# Advancing Textile Waste Recycling: Challenges and Opportunities Across Polymer and Non-Polymer Fiber Types

**DOI:** 10.3390/polym17050628

**Published:** 2025-02-26

**Authors:** Mehrdad Seifali Abbas-Abadi, Brecht Tomme, Bahman Goshayeshi, Oleksii Mynko, Yihan Wang, Sangram Roy, Rohit Kumar, Bhargav Baruah, Karen De Clerck, Steven De Meester, Dagmar R. D’hooge, Kevin M. Van Geem

**Affiliations:** 1 Laboratory for Chemical Technology, Department of Materials, Textiles, and Chemical Engineering, Faculty of Engineering and Architecture, Ghent University, Technologiepark 121, 9052 Zwijnaarde, Belgium; mehrdad.seifali@synpet.com (M.S.A.-A.); bahman.goshayeshi@ugent.be (B.G.); oleksii.mynko@ugent.be (O.M.); yihan.wang@ugent.be (Y.W.); sroy@bitmesra.ac.in (S.R.); rohitkumar.rohitkumar@ugent.be (R.K.); bhargav.baruah@ugent.be (B.B.); dagmar.dhooge@ugent.be (D.R.D.); 2 Synpet Technology, R&D Center, Avenue Louise 523, 1050 Brussels, Belgium; 3 Centre for Textile Science and Engineering, Department of Materials, Textiles, and Chemical Engineering, Faculty of Engineering and Architecture, Ghent University, Technologiepark 70a, 9052 Zwijnaarde, Belgium; brecht.tomme@ugent.be (B.T.); karen.declerck@ugent.be (K.D.C.); 4 Laboratory of Petrochemical Technology (LPT), Department of Chemical Engineering, Aristotle University of Thessaloniki, University Campus, 54124 Thessaloniki, Greece; 5 Laboratory for Circular Process Engineering (LCPE), Department of Green Chemistry and Technology, Ghent University, 8500 Kortrijk, Belgium; steven.demeester@ugent.be

**Keywords:** textile waste, end of life, sustainability, reuse, mechanical recycling, chemical recycling

## Abstract

The growing environmental impact of textile waste, fueled by the rapid rise in global fiber production, underscores the urgent need for sustainable end-of-life solutions. This review explores cutting-edge pathways for textile waste management, spotlighting innovations that reduce reliance on incineration and landfilling while driving material circularity. It highlights advancements in collection, sorting, and pretreatment technologies, as well as both established and emerging recycling methods. Smart collection systems utilizing tags and sensors show great promise in streamlining logistics by automating pick-up routes and transactions. For sorting, automated technologies like near-infrared and hyperspectral imaging lead the way in accurate and scalable fiber separation. Automated disassembly techniques are effective at removing problematic elements, though other pretreatments, such as color and finish removal, still need to be customized for specific waste streams. Mechanical fiber recycling is ideal for textiles with strong mechanical properties but has limitations, particularly with blended fabrics, and cannot be repeated endlessly. Polymer recycling—through melting or dissolving waste polymers—produces higher-quality recycled materials but comes with high energy and solvent demands. Chemical recycling, especially solvolysis and pyrolysis, excels at breaking down synthetic polymers like polyester, with the potential to yield virgin-quality monomers. Meanwhile, biological methods, though still in their infancy, show promise for recycling natural fibers like cotton and wool. When other methods are not viable, gasification can be used to convert waste into synthesis gas. The review concludes that the future of sustainable textile recycling hinges on integrating automated sorting systems and advancing solvent-based and chemical recycling technologies. These innovations, supported by eco-design principles, progressive policies, and industry collaboration, are essential to building a resilient, circular textile economy.

## 1. Introduction

Textiles are an integral part of our society because clothes provide comfort, protection, and temperature regulation to people, while domestic textiles provide utility around the house, and technical textiles play crucial roles in their respective industries [1,2]. These industrial applications may even have positive effects on sustainability, such as textiles used for wastewater treatment and material recovery [3,4,5,6,7,8,9,10,11]. While the global significance of textiles is unquestionable, the sustainability of the textile sector is still far from optimal [12,13].

A sustainable industry can be defined as one that provides for the needs of the current generation without compromising future generations’ ability to do the same, smartly integrating material usage and the environmental impact of production [14,15]. More generally, three aspects of the industry can be looked at: the economic, social, and environmental aspects. This is the so-called “triple bottom line” framework coined by John Elkington [16] in 1994.

When it comes to the economic aspect, the textile industry is a global giant. The worldwide production of textile fibers reached 113 million tons in 2021—up from approximately 24 million tons in 1976—and continues to grow at a consistent rate of 3.4% [17]. This represents a vital fraction of the world’s economy, with textiles representing 4.19% (USD 882 billion) of the world’s total trade in 2021 as the seventh most-traded product [18]. This is expected to grow to USD 1320 billion by 2030 [19]. Within Europe, this industry’s largest subsectors are clothing and accessories (37%, including workwear), industrial and technical textiles (17%), fabrics (15%), and home textiles (14%) [20].

Textile production is a source of employment for millions of people. While the exact number of employees in the textile industry is unknown, conservative estimates yielded 430 million in 2017, including those who produced or harvested the raw materials necessary for the sector [21]. The World Bank estimates the global labor force to be 3.36 billion people this year, meaning that approximately one out of every eight workers worldwide is affiliated with the textile industry [22]. While this employment is a huge boon to the social aspect of the industry, there are also many concerns regarding related issues, such as worker abuse, low wages, gender equality, child labor, worker health and safety, and a high environmental cost [23,24].

The environmental aspect is associated with the mass production of textiles because the textile industry is ranked the fourth most environmentally damaging sector in the world [25,26]. In 2020, it had the third-highest impact on land use after the food and housing sectors [27], and it consumes enormous amounts of resources. In 2015, the industry used over 96 million tons of non-renewable resources, such as oil, fertilizers, dyes, and finishing chemicals, which will grow to an estimated 295 million tons by 2050 if no changes are made [28]. Additionally, 79 billion m^3^ of freshwater were used, with up to 2700 L of water required to produce a single T-shirt [29]. This freshwater use, particularly for the dyeing and finishing of textile products, made the sector responsible for approximately 20% of global clean water pollution [25]. Annually, around 0.5 million tons of microfibers from the washing of synthetic textiles are also discharged into water bodies, with synthetic clothing production responsible for 35% of the primary microplastic pollution in the environment. A single load of laundry for polyester clothing can release up to 700,000 microplastic fibers, which can ultimately enter the food chain [30]. Other emissions are just as large, with the textile industry accounting for approximately 10% of the world’s total greenhouse gas (GHG) emissions [31,32].

It is important to note that both industry and consumers are responsible for the aforementioned environmental statistics. From 2000 to 2015, for example, clothing production doubled from 50 billion units to over 100 billion units, not solely due to a growing world population and gross domestic product, but disproportionately due to the concept of “fast fashion” [28]. This consumerist trend entails clothing collections changing much more frequently and people wearing each garment fewer times before discarding it. Within those 15 years, the average clothing utilization dropped by 36% across nearly every apparel category worldwide, although this varies strongly by country, with China seeing a utilization rate drop of 70% and US citizens wearing garments for just a quarter of the global average [28]. It is estimated that more than half of all fast fashion clothing is discarded within one year of purchase [33]. In addition to social education on fashion culture, a fundamental revolution should also be considered in the recycling industry and in textile disposal methods to maximize textile recovery in different ways.

In the initial stage, progress can be initiated by implementing separate bins for textiles in households and public spaces [34,35]. Currently, textile waste is mainly manually sorted, with some types being exported or reused, while the remaining textiles are discarded. This initial phase with better textile sorting is one of the basic steps of recycling but can currently be time-consuming and expensive because mixed fibers and composites are used in several textiles. As such, this initial stage is difficult, requiring combined recycling processes [36,37,38]. To overcome limitations in the current manual sorting system, the automatic sorting of textiles could be utilized. Many techniques are currently being developed to address the separation of different textiles [39].

After sorting the design, we should further improve the various methods that have been developed to recycle textiles. These include physical recycling [40,41,42], chemical recycling [43,44,45] (differentiating between solvolysis [46], pyrolysis [47,48], and gasification [49], all aiming at different small chemical products depending on the type of process and textile waste [50]), biological recycling (e.g., enzymolysis and fermentation) [51], composting [52], and mechanical recycling [53] (in which the polymer structure of the textile is preserved). Mechanical recycling techniques may involve fiber, yarn, and/or fabric recycling, or the conversion of textiles into primary raw materials through dissolution and/or re-extrusion to enable fiber production or for other purposes [54,55,56,57].

In general, numerous studies have explored various methods of textile recycling. In this paper, we aim to identify appropriate recycling techniques for different types of textiles based on their chemical and physical structures [44,58,59,60]. In what follows, we first discuss the main textile polymer types and their blends, as well as their manufacturing, to highlight how advances in sorting and recycling technologies can improve textile waste management. The review is completed with a life cycle analysis (LCA) section to further help the reader identify the environmental dimensions of textile waste.

## 2. Raw Materials, Chemical Principles, and Manufacturing of Textiles

**Figure 1:**
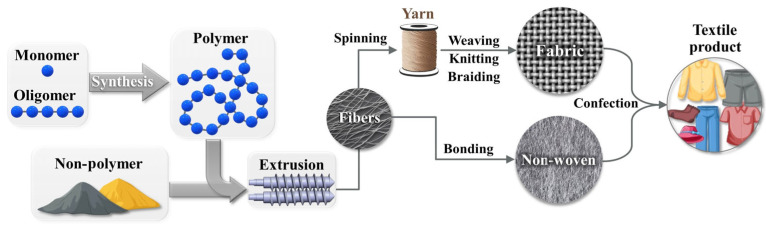
The textile manufacturing chain, from building block to end product.

To understand the different end-of-life options for textile products, it is important to know how they are built because recycling processes follow this same structure, but backwards. As illustrated in Figure 1, textiles start their life cycle as non-polymers, naturally occurring polymers, or building blocks that are synthesized into synthetic polymers. These polymers can then be extruded into fibers, although naturally occurring fibers that do not need to be extruded also exist, such as cotton or wool. Whatever their composition, many fibers can, at a further stage, be combined by spinning them into yarns to then make fabrics via different processes (weaving, knitting, braiding, etc.), or they can be bound (e.g., by adhesives) and turned into nonwoven fabrics. Final processing into a finished textile product involves coloration and finishing treatments [61,62,63].

In the following sections, the main categories of fiber production, as well as their embedding in textile manufacturing processes, are elaborated on.

### 2.1. Fiber Production Routes

**Figure 2:**
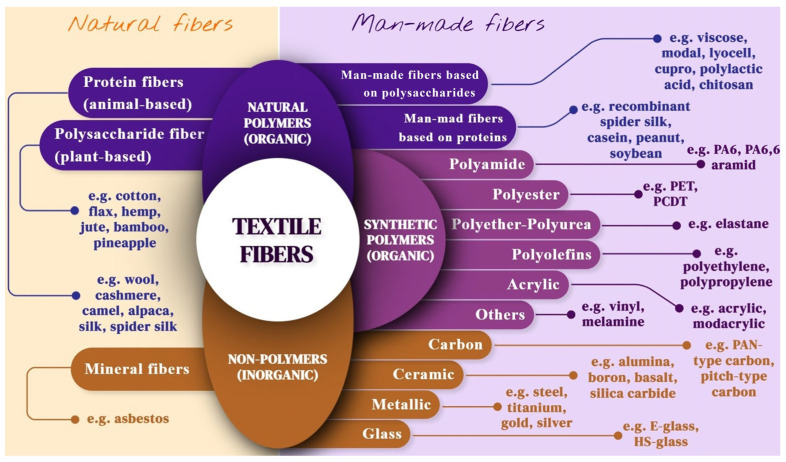
Fiber categories based on polymer and fiber types.

Fibers can be naturally occurring or man-made. Historically, textiles were produced from naturally occurring fibers, such as animal wool, cotton, silk, various types of cellulosic products, and asbestos [64]. However, the emergence of commercial synthetic polymers and advanced fiber production processes has led to the widespread usage of man-made fibers, such as those based on the synthetic polymers polyester, polyamide, polyolefins, and polyurethane (PU), man-made natural polymers (e.g., viscose and lyocell), and non-polymers (e.g., carbon and glass) [65,66]. Examples of these three categories are shown in Figure 2.

**Figure 3:**
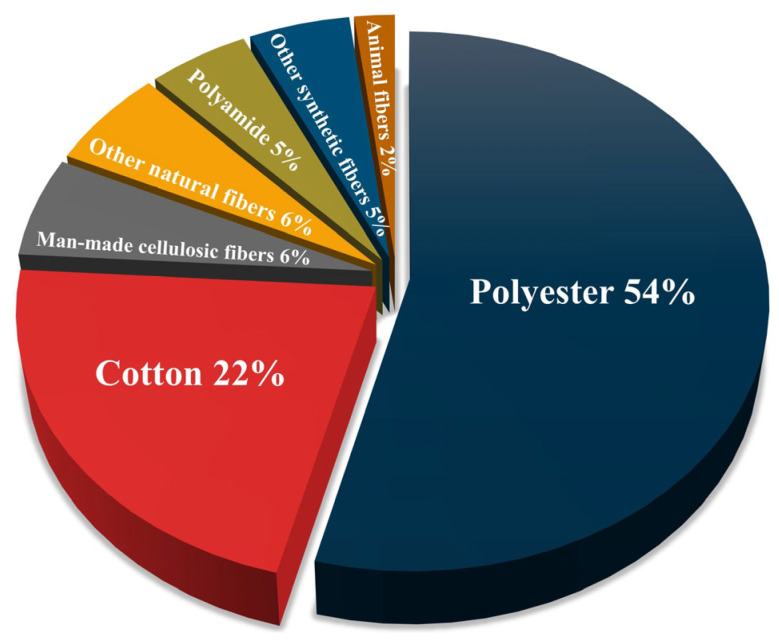
Worldwide distribution of fiber extraction and production. Natural fibers represent 30% of the total, while man-made fibers represent 70% [17].

Global fiber production has reestablished a growing trend after a slight decline in 2020 due to the coronavirus disease 2019 (COVID-19) pandemic. In 2022, a record 116 million tons of fibers were produced, which is expected to reach 147 million tons by 2030 if business continues per the current standard [67]. Among them, polyester fibers accounted for an overwhelming majority of 54% of the net produced fibers, followed by cotton at 22%. Man-made cellulosic fibers (MMCFs) and polyamide fibers are the next two most important fibers, accounting for 6% and 5% of global fiber production, respectively. Animal-derived fibers accounted for only 2% of the total, while other natural fibers accounted for 6%, and other man-made fibers of synthetic polymers (e.g., polyolefins and polyacrylics) accounted for 5% (see Figure 3) [17]. Besides these commonly used fibers produced in large quantities, there are unique industrial fibers produced from both organic and inorganic materials, such as optical poly(methyl methacrylate) (PMMA) fibers [68], aramid fibers, and carbon fibers [69], which serve specialized industrial applications and are produced in limited quantities (not included in Figure 3).

#### 2.1.1. Natural Fibers

Natural fibers occur in their fibrous structure in nature and can be extracted for industrial use. They are mostly made up of natural polymers produced by plants and animals, but there are also non-polymer mineral fibers that occur naturally through geological processes [70].

##### Natural Fibers of Natural Polymers

Natural fibers of natural polymers can be categorized into polysaccharide fibers, which are plant-based, and protein fibers, which are animal-based. Polysaccharide fibers consist of monosaccharide units linked by glycosidic bonds [59]. By far the most important polysaccharide in the textile industry is cellulose, which is composed of D-glucose monomers linked by β-(1,4)-glycosidic bonds [59]. Cellulose is a main constituent of plant cell walls and is found in many different natural plant fibers: seed fibers (e.g., cotton and kapok), bast fibers (e.g., flax, hemp, jute, and ramie), leaf fibers (e.g., pineapple and sisal), fruit fibers (e.g., coir), and grass and reed fibers (e.g., bamboo) [71]. In addition to cellulose, these fibers may contain traces of water, waxes, pectins, and fats [72]. Of the aforementioned plant fibers, cotton is the most used, with a share of 22% of the global fiber market in 2021, while all other natural fibers combined had a share of 6% [73]. Worldwide, cotton is produced at approximately 25 million tons annually, utilizing 2.5% of the world’s arable land [74]. After cotton, flax, hemp, and jute are the most common plant-based (cellulose) fibers [75].

Animal-based fibers are primarily composed of collagen or keratin, proteins consisting of amino acid units that are connected by peptide bonds [70]. α-Keratin is the main component of hair fibers (e.g., wool, alpaca wool, cashmere, mohair, and angora wool), while β-keratins—chief among which is fibroin—are the main components of feather and silk fibers (e.g., silk and spider silk). Collagen fibers are mostly found in mammalian skin and tendons and are not used in conventional textile fibers but are the base material for leather [76]. The most important animal-based fibers in the current textile industry are sheep’s wool, camel hair, angora wool, and silk [75].

In general, the natural fibers of natural polymers have a low density and high crystallinity, leading to a high specific strength and modulus. Due to their abundant supply, and renewability in tandem with these mechanical properties, they are used from everyday products, such as clothing, to more advanced products, such as fiber-reinforced polymer composites. An important advantage of these natural fibers is that they are both biodegradable and recyclable at the end of their lives via several recycling processes, except for those that require high temperatures (e.g., melt re-extrusion) because natural polymers degrade at such temperatures [77,78].

##### Natural Fibers of Non-Polymers

The only naturally occurring fibers of non-polymers are asbestos fibers. They are composed of silicate groups that have fully crystallized into long, thin, fibrous crystals, each comprised of even smaller fibrils [79]. There are six types of asbestos, split into two groups: serpentine asbestos and amphibole asbestos [80,81]. Each type consists of silicate (SiO_2_) and some other minerals in its chemical structure: aluminum oxide (Al_2_O_3_), ferric oxide (Fe_2_O_3_), FeO, magnesium oxide (MgO), calcium oxide (CaO), and/or sodium oxide [82].

Asbestos fibers have excellent fire resistance and thermal, electrical, and sound-insulating properties, leading to their main use in the construction sector. However, as they have been documented to cause mesothelioma (a type of cancer in the chest and abdominal cavities) and asbestosis (lung scarring due to asbestos fiber inhalation) in people who work with them, they have been banned in approximately 60 countries [83,84].

Asbestos fibers are non-biodegradable and can contaminate waters and the surrounding atmosphere if landfilled improperly. They can be mechanically recycled through heat treatment at temperatures above 1000 °C or through irradiation with microwaves, at which point the hydrate silicate fibers turn into inert Mg-Fe silicate glass, which can be used for the fabrication of traditional ceramic materials [82].

Man-made fibers derived from non-polymers, such as inorganic fibers or fibers made from natural substances like cellulose, are increasingly being explored for various applications. These fibers can offer unique advantages, such as enhanced durability, heat resistance, and biodegradability. They are commonly used in specialized fields like construction, automotive, and medical industries, where their specific properties—like strength, fire resistance, and environmental friendliness—are highly valued. The applicability of these fibers continues to grow as technology advances, offering new possibilities for sustainable and functional materials [85].

#### 2.1.2. Man-Made Fibers

Seventy percent of the fibers used globally do not occur in nature and are produced industrially via melt or solution spinning processes [17]. The basic premise of fiber production is that the polymer material must be liquefied in some way, either via melting or dissolution, after which it can be processed into thin, elongated structures, thus forming fibers [86]. These can be staple fibers with a short length similar to natural fibers or filaments with an “infinite” length (limited only by stopping the spinning process).

Today, thermoplastic polymers are preferably processed via melt spinning over dissolution spinning because melt spinning processes typically yield higher spinning speeds and do not require solvent separation or recovery [87]. For non-thermoplastic polymers, solution spinning is a viable alternative, possibly after pretreatment steps, to allow for suitable solubility for less soluble polymers (e.g., the rayon spinning process). Further exceptions are the emulsion spinning process used for fluorocarbons or the production of certain ceramic materials via a sacrificial polymer solution (e.g., cellulose), which is then burned away, leaving only the sintered mass in fiber form [88].

Melt spinning typically starts with dried polymer chips melted continuously in screw melters to yield a stable, pure, and homogeneous melt. Certain thermoplastic polymers, such as polyester and polyamide, are synthesized in a molten state and can be processed immediately after polymerization without the need to be turned into polymer chips first [89]. In any case, the polymer melt is pushed through the spinneret holes into a quench chamber, in which air (or water) currents remove heat from the liquid protofibers to solidify them. In addition, other melt-based technologies exist, such as centrifuge spinning (e.g., glass), split-film spinning (e.g., polypropylene), melt electrospinning (e.g., polylactic acid), preform drawing (e.g., glass), melt blowing (e.g., polypropylene), and spun bonding (e.g., polyethylene terephthalate [PET]) [90].

The two main solution spinning techniques are dry and wet spinning (including gel spinning). Both extrude a polymer solution through spinneret holes, after which the fiber is formed through the solvent’s evaporation out of the protofiber through the application of heat in a solidification chamber for dry spinning [87,91] or through the use of a coagulation bath containing a non-solvent for wet spinning [87]. More specialized solvent spinning techniques include solvent electrospinning, centrifuge spinning (e.g., polycaprolactone), and flash spinning (e.g., polyethylene [PE]) [92].

It should be noted that the conventional melt and solution spinning processes include a final shared step. During or after solidification of the protofibers, they are drawn to several times their original length by a series of rolls that rotate at increasing speeds. This aligns the crystalline sections of the fiber structures in a lengthwise direction to improve the fibers’ mechanical properties. To ensure that this process happens smoothly, a finish may be applied to the solidified fibers.

##### Man-Made Fibers of Natural Polymers

Some fibers do not occur in nature but do contain natural polymers. Chief among these are MMCFs, which are created by processing cellulose-containing raw materials, such as wood or cotton waste [93]. MMCFS can generally be categorized into two types: regenerated cellulose and cellulose esters (e.g., cellulose acetate and cellulose triacetate). The primary category, commonly known as viscose, encompasses the majority of production. These fibers are primarily produced using two industrially dominant technologies: the viscose process, which involves the chemical modification of cellulose and its regeneration, and the lyocell process, a direct dissolving process that does not require chemical modification. Both technologies are employed to create fibers with different properties and characteristics.

In general, the use of strong acids and bases during chemical treatments of MMCFs reduces the average molar mass of cellulose, resulting in MMCFs with lower mechanical properties compared to cotton. However, finishing processes, such as calendering [94], sanforization [95], and coating [96], can partially compensate for these lower properties, but cotton still provides higher quality [97,98,99].

Viscose is the most commonly used MMCF [100]. As it is made up of pure α-cellulose, there are many hydroxyl groups on its surface. These functional groups are highly reactive and have a strong interaction with a polar polymer matrix due to hydrogen (H_2_) bonding.

**Figure 4:**
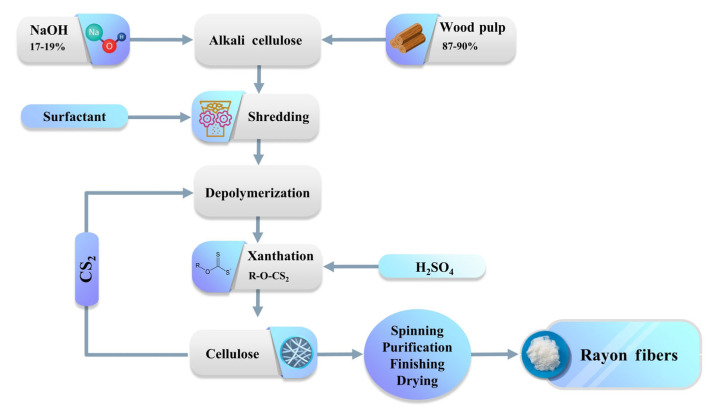
The viscose production process [101,102].

To manufacture viscose (Figure 4), wood pulp is first chemically converted into a soluble compound to obtain pure cellulose. Then, aqueous sodium hydroxide (NaOH) is used to form alkali cellulose and depolymerize cellulose to some extent. Carbon disulfide is added to the alkali solution to form sodium cellulose xanthate. Finally, sulfuric acid (H_2_SO_4_) is used to hydrolyze the alkali solution and regenerate cellulose and carbon disulfide. The quality of wood pulp, the amount of chemicals used, the (net) degree of depolymerization, and hydrolysis influence the properties of the final product [101,102].

**Figure 5:**
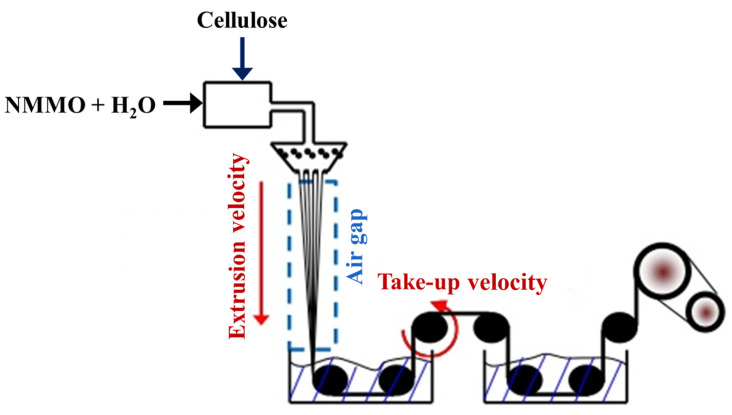
The lyocell production process [109].

Lyocell is a revolutionary technology for manufacturing MMCFs that involves a sustainable and environmentally friendly process. As shown in Figure 5, the process starts with dissolving cellulose, usually derived from wood pulp, in a nontoxic solvent, such as N-methylmorpholine N-oxide (NMMO) [103]. This solution is then extruded through fine spinnerets to create individual filaments. These filaments are washed and dried to remove the solvent, resulting in pure cellulose fibers [104]. The entire process is a closed-loop system, in which the solvent is continuously recovered and reused, minimizing waste and chemical emissions. The resulting lyocell fibers exhibit excellent properties, such as high strength, softness, moisture absorption, and breathability, making them suitable for a wide range of applications, including textiles, apparel, and nonwoven products. Moreover, lyocell is considered a sustainable choice due to its renewable raw material source, low environmental impact, and biodegradability [105].

Besides cellulose, there is another natural polysaccharide used to make man-made textile fibers: chitin and its derivative, chitosan. These polymers are very similar in structure to cellulose, but their glucose monomers are slightly different. For chitosan, the glucose molecule has an amine functional group (glucosamine), and for chitin, this glucosamine has an additional acetyl group attached to the amine group (acetylglucosamine) [106]. Chitin’s biological function is also similar to that of cellulose, making up the cell walls of many living organisms. In this case, it is not found in plants but in fungi and the exoskeletons of certain animals, such as insects and arthropods. Chitosan fibers are made through the deacetylation of chitin with a strong alkali (e.g., NaOH) at high temperatures, and then dissolving the chitosan in an acidic solvent, typically acetic acid (CH_3_COOH), which can be wet spun into a coagulation bath of NaOH or ethanol (EtOH) [107]. Despite the potential for their applications in the textile industry, chitin and chitosan represent less than 1% of the total fiber market, with use only in medical and specialty applications due to their biodegradable and antimicrobial properties [108].

##### Man-Made Fibers of Synthetic Polymers

Synthetic polymers result from chemically converting monomers, oligomers, or polymers that occur in nature or are in turn produced from a petroleum (or alternative) base. Overall, there are two types of polymer synthesis: chain-growth polymerization and step-growth polymerization [110].

**Figure 6:**
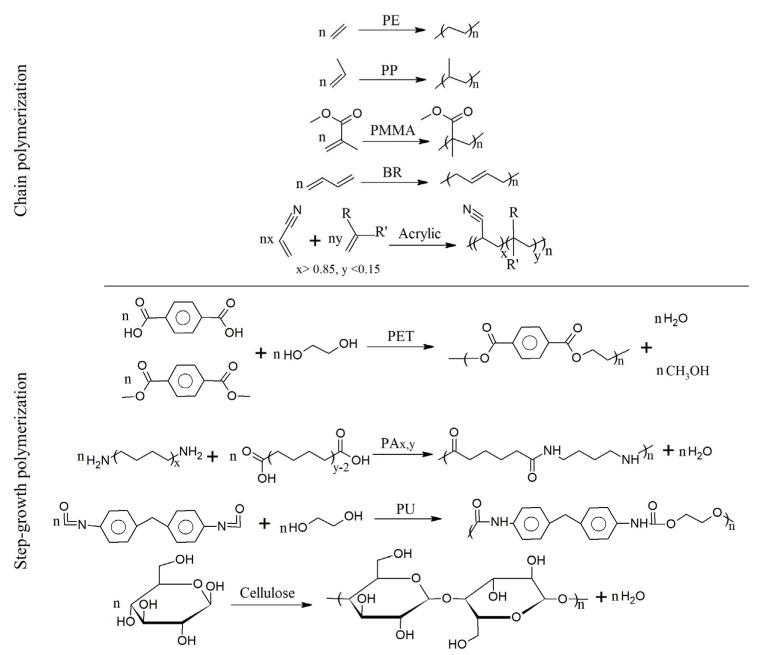
Overall reaction for important polymerizations. The targeted polymer is listed above the arrow for clarity [111,112,114].

Figure 6 displays examples of the overall reaction of various polymerization processes. Chain-growth polymerization [111,112,113] is used to produce vinyl polymers, such as PE, polypropylene (PP), PMMA, butadiene rubber (BR) polymer, and acrylic polymers, while step-growth polymerization [114,115] is used for polymers, such as PET (in general polyesters), polyamide, and PU.

The diverse characteristics of polymers have led to their widespread use in the textile industry, ranging from (poly)acrylic fibers (primarily composed of copolymers of acrylonitrile and other vinyl monomers, such as vinyl chloride) [116] to polyolefin fibers [117], synthetic leather made from polyvinyl chloride (PVC) [118], foam made from PU [119], and rubbers used as adhesives [120]. Additionally, polyvinyl acetate (PVAc) and polyvinyl alcohol (PVA) have been utilized as reinforcement fibers and adhesives [121,122,123].

In the following sections, a more detailed description of PET and polyamide fiber synthesis is provided.

Polyesters

Linear polyesters are manufactured from the condensation polymerization of a bifunctional carboxylic acid with a bifunctional alcohol [124]. PET is the dominant polyester utilized in the textile industry (and for bottle grades), whereas other polyesters are limited to specific applications [125,126,127]. The main features of the important polyesters are shown in Table 1. The chemical structures of polyesters primarily affect their melting temperature (T_m_) and glass transition temperature (T_g_), with a structure that corresponds to a higher T_m_ leading to physical properties such as thermal and mechanical stability, while a structure that yields a lower T_g_ results in increased flexibility and softness [125,128,129]. Semi-crystalline engineering plastics, such as PE, can be oriented and self-crystallized during extrusion, resulting in enhanced superior physical and mechanical properties.

**Figure 7:**
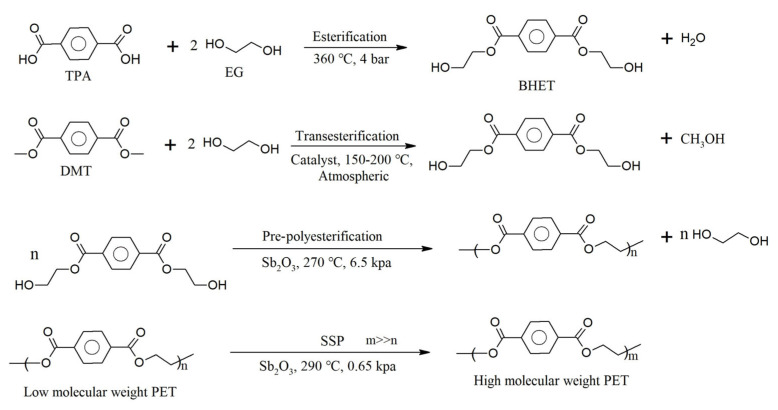
The industrial polymerization and structure of PET [130,131].

The three main stages involved in PET polymerization are depicted as the first three entries in Figure 7. This polymerization initially involves (trans)esterification. For example, esterification between terephthalic acid (TPA) and ethylene glycol (EG) takes place at a high temperature and pressure (360 °C, 4 bar). To prevent reverse reactions, methanol (CH_3_OH) is removed. Bis(2-hydroxyethyl terephthalate) (BHET) is generated, and it serves as the oligomer for the second stage, which takes place catalytically (usually through the presence of antimony trioxide [Sb_2_O_3_]) at a moderate temperature and low pressure (270 °C, 6.5 kPa). Subsequently, the average molar mass is increased to the commercial polymer grade in the third stage, which is carried out under high vacuum conditions that release H_2_O, EG, and light oligomers (290 °C, 0.65 kPa).

A complementary extra stage (the fourth entry in Figure 7) is called solid-state polycondensation (SSP). Through this process, Sb_2_O_3_ remains in the final product, and PET may contain around 170–300 mg/kg antimony (Sb) as well as other metals, such as cobalt (Co), iron (Fe), manganese (Mn), and chromium (Cr). Similarly, liquid-state polycondensation (LSP) involves the removal of by-products at high temperatures during the melting of PET. By using LSP, high-performance PET fibers can be produced directly from the melt, without the need for a spinning process. During both the SSP and LSP processes, the average molar mass can be kept within the desired range by adjusting the pressure, temperature, and catalyst type and amount [130,131].

Intrinsic viscosity (IV) is commonly used as a means of characterizing PET, which provides indirect information on the average molar mass and configuration of the polymer. Typically, PET polymers with a higher average molar mass exhibit higher IV values. PET fiber and bottle grades generally have IV values ranging from 0.57 to 0.62 dL·g^−1^ and from 0.72 to 0.85 dL·g^−1^, respectively [132,133].

**Table 1:** Different types of polyesters used in textile and manufacturing applications [125,128,129].

Polyester	Full Name	Structure	T_m_ (°C) ^1^	T_g_ (°C) ^2^	Assets	Ref.
PET	Polyethylene terephthalate	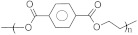	250–265	70–80	A common type of polyester	[134]
PTT	Polytrimethylene terephthalate	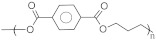	220–230	30–40	Superior softness and drape	[135]
PBT	Polybutylene terephthalate	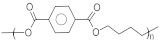	225–235	50–60	Good mechanical properties and resistance to chemicals	[136]
PCDT	Poly(1,4-cyclohexylene-dimethylene terephthalate)	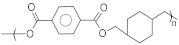	255–270	60–70	High stretch and recovery properties	[137]
PEN	Polyethylene naphthalate	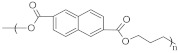	260–275	120–140	High temperature resistance and excellent dimensional stability	[138]
PBN	Polybutylene naphthalate	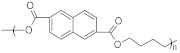	250–265	55–65	High strength, stiffness, and thermal stability	[139]
PLA	Polylactic acid	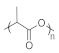	130–180	50–65	A biodegradable polyester derived from renewable resources, such as cornstarch and sugarcane	[140]
PETG	Polyethylene Tere-phthalate Glycol	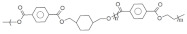	230–260	80–95	Less shrinkage for printed parts	[141]

^1^ T_m_, melting temperature of the bulk polymer; ^2^ T_g_, glass transition temperature of the bulk polymer.

Polyamides

**Figure 8:**
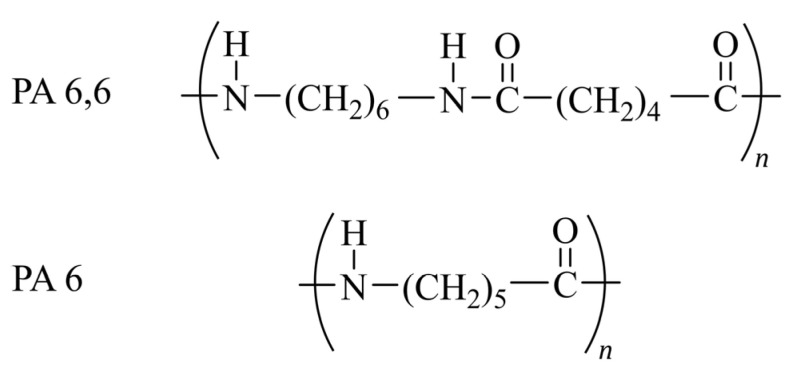
PA6,6 vs. PA6.

Linear polyamide, also known as nylon, is a synthetic polymer formed through the condensation polymerization of a bifunctional amine and carboxylic acid. Polyamide fibers are highly valued for their resilience, durability, and strength, as well as for their flexibility to be molded into various shapes [142,143]. If a monomer containing two carboxyl and amine functions is utilized, the resulting polymer is labeled PAx, where x is the number of carbons in the monomer. Conversely, if two separate units consisting of a bifunctional amine and bifunctional carboxyl are used, the polyamide is called PAx,y, where x and y indicate the number of carbons in the amine and carboxyl units, respectively (Figure 8).

An important polyamide is PA6,6, whose synthesis involves two stages. Initially, adipic acid and hexamethylene diamine are combined in an aqueous medium at a high temperature and pressure (254 °C, 18 bar) for pre-polymerization. Subsequently, the polymerization process is sustained through melt polycondensation at a high temperature (267 °C) and atmospheric pressure to attain an average molar weight within the commercial range [130]. To achieve higher average molar masses, it is essential to remove by-products from the reactor through the LSP and/or SSP process, which helps prevent the kinetic relevance of reversibility and depolymerization reactions.

The various types of polyamides possess unique properties and applications. For instance, PA6,6 is commonly used in fiber production, while PA6 is frequently utilized for injection molding and less often as fibers (e.g., fishing nets) [144].

##### Man-Made Fibers of Non-Polymers

There are four categories of non-polymers that are common in man-made fibers: carbon, glass, ceramics, and metals. All of these building blocks are inorganic, although carbon and several ceramic non-polymer structures (e.g., silicon carbide) are often produced from organic polymers [145,146,147].

Carbon fiber, at its chemical structure level, very similar to graphite, is composed almost entirely of carbon atoms in a regular hexagonal pattern [148,149]. It is a lightweight, high-strength material composed of carbon atoms bonded together in a crystalline structure, offering exceptional mechanical properties. Its engineering applications span the aerospace, automotive, sporting goods, and renewable energy sectors due to its strength-to-weight ratio. It is manufactured by converting precursor materials, such as polyacrylonitrile (PAN) fibers or pitch, into carbon fibers through processes such as oxidation and carbonization [150,151].

Glass fiber, also known as fiberglass, is a versatile engineering material composed of fine fibers made from glass. It exhibits excellent strength, durability, and resistance to heat and corrosion, making it ideal for various applications [152]. While some would call glass an inorganic cross-linked polymer, it is not classified as such due to the inability to distinguish individual polymer chains in the silica network [145,146,147].

### 2.2. Blended Fiber Textiles

Blended fiber textiles are made of yarns composed of two or more different types of fibers (so-called combination yarns) or of two or more different yarns of a single fiber type each (creating a so-called mixed fabric). This blending offers unique properties and advantages that are not found in individual fibers alone [153,154]. Several examples of blended textiles are given below. For example, the polyester–cotton blend is one of the most common and popular mixed textile combinations. The resulting fabric combines the softness and breathability of cotton with the durability and wrinkle resistance of polyester. It is commonly used in various garments, including shirts, trousers, and bedsheets [155,156].

Furthermore, a wool–synthetic blend is a blended wool with synthetic fibers, such as polyamide or polyester, creating a fabric that combines the warmth, softness, and moisture-wicking properties of wool with the strength, durability, and quick-drying characteristics of synthetics. This blend is often used in outdoor clothing, such as jackets and sweaters [157].

The silk–linen blend is known for its strength and breathability. This combination results in a fabric that has a luxurious feel, good moisture absorption, and improved durability. It is commonly used in dresses, scarves, and home furnishings [158].

A viscose–polyester blend is used to create a fabric that combines the comfort, drape, and moisture absorption of viscose with the durability and wrinkle resistance of polyester. This blend is often used in dresses, skirts, and blouses [159,160].

In addition, an acrylic–wool blend is employed to create a fabric that retains the warmth, softness, and insulating properties of wool while reducing its cost and enhancing its resistance to shrinkage and stretching. This blend is commonly used in knitwear, blankets, and accessories [161].

The foregoing are just a few examples of blended fiber textiles. Numerous other combinations are available, depending on the desired properties and application of the fabric. The blending of textiles allows manufacturers to create fabrics that offer a combination of desirable characteristics from different types of fibers [162].

Furthermore, in the textile industry, various additives are used to enhance the performance, appearance, and functionality of fabrics. These additives can be added during the manufacturing process or applied as a finishing treatment [163]. They include dyes and pigments [164], flame retardants [165], antimicrobial agents [166], UV stabilizers [167], softeners [168], water repellents [169], and antistatic agents [170].

The recycling of mixed fibers and additives in the textile industry poses several challenges, such as fiber separation, contamination by including different additives, loss of performance during the recycling process, lack of infrastructure, limited demand for recycled mixed fibers, and lack of standardization [162,171,172,173,174].

### 2.3. Fabrics

Fabric manufacturing encompasses various methods, including weaving, knitting, braiding, and nonwoven techniques, each yielding distinct textile structures. Woven fabrics are created by interlacing two sets of yarns (warp and weft) at right angles on a loom, forming a stable and structured material, such as cotton, silk, or polyester. Knitting involves interloping yarns to produce flexible and stretchable fabrics, such as sweaters or socks [175,176,177,178].

Braiding combines yarns in a diagonal pattern, resulting in the strong and tubular structures used in ropes or cables. Nonwoven fabrics are made by bonding fibers together mechanically, chemically, or thermally, producing materials such as felt or geotextiles. Each production method offers unique characteristics in terms of texture, strength, and suitability for various applications in the fashion, industrial, and technical sectors [179,180].

### 2.4. Textile Products

Textile products encompass a broad array of goods that serve both practical and aesthetic purposes in everyday life and industrial settings. Apparel, ranging from clothing items to accessories such as hats and footwear, represents a substantial segment of the textile market [181].

Home textiles, including bedding, towels, curtains, and upholstery fabrics, are essential for furnishing and decorating living spaces. Technical textiles play vital roles in industries such as automotive, medical, and construction, providing functional solutions, such as airbags, medical textiles, and geotextiles [182].

Sports and outdoor textiles cater to the demands of active lifestyles, with specialized clothing and gear designed for performance and comfort. Industrial textiles serve various industrial applications, including filtration, reinforcement, and packaging, contributing to the efficiency and safety of industrial processes [183].

Industrial textiles include specialized fabrics used in applications such as geotextiles, filtration, protective clothing, automotive components, medical devices, aerospace, and construction materials [184].

The diverse applications and versatility of textile products underscore their importance across different sectors and consumer needs [185].

## 3. End-of-Life Options for Textile Waste

The International Organization for Standardization (ISO) presents the overall environmental aspects of textiles in ISO 5157:2023. It states that the end-of-life stage of textile materials “begins when the used product is ready for disposal, recycling, reuse, etc., and ends when the product is returned to nature (combustion, deterioration), or is recycled or reused” [186]. Before reuse, recycling, or returning to nature can occur, textile waste must be collected and sorted—both at the household and industrial levels—which involves the challenging yet crucial step of separating textiles from other waste fractions. Currently, textile waste is mainly sorted manually, although the use of automated sorting techniques is gaining traction [34,187].

Reuse signifies recovering a product as a whole rather than disassembling it into materials. This product can be lightly damaged and undergo minor reparations as long as it is restored to a usable state, as intended by its original design [186].

Recycling is performed when the product itself cannot be restored but the materials that make up the product can be recovered for use in a new product, usually after some form of pretreatment. As most textile waste consists of polymer materials, ISO 472:2013—the standard on plastics vocabulary—can be consulted for terminology purposes. This standard categorizes recycling methods based on whether changes occur in the chemical structure of the building blocks during the recycling process. It recognizes two categories: mechanical recycling, during which the chemical structure of the material does not significantly change; and chemical recycling, during which the chemical structure of the material significantly changes, such as polymers that are converted into monomers or oligomers [188].

Note that the textile-specific standard ISO 5157:2023 instead categorizes recycling methods based on the process used and has three categories: mechanical, thermomechanical, and chemical recycling [186]. In this review, a broader plastic vocabulary is used. The thermomechanical recycling processes that involve melting polymers mentioned in ISO 5157:2023 then become a subset of mechanical recycling.

Alternatively, recycling can be categorized by the level to which the waste textiles are degraded before they are turned into new products (i.e., fabric, yarn, fiber, polymer, oligomer, or monomer) [189]. Fabric, yarn, fiber, and polymer recycling all employ mechanical recycling techniques, while oligomer and monomer recycling employ chemical recycling techniques [190].

Another classification is that of primary, secondary, tertiary, or quaternary recycling, which is used by the American Society for Testing and Materials in definition D5033 [191]. Here, primary recycling indicates a process whereby recycled materials (also called secondary raw materials) are used to create a product identical to the original product (before it became waste). Secondary recycling turns product waste into a new product that is not directly related to the original product. Tertiary recycling turns waste into basic chemicals or fuels. Quaternary recycling turns waste into heat [192]. The latter is another name for incineration or combustion and does not conserve any materials; thus, it is incorrect to call it recycling and should instead fall under “returning to nature” in ISO 5157:2023. Directive 2008/122/EC of the European Parliament and of the Council also states that “recycling […] does not include energy recovery and the reprocessing into materials that are to be used as fuels or for backfilling operations” [193]. This clearly demarcates the border between what ISO 5157:2023 considers recycling and what it considers “returning to nature”, the latter being any process that recovers only energy or fuels but not materials, or where nothing is recovered at all (e.g., landfilling) [194].

Furthermore, recycling can be categorized as closed-loop recycling, whereby the original product is reformed (identical to primary recycling), or open-loop recycling, whereby the waste is turned into a product that differs from the original product (this can be secondary or tertiary recycling). Within open-loop recycling, a distinction is made between upcycling, side-cycling, and downcycling depending on whether the new product is of higher, equal, or lower quality than the original product, respectively [189].

**Figure 9:**
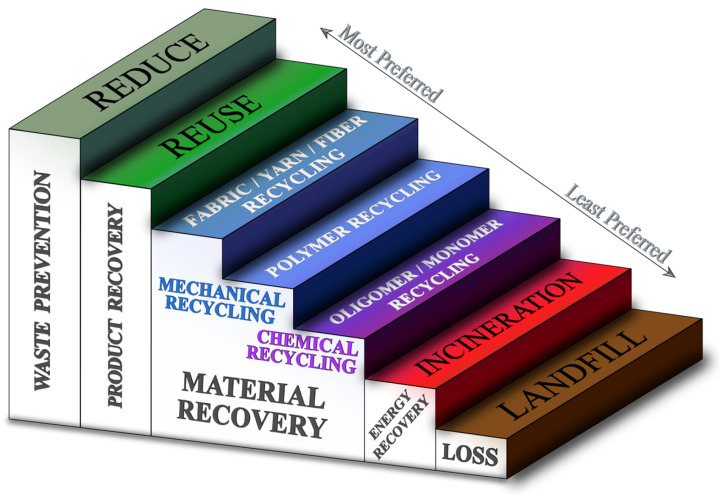
Waste hierarchy staircase for textile end-of-life options. Ideally, processes at the top are used until they no longer yield a product or secondary raw material of sufficient quality, in which case a process lower down should be used, except for incineration or landfill.

It is important to realize that all the mentioned end-of-life options for textile waste—other than landfilling—have some value, but that value is not always equal. To choose the best end-of-life option for a certain waste product, the costs and benefits of each option should be evaluated. In Figure 9, a staircase is shown with all the end-of-life options ordered from top to bottom. The European Union (EU) calls this the “waste hierarchy” in their Waste Framework Directive (Directive 2008/98/EC) [195]. Generally, the higher up on the staircase an option is, the lower the environmental impact and economic cost of that process, due largely to the lower number of production process steps required to return to a recycled end product. The flip side of this coin is that the less costly processes also have a more limited impact on the quality range of the secondary product. Reused products essentially have the same quality as the original products before the latter became waste at the end of their lives, while chemically recycled products are reprocessed so thoroughly that they will often be of similar quality to virgin products.

In an ideal world, any time a product reaches the end of its life—whether that was its first life (i.e., the product was virgin) or a later one (i.e., the product had already been reused or recycled at least once)—one would opt for the highest level on the staircase in Figure 9 as long as that yields a secondary product of sufficient quality. As such, optimal product and material recovery can be achieved by cascading down the staircase [189]. In theory, the bottom two end-of-life options (incineration and landfill) should never be taken because chemically recycled materials should be of equal quality to virgin materials, meaning that process can be repeated infinitely. Incineration and disposal do not recover any materials and thus do not contribute to a closed-loop economy, meaning they should be resorted to only if the reuse or recycling process is impossible for the product or material in question [196].

**Figure 10:**
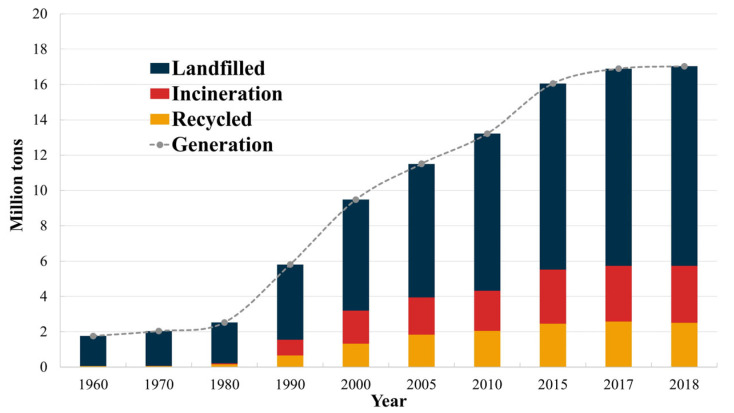
Textile waste management in the U.S. (1960–2018) [197].

Historically, however, most textile waste has been dealt with through landfill disposal or incineration due to its low operating cost, despite its lack of product or material recovery. Only in recent decades—in light of environmental concerns—has this very linear model been challenged and has the closed-loop economy become a focal point. However, there is still a lack of mature, efficient recycling technologies and supported initiatives, meaning that the textile industry still scores badly in terms of sustainability. This evolution can be seen in Figure 10, which illustrates waste management in the US from 1960 to 2018, excluding textiles that are reused or that illegally end up in nature. The trends show that until the 1980s, waste textiles ended up primarily as landfills. Afterwards, textile incineration and recycling gradually increased. In 2018, textile waste management consisted of 15% recycling, 19% incineration, and 66% landfill [197]. Wool is a natural fiber primarily composed of the protein keratin, which is found in the hair fibers of animals such as sheep, alpacas, goats (cashmere), and other mammals [198,199,200].

The aforementioned definitions and statistics on textile waste management highlight the need for accelerated recycling of textiles because current methods are inadequate in addressing true environmental challenges [201,202]. Subsequently, feasible solutions for achieving complete textile waste recycling are reviewed in the following sections, including collecting, automated textile sorting (ATS), and different types of textile recycling technologies [203,204].

### 3.1. Collection and Sorting

#### 3.1.1. Collection

Textile waste is categorized as pre-consumer waste or post-consumer waste depending on its source [205]. Pre-consumer waste is generally easy to collect and can be subdivided into (i) production waste, which is generated during the product manufacturing process [206], (ii) post-industrial waste, which is generated by non-textile manufacturing industries that simply use textiles as inputs or outputs [207], and (iii) finished products that become damaged or remain unsold in retail establishments [208].

Production waste from a textile product can range from 10% to 50% of the raw material used to manufacture it but can easily be collected by the company internally. This makes it the most collected type of textile waste [206].

In theory, so-called post-industrial waste is also easy to collect separately from municipal waste by setting up waste collection schemes between the textile and non-textile industries. In practice, however, this waste is often not collected separately because the collection creates logistical difficulties, and much of the waste ends up being so contaminated that it can no longer be reused or recycled, such as medical textiles used in hospitals [207].

The unsold or lightly damaged finished products found in store inventories (most often clothing and footwear) are extremely suitable for reuse and should be collected and recovered as such. Once again, most of these products are not collected for reuse; instead, they are destroyed to avoid collection, sorting, and possible recycling costs, to avoid devaluing the brand value, and to avoid paying inventory taxes [209]. In 2023, the EU agreed upon a resolution to ban the destruction of unsold clothing, which could help in the sustainable collection of this waste category if the resolution can be enforced, although this would still not prevent companies from exporting their waste outside the EU first [210].

It should be further highlighted that post-consumer or household textile waste is more difficult to collect because it comes from many individual sources and must be separated from regular municipal waste to be of use [211]. A large portion of textile waste is currently still collected without separation from municipal waste. For example, in 2023, the EU generated 13.9 million tons of post-consumer textile waste (approximately 12 kg per person) but collected only 22% of it in a way that would enable reuse or recycling. To improve these numbers, EU legislation will require member states to collect textile waste separately from municipal waste starting in 2025 [195].

Separation from other municipal waste can occur by having charitable organizations or retail stores take in waste textiles, or by installing separate collection bins for textile waste and incentivizing consumers to use such bins. In general, the most important factor for collection rates is consumer convenience, which is geographically dependent, with collection bins being more accessible in more developed regions [205,212].

Recurring problems for post-consumer textile waste collection are unclear regulations for collecting organizations (including the definition of what is and is not classified as waste) and a lack of control over permits for collection bins. Additionally, a lack of sanctions for incorrect collection can lead to illegal textile containers and a lack of transparency throughout the collection process [213]. In addition, (domestic) high-grade recycling possibilities are still scarce for most textile types, meaning that waste textiles generally have a low value, especially because the incineration costs for any collected but unused or unrecycled material are high [213]. For the few recyclable textiles, product saturation can also be a concern, making the diversity of textile waste collection important [214].

Fortunately, modern technologies, such as the Internet of Things (IoT), big data analysis, and blockchain technology, can make textile waste collection more sustainable. Waste products, collection bins, and collection vehicles can all be linked in an IoT network, such as by incorporating smart tags (e.g., radio-frequency identification [RFID] tags) into textile products and suitable sensors into waste containers and collection vehicles. This allows collection services to track the amount, type, location, and even condition of textile waste that is deposited into collection bins or currently loaded onto collection vehicles. These big data can then be fed into predictive algorithms and decision-making tools to optimize collection routes both ahead of time and in real time, increasing collection rates and reducing transportation costs (both financial and ecological) [215,216,217]. If these data are transferred through a blockchain accessible by all stakeholders, they can also improve the traceability, transparency, and accountability of the waste collection process, thus even minimizing illegal dumping and collection [206].

Further down the line, smart contracts can be set up between waste collection services and textile recyclers to automate transactions and to ensure that the quantity and quality of waste being delivered are correct without the need for labor-intensive manual verification and paper-based documentation [218]. Even consumers can be involved by supplying mobile apps and platforms that provide information (and thus awareness), incentives for correct disposal, and a feedback option. These tasks would also allow consumers to request, schedule, track, and pay for individualized services [206].

The downside to the aforementioned technological aids is that they all require specific infrastructure, good connectivity, and security for all users. While possible, these are accompanied by extra maintenance costs and a need for regulation from higher-level platforms [206,218]. Additionally, due to the little manual involvement in this automated collection system, there can be more contamination in the collected textiles, which can cause issues down the line [213]. The current technology is also still maturing in this field, and certain elements, such as cheap, waterproof, and flexible sensors for collection bins, are not yet available [219].

It is important to note that a large amount of textile waste collected in developed countries is exported to developing countries. In 2019, for example, almost 1.9 million tons of waste clothing collected in the EU were exported mostly to countries in Africa (e.g., Tunisia, Ghana, Cameroon, and Togo), Asia (e.g., Pakistan, the United Arab Emirates, and India), or Europe (e.g., Ukraine). This represents 25% of the textiles consumed each year in the EU and marks a large increase from the approximately 600,000 tons exported in 2000. Most of this waste becomes untraceable for the origin country and is not necessarily recovered; used textiles in Africa often get reused locally, while the waste in Asia is mostly downcycled into rags or fillers, but a large portion of the waste streams is likely to end up in open landfills [220]. More recently, many Asian and African countries have started banning the import of waste clothing to boost their domestic textile industries and reduce the environmental impact of that waste ending up in landfills [221].

#### 3.1.2. Sorting

While sorting is not required for pure waste streams, it is required for all mixed waste streams, such as post-consumer textile waste. By sorting, textile waste of different colors, chemical compositions, sizes, and qualities can be separated [108,110]. For example, the quality of a waste product can determine whether it is fit for reuse, must be recycled, or needs to be incinerated or disposed of [222]. Chemical composition is very important for recycling processes because these processes are tuned to specific fiber types, and color is similarly important for recycling without prior decolorization because the secondary raw materials resulting from such recycling processes will end up having the same color or a blend of the input colors [223,224,225].

Currently, textile waste is mainly sorted manually. This is cheap in developing countries where labor costs are low, and it allows for a good estimation of quality, particularly whether a waste product is reusable or must be processed further. However, there are clear downsides to manual sorting. It is very time-consuming and cannot efficiently deal with high volumes of textile waste nor separate waste based on chemical composition other than by relying on product labels, which can be faulty or missing [36,37,38,203]. In addition, workers often experience less evident working conditions, which leads to unreliable sorting [226].

To overcome these limitations, ATS is being developed. To be effective, this sorting system must be nondestructive, able to distinguish qualitatively between fiber types, able to quantitatively analyze fiber blends, and have high throughput [227]. The focus is placed on the separation of waste by color and chemical composition because ATS systems currently cannot distinguish quality differences to the level where they can sort reusable from non-reusable textiles [213].

Physical sorting methods, such as sorting based on density or melting point, can be used in an ATS system. Melting point indicators are considered by some to be inexpensive [214] while others consider them expensive [226], but in any case, these physical sorting methods are rather slow, require rigid training from the operator, and have limitations on the chemical composition they can distinguish (e.g., melting point indicators cannot distinguish PA6,6 from polyester fibers) [214,226].

A promising sorting method for the future is the use of RFID tags in textile products. These tags can contain information on the product (e.g., its chemical makeup, color, and age), which can be read remotely by an in-line sensor in a sorting facility. Unfortunately, these RFID tags are currently used only for clothing and often fail after several laundry cycles [228].

A concept similar to RFID tags is the exploitation of photonic crystals, as described by Lezzi et al. [229]. These polymeric photonic crystals can be introduced into textile fibers and exhibit characteristic infrared reflectance responses in the 1–5.5 μm wavelength range, which can be read by spectroscopes at different angles, thus acting as an information tag within the material itself.

**Figure 11:**
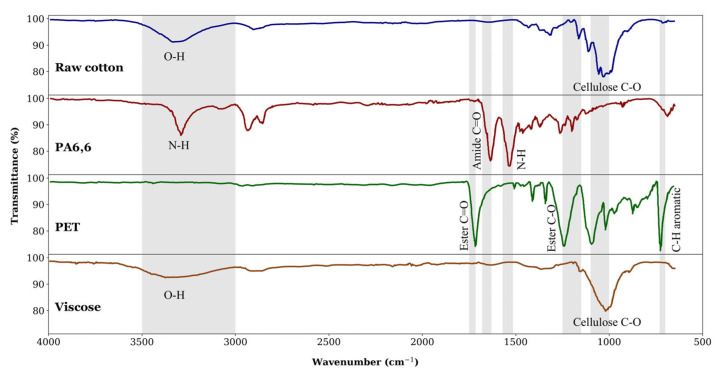
Example of the attenuated total reflection–Fourier transform infrared (ATR-FTIR) spectra of common fibers [231,232,233,234].

While RFID tags and photonic crystal technology are still being developed, the focus of the literature on ATS today is on spectroscopic methods, an overview of which is given in Table 2. Color can easily be detected by a computer system using sensors that pick up reflected visible light (wavelengths roughly between 400 and 700 nm). However, to detect chemical composition, light in the infrared wavelength domain is required. State-of-the-art research on textile sorting is mainly concerned with NIR spectroscopy, which uses wavelengths between 800 and 2500 nm [226,230], but also sometimes studies mid-infrared (MIR) spectroscopy, which encompasses the wavelength range of 2500 nm–25 μm [24,231,232,233,234,235]. Occasionally, visible near-infrared (VNIR) spectroscopy is mentioned, which is a combination of visible light and NIR light with wavelengths between 400 and 1000 nm. Various types of textiles, such as cotton, wool, and polyester, possess distinct spectral signatures that can be detected in this range [236]. An example of chemical-composition identification via infrared spectroscopy is shown in Figure 11. Here, ATR-FTIR spectroscopy has been used in the MIR domain.

For the actual sorting and classification of any identified fiber type, advanced machine learning software tools can be used for pattern recognition and to process the collected infrared data [203,237]. Typical examples are also listed in Table 2.

Furthermore, a study conducted by Riba et al. [24] aimed to enhance textile sorting using an automatic sensing and sorting approach, which involved analyzing 350 well-defined textile samples composed of a single fiber type. The ATR-FTIR spectrum was utilized in combination with various algorithms, including principal component analysis (PCA), k-nearest neighbors (k-NN), and canonical variate analysis (CVA). The outcomes demonstrated that unknown samples could be sorted automatically with high speed and excellent accuracy without the need for any prior analytical treatment.

Chen et al. [238] used NIR with the partial least squares (PLS) and extreme learning machine (ELM) algorithms to categorize textile fibers. According to the findings, the ELM-based predictive models showed higher accuracy than those generated by PLS while having a similar computational cost. Similarly, Liu et al. [239] utilized various machine learning algorithms, including a support vector machine (SVM), multilayer perceptron (MLP), and convolutional neural networks (CNNs), in combination with the NIR spectrum, to classify waste textile fibers. CNN was claimed to demonstrate a higher classification rate, ranging from 92% to 98%.

Zhou et al. [230] utilized NIR spectroscopy in combination with PCA, soft independent modeling by class analogy (SIMCA), and latent Dirichlet allocation (LDA) algorithms to identify seven common, well-defined fibers rapidly and precisely. Their findings revealed that cotton, lyocell, PP, and PET could be efficiently classified, with an almost perfect recognition rate. However, wool, cashmere, and PLA had overlapping features in their chemical and physical analyses, resulting in lower recognition rates when the SIMCA algorithm was utilized. However, through the utilization of LDA and some modifications, the fibers could be completely classified. Consequently, the NIR spectroscopy method utilizing various algorithms has demonstrated the efficient and accurate classification of many important types of textile fibers.

The aforementioned studies show that spectroscopy techniques have many advantages for use in ATS because they can be performed in real time with good throughput while being nondestructive methods [228]. Combining visual and infrared light can yield very accurate detection of not just fiber types but also dyes, thin coatings, and finishes at the surface of such fibers [206]. A drawback of the discussed spectroscopic technologies, however, is that they allow for the identification of only one point in the sample, which is often not good enough when it comes to complex heterogeneous samples, such as textile waste [240].

A possible solution to this problem is hyperspectral imaging (HSI), which combines both spatial and spectral scanning. Essentially, a two-dimensional scan of the waste sample is taken, and at each point (pixel) on the surface, an infrared scan is performed to generate its spectrum. This allows for the detection of multiple fiber types or contamination on a waste sample [240]. For example, Jiang et al. used HSI in the NIR range to identify contamination in cotton lint samples, with 90% accuracy [241]. Blanch-Perez-del-Notario et al. reported that HSI in the visible-NIR range can be used to identify wool and polyamide samples with high accuracy while losing some accuracy on cotton, viscose, and PET samples, especially in the case of fiber blends. Importantly, the system can discriminate between denim and non-denim fabrics of the same color, which is otherwise difficult to distinguish for ATS systems [236].

While HSI allows for the identification of components in heterogeneous waste at both high accuracy and speed [242], it shares a major limitation with more conventional spectroscopic techniques: a lack of depth perception due to the low penetration of visible and infrared radiation. All spectroscopic techniques can characterize only the surface layer of a waste sample. Thus, textiles with thicker coatings or multilayers cannot be identified by infrared spectroscopy, and even functional finishes can pose problems for sensors [203]. Moreover, infrared technology can be costly, particularly for large-scale ATS, because regular calibration of infrared sensors and cameras is necessary to maintain the requested high sensitivity and proper functioning, and extensive spectral libraries are required to analyze the gathered data. Fortunately, these libraries can also be built using other analytical techniques, such as solid-state nuclear magnetic resonance [227]. On the processing side, fast computers with a large data storage capacity (doubly so for HSI) are required. Any noise or interference encountered during the infrared scanning process can also have a substantial detrimental effect on neural network accuracy [206].

Despite the foregoing minor issues with spectroscopy-based ATS, the main obstacle and practical challenge is the complexity of textile waste, including the (multiple) presence of dyes, finishes, buttons, zippers, and prints, but mostly the increasing prevalence of mixed textiles consisting of different fiber types, including those products where different fiber-type threads are used for the seams [227]. While HSI could be an early contender for tackling this problem, dealing with mixed textiles in the future will certainly demand additional resources and efforts from the scientific community.

**Figure 12:**
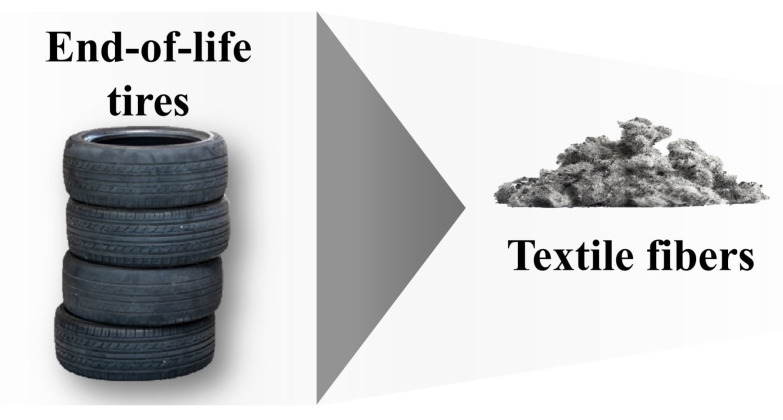
Tire textile recovery [247].

For completeness, additional emphasis is placed on the fiber recovery of tire and rubber products (Figure 12). With an annual production of approximately 60 million tons of tires and rubber products, the textile sector accounts for roughly 5% of this total, which equates to the use of approximately 1–1.2 million tons of fibers primarily made of PET and, to a lesser extent, polyamide. To ensure maximum safety and reduce the risk of harm, tire textiles are made using high-quality PET grades that have significantly high IV values, typically falling within the range of 0.95–1.05 dL·g^−1^ [133]. The exceptional quality and significant quantity of utilized textile fibers necessitate distinct approaches for reusing them, thereby underscoring the significance of tire recycling.

The first step in recycling the aforementioned textiles involves separating the various components of the tires, including rubber compounds, steel wires, and textile fibers. Cryogenic and ambient grinding [243], water jet pulverization [244], and supercritical solvent pulverization [245] are among the methods currently proposed for powderizing tires while separating the steel and textile components. Methods such as water jet and supercritical carbon dioxide (CO_2_) pulverization are particularly advantageous because they are environmentally friendly and partially devulcanize the tires during the process. Once separated from the tire and sorted, the fibers can be recycled using standard methods. However, the presence of impurities, such as tire particles, can hinder the recycling process. Supplementary procedures are thus required to remove rubber particles before recycling [246].

**Table 2:** Overview of ATS using different IR methods; accuracy based on selected test cases in the main references.

IR Method	Algorithm	Fiber	Accuracy	Challenges	Advantages	Ref.
VNIR	Quadratic discriminant classifier, SVM, PCA	Cotton/viscose	68%	A more extensive sample set is needed to guarantee a robust sorting system for all textile varieties.	Compared to the NIR range, the VNIR range offers higher spatial resolution, cheaper and compact cameras, and blue denim sorting.	[236]
		PET	70%			
		Polyamide	100%			
		Wool	100%			
		Cotton and PET	Misclassified 50% as cotton and 40% as PET			
		PET and cotton	Misclassified as cotton			
ATR-FTIR	PCA, CVA, k-NN	Polyamide, PET, viscose, cotton, linen, wool, silk	100%	For real sorting machinery, implementing the specific software to establish a robust IR-spectra database for correctly classifying dirty-wet textile waste.	Automatically classified fiber samples with 100% accuracy and high speed, without any involvement of prior analytical treatment of the textile samples.	[24]
				Post-sorting, a second sorting by color is required to reduce additional dyeing.		
				Strict maintenance protocol must be ensured for the contact between the sensor and the textile to register and compare the IR spectrum with the database for classification.		
NIR	SVM, MLP, CNN	Pure PET slash, pure PET normal, pure wool, pure cotton normal, PET/polyamide, PET/wool, PET/cotton slash, PET/cotton, polyamide	92–98%	To ensure higher prediction accuracy, a more comprehensive sample spectrum information is required to be included in the established standard spectral library.	The proposed method is simple and practical, presents a fast identification speed and a higher recognition rate, and can be applied to a wide range of applications.	[239]
NIR	SIMCA	Cotton	93%	Low accuracy arises for cotton and polyester due to their relatively close spectral characteristics.	This is a nondestructive, simple, and fast method that can identify fibers with a total recognition rate higher than 95%.	[226]
		PET	92%			
		PA6,6, acrylic, wool, silk	100%			
NIR	PCA, SIMCA, LDA	Cotton, Tencel^TM^, PP, PLA, PET, wool, cashmere	100%	As there are many overlapping features in the chemical and physical analyses of wool and cashmere, there is lower accuracy for both textiles.	Using the mentioned algorithm for this method, seven textile fiber types were identified quickly and accurately.	[230]
ATR-FTIR	PCA	Wool, silk, cotton, linen, viscose, PET, PA6,6, acetate, Tencel^TM^, acrylic, elastane, 15 two-component textiles	n.d. ^1^	Mixed textiles are highly inhomogeneous. Thus, in such cases, homogeneity is tested using microscopic or IR-microspectroscopic analysis.	ATR-FTIR spectroscopy enables quick, easy, and nondestructive classification and semi-quantitative analysis of textiles.	[235]
NIR	PLS, ELM	Wool, PET, PAN, PA6,6	Not mentioned	For the analysis, the number of spectral variables was reduced. However, the key to the successful utilization of an NIR-based analytical method is to construct a robust calibration model using samples with sufficient representativeness.	The ELM method is superior to the conventional PLS.	[238]
					The developed procedure may have commercial and regulatory potential to avoid laborious, time-consuming, and expensive wet chemical analysis.	
ATR-FTIR, r-FTIR, mATR-FTIR	Principal component-based discriminant analysis and random forest-based machine learning	Wool, silk, cotton, linen, jute, sisal, viscose, acetate, Tencel^TM^, fiberglass, PET, PA6,6, acrylic, elastane, PE, PP	99% (r-FTIR)	Obtaining a good-quality r-FTIR spectrum of elastane was problematic as the reflectance spectra of the used elastane thread was distorted.	r-FTIR is a suitable technique for the quick, easy, nondestructive, and non-invasive analysis of different types of textile samples. For analyzing very small threads (up to 10 individual fibers), a possible mATR-FTIR approach should be preferred.	[234]
			96% (mATR-FTIR)	For extraneous materials on sample fibers (additives or contaminants), the spectrum recorded is influenced by contaminants.		
FTIR	PCA, SIMCA	Viscose, PA6,6, acrylic, PET	97%	The developed data-mining models can be made more extensive by adding more data of different fiber types, which will result in a sophisticated forensic tool for fiber discrimination.	The combination of spectroscopy and chemometrics has led to a highly desirable method for clustering and classifying 138 synthetic fiber samples into four groups.	[248]

^1^ Not determined.

### 3.2. Reuse

Through collection and manual sorting, end-of-life textile products with sufficiently high quality can be identified as fit for reuse. This quality requirement leads to the absence of technical textiles in the reuse industry because these textiles are developed for their functions rather than their aesthetics. This means that they are generally used until their functions fail, after which they are no longer suitable for reuse [249]. The opposite is true for clothing, which is often discarded before the mechanical properties fail for fashion reasons. As such, much of the literature on textile reuse is centered on secondhand clothing [250]. Household textiles are situated between these two extremities and are generally reused in some capacity, but not as much as clothing is [251].

In the context of ISO 5157:2023, “reuse” appears to be an umbrella term for three separate end-of-life processes—reuse, repair, and remanufacturing—as noted in other definitions in the same standard [186]. All these processes involve at least minor treatments to the product, but crucially, the product is maintained throughout the process rather than being dismantled into secondary raw materials that may be used to create other products.

Actual reuse—sometimes called secondary use—involves the least amount of treatment and is usually limited to washing and sometimes refinishing or refreshing of color [186,252]. This happens more on an informal level, through the exchange or donation of clothing or household textiles within families or between friend groups, than at an industrial level [253]. The more formal market is established by charity organizations and secondhand shops, internet platforms, and textile libraries, where secondhand textiles can be bought or exchanged [254].

In Europe, 22% of clothing waste is collected for reuse or recycling, of which 7–10% is suitable for reuse but only 1–3% consists of clothing items that are fully functional and have a market value by virtue of their being fashionable, hence entering the market for actual reuse [189,250]. In recent years, these numbers have been increasing due to the Extended Producer Responsibility for Textiles Decree, which states that from 2025 onward, producers will be responsible for the preparation of textiles for reuse or recycling at the end of their lives [255].

Repair denotes a process in which waste textile products are not fully functional but still do not have major defects, and there is a simple replacement of damaged parts [186]. For most clothing or household textiles, this involves repairing small details, such as patches of fabric, buttons, or zippers, although items with integrated or hidden electronics can be a hindrance to this process [256]. After repair, the products are entered into the secondhand clothing or household textile market, as if they were fully reused.

Remanufacture can occur if more major (industrial) procedures are required to return a product to its original condition [186]. While there is no universally accepted definition of remanufacturing [257], Lund presented a general working definition as early as 1984: “Through a series of industrial processes in a factory environment, a discarded product is completely disassembled. Useable parts are cleaned, refurbished, and put into inventory. Then the product is reassembled from the old parts (and, where necessary, new parts) to produce a unit fully equivalent and sometimes superior in performance and expected lifetime to the original new product” [258]. This has been used for decennia within the engineering and electrical and electronics industries but is a new concept for the textile industry [259]. While it is necessary to reassemble the product after disassembly, the reassembled product does not need to have the same identity as the original (waste) product. Clothing items can be transformed from one piece into another (e.g., a pair of jeans into a jacket), depending on fashion trends, for example [259].

Reuse is higher up on the waste hierarchy than recycling because it has inherent advantages. It does not require as much sorting, packaging, transportation, manufacturing, nor a whole recycling process, and thus results in a larger net decrease in a product’s environmental and economic impact [260]. For example, clothing libraries were found to be environmentally beneficial if a garment’s service life was considerably extended [195]. An extension of 3 months leads to an average reduction in its carbon and water footprints of 5–10% [261,262].

While more advantageous than recycling in the aforementioned aspect, it should be noted that an extension equal to its original service life, wherein a new owner uses the product as many times as the virgin product—i.e., a 1:1 replacement rate—is unrealistic [189]. This is mostly because textiles are damaged during the use phase of their lifetimes, and this damage is not repaired during reuse processing. For textiles that are washed during their use phase, most of the damage is incurred due to the mechanical stress that fibers are placed under during laundering [227]. Aging is also an important factor, for both clothing and household textiles. Physical or mechanical aging is caused by bending, stretching, and abrasion of the fibers [263], which cause an ordering of amorphous polymer chains and thus increases crystallinity, which makes the fibers stiffer and more brittle, with a tendency to deform plastically or break under stress due to a decrease in the molecular mobility of the polymer [264]. Chemical aging, on the other hand, is caused by the presence of aqueous solutions, oxygen, or high temperatures [263], which can cause changes in the molecular bonds in the polymer chains (e.g., through hydrolysis, oxidation, and thermal scission). Scissions in polymer chains can cause a rapid decrease in the degree of polymerization and, thus, average molar mass. In turn, this decreases the tensile strength and elasticity of the fibers and can also introduce more reactive sites to the polymer chains, thus affecting their chemical reactivity [265]. This decrease in fiber quality means that products can be reused only for a certain amount of time and should be recycled when they are no longer of sufficient quality.

Besides the technical limitations, reuse is also highly dependent on local legislation, which may allow or prohibit the reuse of certain items (e.g., the reuse of baby clothing is sometimes prohibited due to the possibility of it transmitting diseases) [260], and on the education of consumers, who require a level of awareness for secondhand shopping to be developed profitably [266].

### 3.3. Pretreatment Prior to Recycling

Textile waste that is not fit for reuse often requires some form of treatment before it can be recycled, incinerated, or landfilled. This is especially true for waste that is destined to be recycled, as the quality of the outputs of recycling processes is highly dependent on the quality of their inputs, and virtually all recycling technologies are limited to textiles with specific compositions. This is why sorting is important (see Section 3.1.2), but the sorting process takes only the chemical composition of the bulk of the waste into account. In reality, textile waste often comes with so-called hard points (e.g., zippers, buttons, or labels), is colored with dyes or pigments, is finished or coated with specific chemicals, or may have been contaminated during its use phase [267,268]. Certain contaminants can simply be washed away, but certain additives are much more difficult to remove, yet they must be removed for optimal recycling. In addition, not all recycling technologies can accept waste of any shape or structure, so size reduction pretreatment is often necessary. This size reduction is often the first step of any pretreatment and can even take place before the sorting phase for waste that cannot be reused as a product (e.g., pre-consumer waste). In practice, it usually consists of simple mechanical cutting and shredding operations [269]. Waste textile materials can even be ground into powder to increase their accessible surface areas and decrease their crystallinity, but this destroys any fiber structure and is thus not applicable to mechanical fabric, yarn, or fiber recycling. Additionally, it limits the separation options for blended fibers and should thus be used only for single-polymer textile waste [270]. Some mechanical fiber blend separation techniques using centrifuges, cyclones, sink-float baths, or electrostatic separators do exist, but these increase the cost of the process and are highly limited by the degree of entanglement of the different fiber types [271].

On the other hand, the forms of textiles, whether in the form of yarns or fabrics, significantly impact the challenges faced during recycling. Yarns, especially those made from blended fibers, can become tangled or knotted in machinery, leading to clogging and inefficiencies. Their fine, flexible structures make mechanical recycling difficult, often resulting in jams and equipment wear. Fabrics, particularly those woven or knitted from blends such as polycotton or elastane with polyester, present additional hurdles due to their tightly interlaced structures, which resist separation and can deform during processing, causing inconsistent recycling results. Blended textiles, with their varying fiber properties, complicate both mechanical and chemical recycling because differences in melting points or chemical behaviors often lead to contamination and reduced quality of recycled materials. These challenges necessitate the use of specialized recycling techniques to effectively manage and process diverse textile forms [272,273].

Disassembly (or trim removal) is the name given to the pretreatment steps that involve the removal of hard points from textile waste. These include elements not made of textile materials, such as zippers, buttons, and rivets, but also elements made of textile materials with compositions different from those of the bulk textile, such as labels, patches, and linings [274]. These foreign elements can be removed from textile waste by hand, which provides high removal accuracy and minimizes the lost textile surface area, but this is very labor-intensive and thus expensive. For this reason, automated textile disassembly (ATD) techniques are being developed [269].

The oldest and only commercially prevalent ATD technique is built into the mechanical fiber recycling process, rather than being a separate pretreatment step. In this case, textile waste that has been cut down to size is subjected to pickers that open the textiles and tear off the hard points before being garneted fully. During these picking or garneting phases, other mechanical separation systems can also be implemented, such as cyclone separators, sink-float baths, or magnetic separators. The major downside of this built-in system is that it does not remove all types of hard points, focusing mainly on the non-textile elements but ignoring the labels, patches, linings, etc. Machinery manufacturers ANDRITZ Laroche and Dell’Orco & Villani offer tearing lines with this built-in ATD technique [268].

The second type of ATD system is a more recent newcomer to the market. It requires that the waste be cut up into square pieces (clippings) by two successive cutting machines, after which the squares are separated into squares with hard points and squares without hard points, either by visual means (camera identification) or mechanical means (based on physical characteristics, such as density or shape). In this system, the size of the clippings plays a major role because smaller clippings lead to less material loss when a square is expelled, but they also place a limit on the fiber length, which is detrimental for mechanical fabric/yarn/fiber recycling [194,269]. An example of this ATD system is Trimclean, developed by Valvan, which utilizes cameras that feed images into neural network software. This software decides which squares should be separated from the bulk through pneumatic ejection. It also uses metal sensors to detect non-textile trims more accurately [275].

The ATD technology that is most difficult to implement is automated customized cutting, where the waste does not need to be cut into clippings; instead, a robot cuts hard points from the waste item on an article-by-article basis, either with a blade or a laser. The hard points are then separated from the rest of the material by the robot itself or by any of the aforementioned mechanical separation techniques. This process requires accurate cameras and algorithms to recognize the type of waste and its hard points. It also requires that the waste articles be laid out properly before being scanned. Despite these challenges and their associated costs, the advantages are that minimal recyclable fabric is wasted and the fiber length of that material is kept at its maximum. This system is still being researched by companies such as CETIA and is not yet commercially available [269,276].

Besides hard points, colorants (in the form of dyes or pigments) are often present in textile waste. These rarely interfere with recycling processes, although they have been reported to coagulate and form insoluble particles in re-extrusion or chemical recycling processes, which gives rise to problems during spinning [277]. If colorants are not removed during a pretreatment step, they will also be present in the secondary raw material, limiting the market for the material and lowering its value.

Theoretically, there are two options for the removal of color from a material: removal of the colorant molecules themselves or destruction of their chromophore groups. The latter renders them inert but leaves them in the material. As the removal of colorants from textile waste is not a crucial step in the overall recycling process, there has not been much research on it. It is likely, however, that for the colorant extraction method, principles similar to those applied to the removal of colorants from textile wastewater after the dyeing process (e.g., adsorption, coagulation, flocculation, and solvent extraction) can be used if the colorants can first be extracted from the solid waste and placed in a liquid environment [7,8,278,279,280,281,282,283].

For example, Yousef et al. noted that treating denim waste with nitric acid (HNO_3_; concentration below 60%) can leach dyes from the fabrics, after which the spent acid can be regenerated with activated carbon (AC) [284]. The chromophore destruction method was investigated by Powar, who found that textiles colored with reactive dyes (mostly cellulose-based textiles, such as cotton) can undergo a conventional alkaline reductive treatment using NaOH and a reducing agent (e.g., sodium hydrosulfite) for color stripping. Alternatively, they can be treated with ozone for up to 98% color removal, though with serious mechanical damage incurred to the fibers [285,286].

Glucose as a green reducing agent was proposed as a third option and was found to remove color with much less incurred damage—even less damage than with the conventional alkaline treatment—but with the requirement of a higher processing temperature. Powar also noted that this was only possible for dyed textiles because pigments were extremely difficult to destroy, even with ozone [287]. Määttänen et al. also found alkaline washing and ozone treatments to work for the destruction of dyes from cotton materials alongside treatment with hydrogen peroxide [288]. This color removal is made very challenging specifically in post-consumer waste because information on which dyes or pigments were used in a product or their remaining contents at the end of their lives is often lacking [289].

Other major additives present in textile waste are the chemicals used in the finishing or coating of the fibers. These chemicals are applied to give the product functional properties, such as anti-microbial properties, anti-wrinkle properties, softness to the touch, and flame retardancy, but they often remain in significant quantities in textile products at the end of their lives [290,291]. Some of these chemicals can be ignored for recycling purposes, thus remaining in any secondary raw material. Other chemicals must be removed during the pretreatment step, or they will interfere with the recycling process itself and lead to technical problems, such as low solubility and decreased dyability [267,292]. For example, Björquist [293] found that cotton treated with dimethylol dihydroxyethyleneurea (DMDHEU)—an “easy-care” anti-wrinkle finish—was difficult to dissolve into NMMO during the production of lyocell fibers, even though that is their conventional solvent Similarly, Egan et al. [270] discovered that the presence of DMDHEU on cotton presented a large obstacle to enzymatic biodegradation.

The method required for the removal of the aforementioned chemicals depends entirely on the chemical structure of the finishing chemical and its method of application onto the textile substrate. In general, chemical degradation and subsequent dissolution of the finishes is the most common, as is the case for the aforementioned DMDHEU examples, where the finishes were removed via acidic and alkaline hydrolysis treatments [294,295]. The CreaSolv process by CreaCycle GmbH is an example of a treatment used to remove thicker coatings or layers of textiles adhered to each other with an adhesive. For example, it could delaminate or dissolve PU, polyamide, PVC, and acrylate coatings from PET and polyamide substrates [194].

An alternative to dissolution for coatings is the use of an adhesive polymer material between the coatings and the substrate, which can be activated with a certain trigger. An example of this is the INDAR system by Rescoll Technological Center, which includes blowing agents in its adhesive activated at a certain temperature (130 °C, 150 °C, or 170 °C), thus causing easy debonding at that temperature. This is conceptually similar to the ADT system using disintegrable stitching threads, and it has the same downside of requiring this adhesive upon conception of the product. It has been proven to work for the debonding of PU coatings from PET textile substrates [296,297].

A third option is the PolySep process, in which shredded, coated textile waste is soaked in a solvent and then brought into contact with hot water or steam, leading to flash evaporation of the solvent and detachment of the coating from the substrate. This was originally developed for the removal of PVC layers from PET substrates [298].

Contrary to the aforementioned examples, some chemical processes are adapted to finishing chemicals and can non-selectively degrade them while degrading the polymer, making a separate finish removal pretreatment unnecessary [267]. It should be noted that removal of finishing chemicals can usually occur before or after the size reduction pretreatment step [299].

While methods can be found to remove all the aforementioned additives, more focus should be placed on the application of these additives during the design of the textile product, also known as design for recycling. Options such as the use of destructible stitching threads and triggerable adhesives that cause delamination should be considered more, and legislation should take into account which colorants and finishing chemicals are currently removable to enforce their use or restrict the use of others.

In addition, to treat wastewater from textile recycling, advanced composite adsorbents are utilized for the efficient removal of contaminants. These adsorbents, such as those incorporating mesoporous silica combined with specific organic ligands, are designed to selectively capture and separate targeted metal ions, such as ytterbium (Yb(III)) and samarium (Sm(III)). The composite adsorbents, with high surface areas and specific bonding capabilities, operate effectively under controlled pH conditions to avoid issues such as hydroxide precipitation. For instance, the materials exhibit high selectivity and adsorption capacities, with Yb(III) and Sm(III) ions being successfully removed even in the presence of other competing ions. Additionally, these adsorbents can be regenerated and reused multiple times, maintaining their efficiency throughout numerous cycles of adsorption and elution. This method not only improves the quality of treated water by removing hazardous metal ions but also offers a sustainable solution for recycling and resource recovery in textile processing [280,281,282,283,300].

### 3.4. Mechanical Recycling

When reuse is no longer possible for a waste textile product, the next-best end-of-life option is mechanical recycling. Contrary to reuse, mechanical (and chemical) recycling does not attempt to maintain the waste product but instead breaks it down into materials which can then be used in manufacturing processes to create new products, whether the same as the original (primary or closed-loop recycling) or different from the original (secondary or open-loop recycling) [189,191,192].

According to ISO 472:2013, mechanical recycling specifically includes all recycling processes that do not cause a significant change in the chemical structure of the polymers that make up the material [188]. This includes breaking down the waste textile product into its fabric, yarn, fiber, or polymer building blocks and allowing a classification embedding physical recycling. In the following sections, the main mechanical recycling methods are addressed.

#### 3.4.1. Fabric, Yarn, and Fiber Recycling

Textile products can be broken down into fabrics, yarns, or fibers through purely mechanical processes, such as cutting, pulling, and blending [301,302]. While fabric and yarn recycling are possible in theory, there is almost no literature on these processes, and the few sources that do speak of fabric recycling conflate it with remanufacturing, which is similar in practice but maintains the product as a whole, rather than viewing the fabric as a secondary raw material output of the end-of-life process.

In contrast, fiber recycling is a well-developed technology and is currently by far the most used method in industry for the recycling of textile waste, more prevalent than polymer recycling or any form of chemical recycling [303]. The objective of this technique is to remove any impurities in textile waste and restore the fibers to their original state so that they can be reused in the production of new textiles [304]. The main process of this restoration consists of the tearing and opening or garneting of textiles into loose fibers by a so-called Garnett machine [305,306], after which the fibers can be respun into yarns or processed into nonwovens.

Fiber recycling uses almost no chemicals, very little water, and less energy than other recycling methods, largely because it requires the fewest processing steps to achieve a new product [60,267]. While this makes fiber recycling the cheapest recycling process, it also has downsides. For example, the heavy mechanical strain that the fibers experience during the recycling process causes shortening of the fibers, and a non-recycled fraction of dust is left over by the process. This reduction in the length of the fiber can pose limitations to its processability into yarn and means that the fibers have reduced mechanical properties, such as tensile strength [307]. This is the reason why up to 95% of mechanically recycled fibers are currently used as flocks in nonwovens or as additives in other products (e.g., bricks), rather than being respun into lower-quality yarns [308,309]. In other words, due to the damage sustained by the fibers during mechanical recycling, this method can be considered open-loop downcycling. Ensuring consistent quality in the recycled fibers produced can thus be challenging [310].

The only way to achieve open-loop side cycling or closed-loop recycling through mechanical means is by adding longer virgin fibers to the shortened fibers upon spinning them into new yarns. Notably, this often still requires at least 50% virgin fibers [311] because only this can allow the new yarns to achieve the required mechanical properties. However, studies have pointed out that lubricants (e.g., a polyethylene glycol 4000 aqueous solution) can be applied to fabrics before shredding to reduce inter-fiber cohesion and friction and thus reduce the damage caused by the garneting process [311]. In general, this true closed-loop recycling is attempted only with pre-consumer textile waste that is clean, sorted, and of high purity [312,313]. Despite this reduction in fiber quality, a good approach to textile recycling would be to allow mechanical fiber recycling until the fiber length has been reduced to a level at which the material is no longer fit for reuse or for fabric, yarn, or fiber recycling, and to then cascade into polymer recycling or chemical oligomer/monomer recycling [189].

While fiber recycling can usually process textile waste with additives, such as colorants and finishing chemicals, without their removal in the pretreatment step, these additives are left in the recycled product. This limits the purpose for which the secondary raw material can be used because it continues to contain the same finishing chemicals, which can affect the mechanical properties of the recycled fibers [314]. In addition, mixes of unsorted colors often come out as black or gray [309]. These factors can reduce the value of the secondary raw material and lead to conflicts with the EU regulation on the registration, evaluation, authorization and restriction of chemicals (REACH) [267,314].

Similarly, fiber recycling has no easy solution for fiber blends, such as cotton–polyester, viscose–polyester, or cotton–elastane. Processing these fibers together generally results in a lower-quality fiber of mixed colors and fiber length [312]. Even pre-consumer blends can be problematic. This is especially true for any fiber blended with elastane because the characteristics of the highly elastic elastane fibers are very different from those of other fibers. As such, most firms send textiles containing elastane straight to landfills rather than attempting to recycle them [315]. The combination of these factors indicates that a rigorous pretreatment is required for most textile waste prior to fiber recycling (cf. Section 3.3).

In the current industrial landscape, fiber recycling is applied mostly to fibers of natural polymers, such as cotton, animal fibers (wool, cashmere, etc.), and man-made cellulose fibers (viscose, lyocell, viscose, cupro, bamboo viscose, etc.), but it is also applied to polyester products and waste with combinations of natural polymer fibers and polyester fibers [316]. Although significant investments in technology and infrastructure are still necessary to overcome the challenges posed by fiber recycling and to fully achieve its benefits [317,318,319], there are already some very successful fiber recycling plants.

At Hilaturas Ferre in Spain, for example, pre-consumer cotton waste (and, optionally, a small amount of post-consumer waste) is recycled into staple fibers of 10–15 mm in length, which can be blended with acrylic or (recycled) polyester fibers to produce new yarns with 90% recycled content [320]. They also have plants for recycling polyester and wool from post-industrial sources [289]. Similarly, Pure Waste in Finland recycles pre-consumer cotton waste via shredding, and the company claims to be able to produce 100% recycled yarns, which can be mixed with recycled polyester or viscose yarns for the production of clothing [321].

#### 3.4.2. Polymer Recycling via Melting

After fiber recycling, the next method in the waste treatment hierarchy is polymer recycling (see Figure 9) [195]. This entails the breaking down of fibers into their constituent polymers without further degrading the polymers in any pronounced way, after which they can again be extruded into fibers. As such, polymer recycling is often called re-extrusion. This is still considered mechanical recycling because the chemical structure of the polymer remains intact [188]. Because polymers can be used as the input material for the same product or an entirely different product, polymer recycling can be both closed-loop (primary) and open-loop (secondary) recycling. In the latter case, an effort is usually made at upcycling or side cycling, but downcycling is also possible.

The most straightforward way for fibers to be broken down into polymers is by melting them and applying mechanical forces to them. This is specifically referred to as thermomechanical recycling in ISO 5157:2023 [186]. It is a widely used method of mechanical recycling, particularly for fibers of synthetic polymers [322,323], more specifically polycondensation polymers such as PET and polyamide [324,325,326].

**Figure 13:**
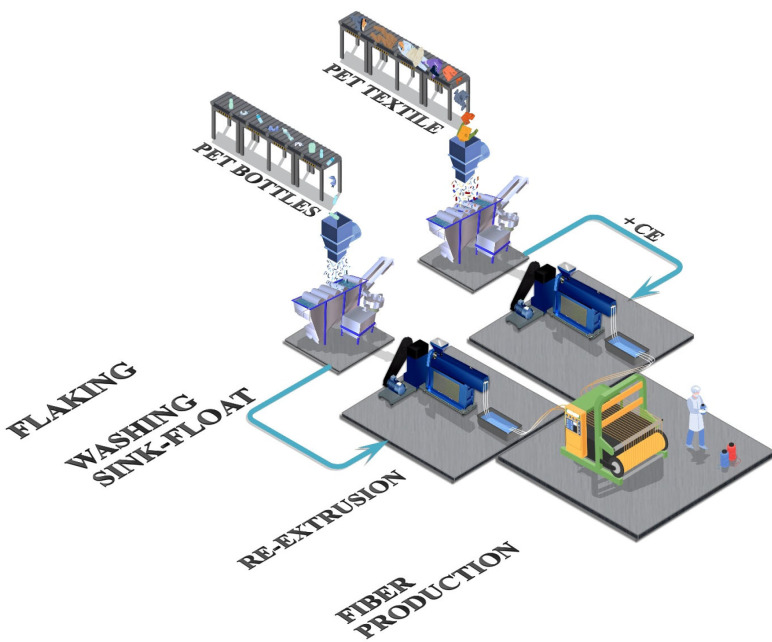
Re-extrusion of fibers from PET bottle or textile waste.

The general process is very similar to the virgin extrusion of thermoplastic polymers, with differing input materials. In this case, pretreated waste fibers are usually cut, compressed into small balls or pills, dried, and then fed into a screw extruder, which melts and immediately extrudes the polymer (cf. Man-Made Fibers of Synthetic Polymers Section) [327]. While compression makes the feeding easier, it also requires an extra step, labor, and energy, and is thus sometimes ignored [328]. For less compressed material, using a twin-screw extruder can be beneficial [327]. The process is shown in Figure 13. Note that nonfibrous waste can also be recycled into fibers via this method, and that this method is in fact more common than fiber-to-fiber polymer recycling in the current industry, particularly for PET bottle waste [328].

A major benefit of the thermomechanical polymer recycling process is that it is much simpler, cheaper and requires fewer chemicals than any chemical recycling process. In addition, the economic value of recycled fibers is generally higher than that of fibers originating from mechanical fiber (instead of polymer) recycling methods. This is because the re-extruded fibers do not suffer from the mechanical damage or fiber shortening associated with the mechanical shredding of fabrics. With suitable care, the resulting fibers can rival the quality of virgin fibers [329].

In practice, however, several degradation reactions can occur during re-extrusion. Excess friction inside an extruder can lead to mechanical degradation of the polymer, reaction with oxygen at high temperatures can lead to thermo-oxidation of the polymer, and any water in the input material can lead to hydrolysis of the polymer. To address these problems, respectively, special screw designs and force feeders can be used, the re-extrusion can be performed in an inert nitrogen (N_2_) gas atmosphere, and the material can be dried more thoroughly to a very low level of residual moisture (below 0.1 g of water per kg of fibers) [327,328,330].

Aside from the aforementioned risks of any re-extrusion process, certain waste fibers can also contain contaminations, such as other polymers (e.g., PVC, even at levels as low as 100 ppm, with hydrochloric acid (HCl) being released), carbon particles, pigments, dyestuffs, Sb_2_O_3_ catalyst residue, stabilization agents, and flame retardants. These contaminations can all cause re-extruded fibers to turn yellow or gray [331] and thus pose challenges for the production of recycled textiles with white or pastel shades [332].

Some of the aforementioned damage will be unavoidable and lead to a decrease in the viscosity of the melt and the average molar mass of the polymers within it. As a result, it can be necessary to increase (or prevent a further decrease in) the average molar mass before or during re-extrusion, either through the addition of stabilizers or certain chemical processes, until it reaches the correct average molar mass range suitable for fibers [157]. For instance, the SSP or LSP process can be utilized to increase the average molar mass of PET and polyamide, although the high operational and equipment costs of both processes can be significant obstacles, particularly their high energy costs [333].

A more common process is reactive extrusion (REX), whereby other molecules known as chain extenders (CEs) are added to the waste polymer [334,335,336]. Generally, CEs are bifunctional or multifunctional compounds that can link with the terminal functional groups of polymers. CEs can be either low-molar-mass compounds or macromolecules. Linear CEs are useful for fiber grades because they can enhance the chain alignment and crystallinity of the drawn fibers [337].

**Figure 14:**
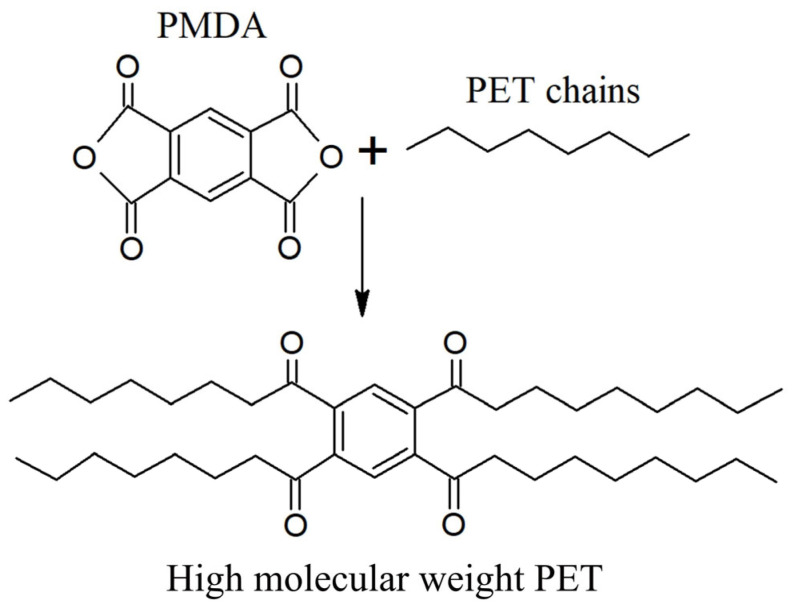
The chain extension of recycled PET using PMDA [338].

Figure 14 shows an example of a chain extension of recycled PET using pyromellitic dianhydride (PMDA), a common CE [338]. The primary suitable CEs for polyamide include biscaprolactam [339], bisoxazolines [340], and a combination of the two [339]. PMDA [338], bisoxazolinones [326], different phosphides [326], bisoxazines [339], bisoxazolines [341], epoxidic multifunctional oligomer [342], bisepoxides [339], and isocyanate [343] are some suitable CEs for PET. In some cases, the use of CEs can lead to the formation of by-products, foreign building blocks, and a broader molar mass distribution or molecular weight distribution (MWD), which can affect plastic properties, causing more shrinkage. Therefore, under REX conditions, vacuum evaporation should be employed to remove by-products [344].

Furthermore, Lee et al. [345] applied aminopropyl-functionalized polyhedral oligomeric silsesquioxanes as suitable CEs. By using this nanoparticle-nucleating agent, the crystallization rate and temperature of PET could be increased 2.7 and 1.2 times, respectively. Similarly, Makkam and Harnnarongchai [346] used an acrylic epoxy resin as a CE to increase the average molar mass of PET. The results indicated that the CE can be accompanied by chain branching or even cross-linking. They found that the MWD and gel content of modified PET increased when the concentration of acrylic epoxy resin was increased. However, the melt re-extrusion recycling process has a major limitation: It can be directly used only for pure thermoplastic fibers (e.g., PET, polyamide, and PP). Fiber blends cannot be easily processed if the flow properties of their melts and/or their melting temperatures are too different (they can preferably differ by, at most, 10–20 °C). Thus, extensive fiber separation during pretreatment is necessary. Any remaining finishing or coating products on the fibers will also cause degradation during re-extrusion. Thus, under this recycling method’s conventional conditions, it is not an option for natural polymers, thermoset synthetic polymers, more complex textile blends, or coated fabrics [327].

#### 3.4.3. Polymer Recycling via Dissolution

As discussed in Section 3.4.2, the main goal of any polymer recycling process for textile waste is the recovery of the polymers that make up the fibers in a liquid form so that they can be re-extruded into new fibers. As an alternative to melting, this liquefaction can be achieved by dissolving the waste fibers into a suitable solvent. This method of recycling is applicable for polymers that cannot melt—because, for example, they would degrade before reaching their melting temperature—while being soluble in a suitable solvent. It excludes many thermoset polymers, whose cross-linked network structure prevents dissolution [269]. As such, this dissolution still covers a wide span of fiber types, but in practice, this recycling method is currently applied at a commercial scale only to cotton and sometimes polyester waste [347,348,349]. Several pilot plants or small-scale operations do exist for other textile types, such as acrylic textiles (Thai Acrylic Fibre Co., Regel in Bangkok, Thailand [350]), PP and PE textiles (Obbotec-SPEX in Rotterdam, The Netherlands [351]), PP textiles (PureCycle Technologies in Orlando, FL, USA [352]), and aramid textiles (Teijin Aramid in Arnhem, The Netherlands [353]), each utilizing different dissolution reactors and parameters, which the companies do not disclose. In general, however, dissolution happens in approximately 30 min (after pretreatment) and is conducted below the solvent’s boiling temperature [354].

**Figure 15:**
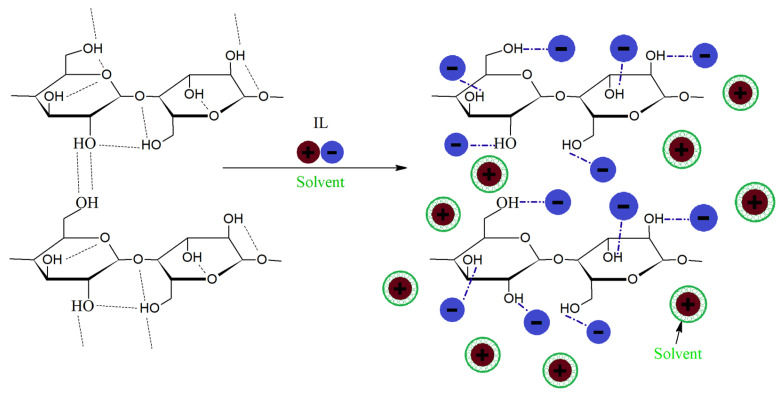
Dissolution of cellulose in an ionic liquid using the anti-H_2_ bonding mechanism [357].

Cotton consists of cellulose, which can be suspended in a liquid and then re-extruded into man-made cellulose fibers by means of wet spinning [267]. Cellulose has low solubility in most chemical solvents due to the strong H_2_ bonds between its molecules; thus, it is necessary to first weaken or disrupt the H_2_ bonding to increase the dissolution rate (an example is shown in Figure 15) [355]. This means that specialized solvent systems are required to facilitate the dissolution of cellulose, depending on various factors, such as the dissolution rate and temperature, pretreatment, and the rate of cellulose degradation during dissolution [40,355]. Common solvents include a cuprammonium solution (leads to cuprammonium fibers), NMMO (leads to lyocell), and ionic liquids (ILs; leads to Ioncell-F^®^) [194,356].

Cellulose can also be slightly depolymerized without breaking it down entirely to glucose, or its chemical structure can be modified to make dissolution easier. This leads to mixed chemical–mechanical recycling results, as is the case for the well-known viscose process. This process uses cellulose-xanthate as an intermediate through treatment with carbon disulfide and subsequent dissolution into NaOH (leads to viscose or modal), or through treatment with urea (CH_4_N_2_O), leading to a cellulose-carbamate intermediate which can be dissolved into NaOH (leads to carbamate) [194].

In the literature, various strong acidic, basic, and ionic solvents have been investigated for the polymer recycling of waste cotton textiles, such as phosphoric acid (H_3_PO_4_)/H_2_O, ILs, trifluoroacetic acid (CF_3_CO_2_H), H_2_O/H_2_SO_4_, H_2_O/NaOH/CH_4_N_2_O, CS_2_/NaOH, H_2_O/lithium hydroxide (LiOH)/CH_4_N_2_O, and dimethylacetamide (DMAc)/lithium chloride (LiCl). As shown in Figure 15, cellulose is dissolved using ILs by repelling negatively charged anion–cellulose complexes through electrostatic forces and by condensing the IL cation around these complexes, leading to steric and solvophobic interactions. Some solvents, such as H_2_O/NaOH/CH_4_N_2_O and H_2_O/H_2_SO_4_, have enabled rapid cellulose dissolution at low temperatures, whereas the LiCl/DMAc system can dissolve cellulose without the need for pretreatment. Despite their advantages, all the solvents mentioned above have certain drawbacks. For instance, the NaOH system has limited cellulose solubility and is unable to dissolve cellulose with a high degree of polymerization, while NaOH/CS_2_ is a derivatizing solvent that causes severe pollution. Moreover, ILs and DMAc/LiCl are not yet economically feasible due to their high costs [104,357].

Theoretically, the aforementioned solvent-based polymer recycling processes need not be limited to cotton, and any cellulose-based textile waste can be used as an input (e.g., virgin man-made cellulose fibers or wood fibers), but these sources of cellulose can vary slightly in chemical structure and lead to different viscosities in the dissolved state, which would necessitate adaptations in the recycling process or pretreatment; thus, cotton is the only major input for these processes to date [358].

In recent years, there has been a significant focus on the process of dissolution for the removal of impurities with inorganic or organic origins from polymeric textile waste to allow the separated polymer to be recycled more efficiently. For example, the dissolution recycling of polyolefins is being researched. It has the potential to be highly effective in removing impurities, separating cross-linked chains, and reducing gel content [359,360]. Moreover, dissolution recycling can help eliminate contaminants such as dyes and additives, which enables in situ catalytic pyrolysis of polyolefins and greatly reduces the costs associated with pyrolysis [361,362,363].

Dissolution recycling has the advantage of requiring less pretreatment compared to many other recycling methods. It can accept material input of any fabric structure (woven, knitted, nonwoven, etc.) without cutting it to size, and it is often tolerant of dyed textiles because the colors can be removed or bleached during the recycling process, as long as it is known which dyes were used [358]. Alternatively, the color can be maintained or regenerated (in the absence of color), significantly reducing the cost of any future dyeing processes if the same color is desired [349]. However, dissolution recycling still requires the removal of hard parts (buttons, zippers, etc.), and the process yield is highly dependent on the purity of the input material, so sorting is still very important [358].

The most important advantage of dissolution recycling is that it can deal with fiber blends without making the process too complex. This requires the use of a solvent that selectively dissolves one fiber without dissolving the others. For example, H_2_SO_4_, NaOH/CH_4_N_2_O, and LiCl/DMAc solvent systems are under investigation for the selective dissolution of elastane out of cotton–elastane blends [364]. Similarly, tetrahydrofurfuryl alcohol and ɣ-valerolactone are green solvents that have been found to be capable of dissolving elastane with an efficiency comparable to that of classical solvents (DMF, DMAc), allowing for the selective dissolution and recycling of polyester–elastane and polyamide–elastane blends [40]. The most common blended materials for dissolution recycling, however, contain cotton and polyester, specifically the so-called polycotton blend. In this case, selective dissolution of the cotton fraction is usually applied, while the undissolved PET is filtered, dried, and subsequently recycled on its own or converted into a derivative compound [365]. Processes that selectively dissolve the PET do exist (e.g., using sulfolane or a switchable hydrophilicity solvent), but they have been reported to reduce the quality of the cellulose fibers [349,366,367]. PET is also more easily recycled via the melt re-extrusion process [365]. Two examples of companies that selectively dissolve cotton in this way are Worn Again Technologies in Nottingham, the UK [368] and Textile Change in Vejle, Denmark [369].

Selective dissolution is valid not only for fiber blends but also for materials with significant nonfibrous polymer fractions, such as fiber-reinforced composites or carpets. For example, polyamide carpets can be recycled by selectively dissolving the fibers in sulfuric, formic or hydrochloric acid (H_2_SO_4_, CH_2_O_2_, or HCl) and subsequently precipitating them in water [370].

While dissolution recycling has the advantage of being able to produce pure polymers of virgin-like quality, this requires more water and energy than a process such as fiber recycling, and it necessitates larger scales to ensure its economic viability [194]. Additionally, the polymer quality is virgin-like only to a limited extent. Research has shown that polymers degrade with each subsequent attempt at dissolution [312,371], meaning that the process cannot be continued ad infinitum, thus making room for chemical recycling. This unwanted degradation can be minimized or avoided with good process design, such as in the process employed by Teijin Textiles in Japan, which has developed a method to dissolve PET and create recycled PET fibers with limited loss in quality [372]. More commonly, however, other input materials are added instead of processing 100% textile waste. Cotton dissolution recycling, for example, is usually improved by the addition of at least 50% virgin wood pulp to cotton pulp [373].

Furthermore, Yousef et al. [374] developed a sustainable technique for extracting cotton from textile waste, which can serve as a new material source that guarantees good quality and economic feasibility. The method consists of three key steps: HNO_3_ leaching, dimethyl sulfoxide dissolution, and sodium hypochlorite and HCl bleaching. These steps were employed to eliminate textile dyes, dissolve the polymeric portion, and release the cotton fibers. It was claimed that the demonstration of the technology at the pilot scale not only illustrated its industrial potential to achieve self-sufficiency in raw cotton fiber but also facilitated the transition to a circular economy.

**Figure 16:**
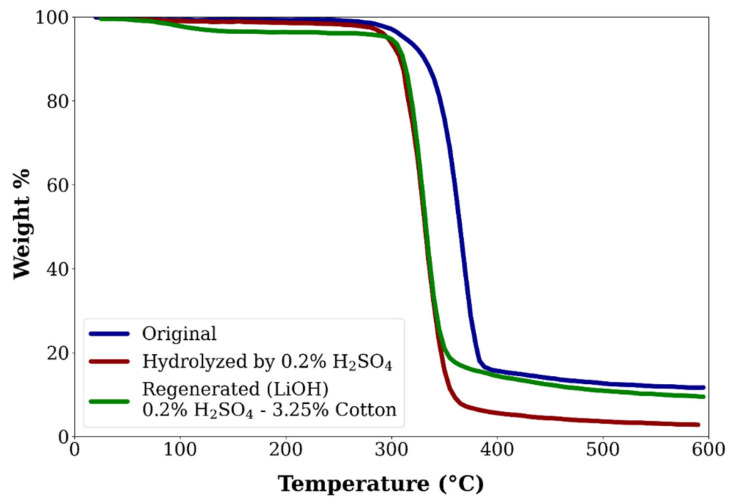
Thermogravimetric analysis curves for original, hydrolyzed, and man-made cotton fibers [348].

Liu et al. [348] applied alkali (LiOH or NaOH)/CH_4_N_2_O aqueous systems as solvents to physically recycle cotton waste. Post-consumer cotton fabrics underwent hydrolysis using H_2_SO_4_ and were dissolved in these solvents to produce man-made fibers via wet spinning. The man-made fibers showed a change in thermal stability, indicating structural modification due to the hydrolysis and dissolving processes. Mechanical testing revealed that the as-spun man-made fibers (without a drawing process) had similar tenacity and competitive elongation to commercially available regular viscose fibers. A low drawing ratio of 1.3 resulted in a 50% increase in fiber tenacity, indicating the potential to produce stronger fibers from cotton waste using industrial wet-spinning facilities and higher drawing ratios. The thermal behaviors of the original, hydrolyzed, and man-made fibers were evaluated using thermogravimetric analysis (TGA). The samples demonstrated a significant weight loss (between 300 °C and 400 °C), corresponding to cellulose thermal decomposition, and the residual char increased at varying levels from hydrolysis to regeneration, yielding about 8–18% at 600 °C (Figure 16).

Yousef et al. [284] also conducted a study in which they employed an integrated and sustainable chemical process to recover pure cotton and PET fibers from textile waste. The process involved the removal of textile dyes from the textile using acid and regenerating the spent acid with AC to restore its original concentration (83%). The researchers then utilized a green dissolution process with a switchable hydrophilicity solvent to dissolve the PET material and liberate high-purity and thermally stable cotton fibers. The solvent’s hydrophilicity was altered to extract PET from the solution, reducing power consumption and PET degradation compared to conventional solvents. The study revealed that cotton fibers and PET constituted 84% and 16% of the fabric, respectively, with a recycling rate exceeding 96%.

Haslinger et al. [375] applied physical recycling to effectively separate cotton from PET. They used 1,5-diazabicyclo [4.3.0] non-5-enium acetate ([DBNH][OAc]) to selectively dissolve the cotton component and then performed dry-jet wet spinning of cellulose to produce textile-grade fibers. The spun fibers were found to possess properties similar to those of lyocell. The PET fraction recovered from the process had a low cellulose content (1.7–2.5 wt%). However, the PET material dispersed in [DBNH][OAc] underwent visible degradation, as evidenced by a decrease in its average molar mass and tensile properties (ca. up to 50%). The results demonstrate that blended textile waste containing cellulose has the potential to serve as a feedstock for conventional fiber spinning processes. In general, physical recycling is one of the feasible approaches for separating natural and man-made fibers from synthetic fibers, such as polyester, in mixed fibers. In this context, Table 3 shows the potential of this approach to release mixed fibers from each other.

### 3.5. Chemical Recycling: Monomer/Oligomer Recycling

Chemical recycling breaks down textiles into their chemical building blocks or oligomers, allowing them to be reused in valuable processes. Commercial polymers, produced through chain-growth or step-growth polymerization, differ in their chemical structures, with the former having hydrocarbon backbones and the latter featuring functional groups within the chain.

**Figure 17:**
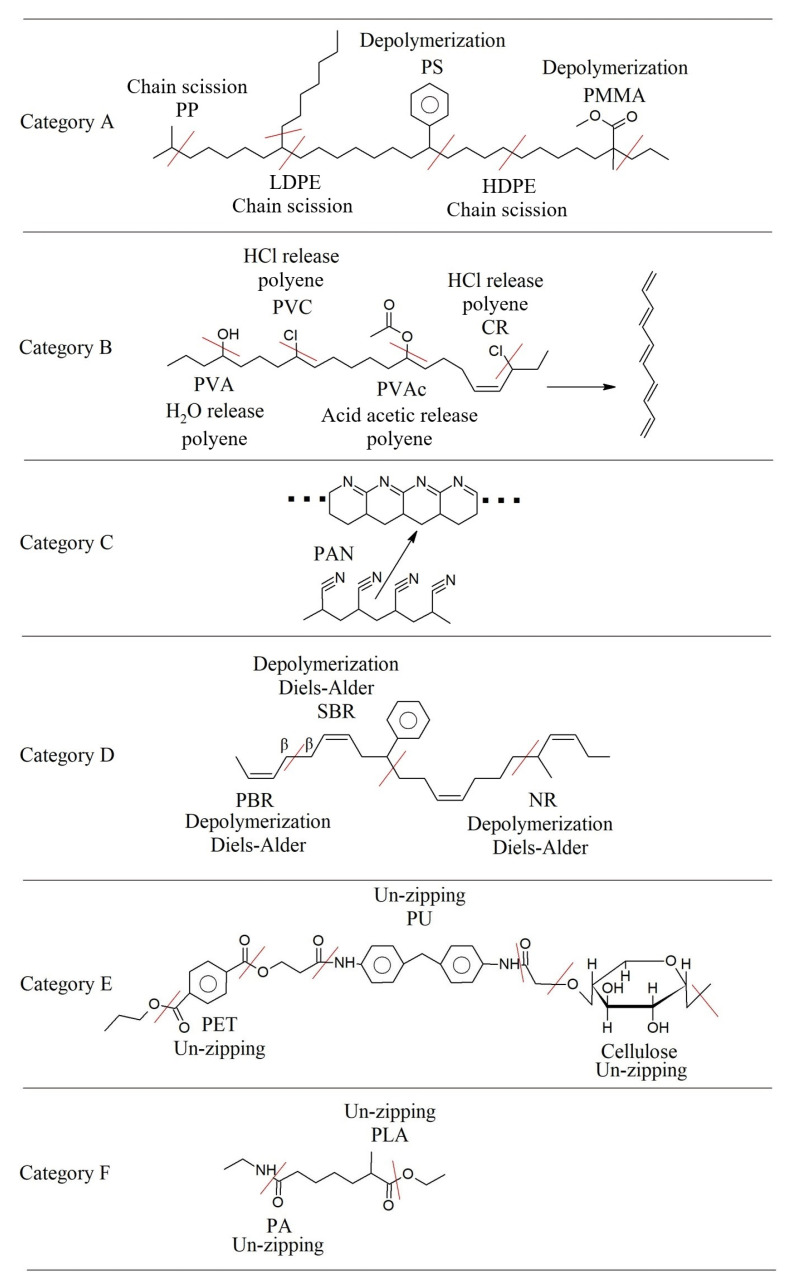
Functional groups of six polymer types, with examples theoretically lumped into one chain for the compactness of the figure [392,393,394,395,396,397,398,399,400,401,402,403,404,405,406,407].

Given the diverse applications of polymers in textiles—such as fibers, adhesives, and labels—this section provides an overview of polymer degradation mechanisms, divided into six categories (A–F) based on chemical composition (Figure 17). Next, key chemical recycling methods like pyrolysis, gasification, solvolysis, and biological recycling techniques are reviewed.

#### 3.5.1. Degradation Mechanisms for Different Polymer Categories

Category A polymers are saturated polymers with a hydrocarbon backbone and side groups connected to the backbone through a carbon bond, as shown in Figure 17 (Category A), highlighting the potential location of the initial scission (also known as fission). In these polymers, the tertiary carbons (if present) determine the preferred scission point of the polymer chains [379,380,381]. Bulky functional groups in polystyrene (PS) and PMMA can guide chain scission toward almost-full depolymerization and monomer recovery [382,383,384,385,386,387]. The thermal decomposition of polyolefins at moderate temperatures results in the creation of waxy pyrolysis oils [388,389,390]. However, increasing the temperature can lead to the production of lighter olefins [361,391]. Under typical circumstances, coke formation during the pyrolysis of this polymer category can be disregarded [392].

**Figure 18:**
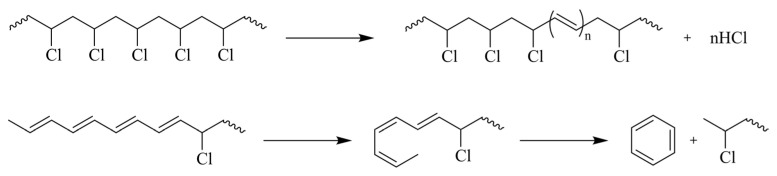
The thermal degradation process of PVC [408].

Category B polymers possess side functional groups connected to the backbone through heterogeneous atoms (Cl, O; Figure 17, Category B). The initial scissions in the degradation of this category occur primarily in the side groups and the formation of a polyene. The chain backbone contains many double bonds, leaving the remaining polyene susceptible to cross-linking reactions and coke formation. The degradation process for this category involves three distinct stages: the release of heterogeneous atoms, cross-linking, and degradation (Figure 17, Category B). Figure 18 shows the mechanism of PVC degradation and the formation of polyene.

Category C polymers are, in essence, devoted to PAN. The degradation behavior of these polymers, which are utilized in the manufacturing of acrylic fibers, strongly differs from that of other polymers (Figure 17, Category C). The degradation of PAN results in the formation of linear or multi-branched polycyclic structures through N_2_ bonding with adjacent carbon atoms, ultimately leading to the formation of stable coke [398]. PAN’s strong propensity for secondary reactions and coke formation makes it a desirable feedstock for producing fibrous AC [409].

Category D polymers are highly unsaturated polymers, such as rubbers (Figure 17, Category D). Regarding their cis structure, most monomers are vulnerable to secondary reactions (e.g., Diels–Alder reactions), resulting in the formation of cyclic compounds ranging from naphthenes to (poly)aromatics. As rubbers are mostly employed in a vulcanized form, devulcanization through fast pyrolysis with a low residence time can lead to the substantial recovery of valuable diene monomers [246,396,399,401].

Note that chloroprene rubber (CR) can be classified as either Category B or D, but its thermal behavior is much closer to that of Category B. The presence of both double bonds and chlorine in the CR structure contributes to a high coke formation rate, with up to 50% produced [407]. This category typically produces a considerable amount of coke during thermal degradation, which can be reduced by increasing the heating rate [395,396,397]. Generally, pyrolysis is not recommended for this category, and alternative methods (e.g., solvolysis, controlled catalytic degradation, or using a suitable carrier gas at high pressures) must be employed to prevent polyene formation.

Nonlinear polymers with heterogeneous atoms in the polymer backbone and potentially with side groups, such as PET, PU, and cellulose (Figure 17, Category E), determine Category E polymers [402,404,405,410]. The associated fibers are highly durable due to the naphthenic and aromatic rings in the chain backbone, as well as H_2_ bonding in some instances. Thus, around 90% of the world’s produced fibers belong to this group [17]. The production of polycondensation polymers through the step-growth polymerization process is reversible, indicating that the bond between monomers is relatively weaker than other bonds. Correspondingly, unzipping is the predominant mechanism during the degradation of this category in cases where the original by-product is a major component. Note that, unlike depolymerization, unzipping during pyrolysis does not result in very high monomer recovery. Due to the high temperature and reaction rate, secondary reactions occur during pyrolysis, which results in a wide range of pyrolysis products (Figure 17, Category E) [411,412,413]. In this regard, a controlled depolymerization technique, such as solvolysis, results in monomer recovery at low to moderate temperatures [414]. In other words, (screw-based) solvolysis can be seen as the most effective chemical recycling method for synthetic polycondensation polymers [415].

Note that while man-made and cellulosic fibers can also be converted into glucose via enzymes or solvolysis, the dissolution and mechanical recycling approach is more practical and desirable [349]. Considering the strong H_2_ bonding in polycondensation polymers (excluding polyesters), it is essential to employ solvents capable of disrupting these interactions during dissolution, as explained above. Specifically, PU functional groups are a combination of ester and amide functional groups. The presence of aromatics in the chain backbone causes secondary reactions to compete closely with chain scission and unzipping during PU degradation. However, the formation of a considerable amount of coke is prevented by bulky urethane groups [416].

Category F polymers differ from the previous category in that their linear structure (Figure 17, Category F) leads to relatively minimal coke formation. However, the appropriate recycling procedures for this category are not substantially distinct from those for the previous category [403,406].

**Figure 19:**
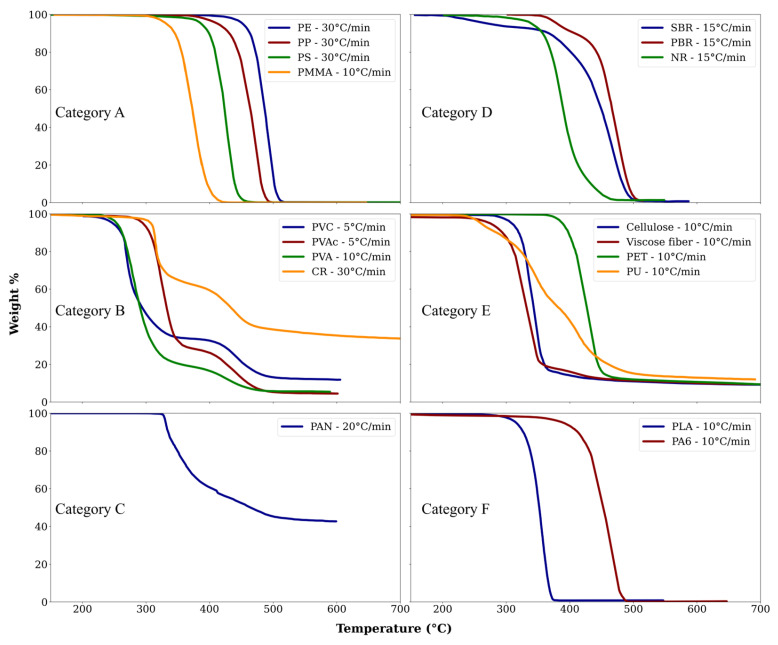
Thermogravimetric analysis graphs of different polymers according to Figure 17 [392,393,394,395,396,397,398,399,400,401,402,403,404,405,406,417].

To further highlight the different overall degradation mechanisms for the six categories, Figure 19 shows variations in their TGA curves.

#### 3.5.2. Biolysis

Currently, biological recycling methods for textile materials focus solely on natural and man-made fibers. Synthetic plastics have thus not been efficiently processed through these methods. Biological recycling processes encompass a range of techniques, such as enzymolysis to produce glucose and fermentation to generate bioethanol. Compared to lignocellulose, waste textiles can be biodegraded without issues caused by lignin and hemicellulose, but the high crystallinity of cellulose in cotton fibers presents a significant challenge. An overview of textile recycling methods using enzymolysis is provided in Table 4. Below, some complementary examples are discussed in greater detail.

Despite concerns over the environmental impact of textile waste containing synthetic polymers and cellulose fractions, the cellulose component holds promise for generating biofuels, such as EtOH and biogas. Textile waste typically contains 35–40% cellulose, making it a potential feedstock for producing biological products [418]. Cotton present in textile waste can serve as an alternative source of renewable energy and has been studied as a feedstock for bioethanol, biorefining, and biogas production [419]. To enhance enzyme accessibility, it is recommended to increase the surface area of cellulose feedstocks by modifying their porosity and reducing their crystallinity through pretreatment.

Notably, textile mill cotton waste has already been subjected to acid and alkali pretreatment to expose sugars for enzymatic hydrolysis by Fusarium species’ cellulase. The enzyme activity was enhanced, and the glucose release was higher with acid pretreatment compared to alkali pretreatment. Glucose was fermented with *Saccharomyces cerevisiae* through simultaneous saccharification and fermentation. In batch fermentation, 11.8 mg·mL^−1^ of alcohol was produced [420].

The advantages of enzymolysis are that the cellulose fraction becomes soluble and can be separated from other fractions in the textile blend without the need for manual sorting. According to Wang et al. [421], the solubility of cellulose in a cold NaOH/CH_4_N_2_O solution can be enhanced by enzymatic pretreatment. They observed an increase from 30% to 65%, which was primarily linked to the enzymatic treatment reducing the average molar mass of cellulose.

Vecchiato et al. [422] developed a circular economy solution for recycling viscose fiber waste. They used glucose to produce bioethanol and a flame-retardant pigment to produce new fibers. The applied enzymatic hydrolysis method allowed for the recovery of both glucose and pigment in high yields of 98% and above 99%, respectively. The maximum amount of released sugars was recovered after 8 h at 50 °C, and this recovered glucose was successfully used to produce EtOH through *S. cerevisiae*. The EtOH yield upon using commercial glucose was almost identical to that upon using recovered glucose.

Studies on the fiber morphology and enzymatic hydrolysis yields revealed that while alkali pretreatment of cotton fiber waste was necessary, it was not required for viscose fiber waste. Viscose fiber outperformed cotton fiber in terms of enzymatic hydrolysis and produced higher fermentation yields (8.1 vs. 6.9 g·L^−1^) due to the variations in their microcrystalline structures [423]. MMCFs appear to be the favored type of fiber in the realm of biological recycling due to their ability to be recycled with minimal expense and environmental impact compared to other fibers.

Biological recycling methods have also been explored for wool, a complex natural fiber composed mainly of proteins (97%) and lipids (1%). This fiber material is an ideal substrate for several classes of enzymes, such as proteases and lipases, which increase its biodegradation rate [423]. For example, Navone and Speight [424] suggested enzymatic treatment of wool/polyester fabric blends as a means of selectively breaking down wool fibers while preserving synthetic fibers. This involved the use of a protease with keratinolytic activity, along with reducing agents applied to the fabric blends. The most effective results could be obtained through a two-step enzymatic process in the presence of sodium thioglycolate. The polyester fibers could then be recycled into polyester yarn, which could be utilized in the production of new clothing items or other products [425].

Quartinello et al. [426] even demonstrated the efficacy of stepwise enzymatic hydrolysis of cellulose–wool–polyester blends. In this process, textile waste was sequentially treated with (1) protease to extract amino acids from wool components with 95% efficiency, and (2) cellulases to recover glucose from cotton and viscose constituents with 85% efficiency. The resulting amino acids and oligopeptides from wool degradation can serve as replacements for carbon and N_2_ sources, while the glucose from cotton hydrolysis can be fermented with *S. cerevisiae* to produce EtOH [423].

**Table 4:** Studies on the enzymolysis of different textile blends for the production of glucose.

Textile	Enzyme	Solvent for Pretreatment	Solvent:Polymer (vol/vol%)	T (°C)	t (h)	Buffer Solution	Glucose Yield (%)	Ref.
Cotton-based waste (jeans)	Cellulase and Tricoderma reesei	H_3_PO_4_	50:1	50	96	0.33 mol·L^−1^ sodium citrate (pH: 4.8)	86.1	[419]
Cotton linters	Celluclast	NaOH/CH_4_N_2_O solution	100:5	50	0.5–4	Acetate (pH: 4.5)	95.0	[421]
Dyed cotton and cotton–polyester blend shirts	Cellulase AP3	NMMO monohydrate, [BMIM]Cl, H_3_PO_4_, and NaOH/CH_4_N_2_O solution	10:1–20:1	50	72	0.1 M·L^−1^ phosphate (pH: 5) with 0.02% sodium azide	58.1	[427]
Polyester–cotton blend shirts	Cellusoft L	Lutensol AT20	NA ^1^	50	1	0.1 M·L^−1^ of acetate (pH: 5)	100	[428]
Undyed cotton T-shirt	Cellulase	[AMIM]Cl	100:5	50	NA	2.5 M·L^−1^ citrate (pH: 5)	86.0	[429]
Cotton–polyester blend	Celluclast 1.5 L and β-glucosidase	NaOH/CH_4_N_2_O solution	NA	50	96	0.2 M·L^−1^ sodium citrate (pH 5.1)	98.7	[430]
Cotton–PET blend	HiC and CTec2	Mechanical treatment	NA	55	24	Sodium phosphate (pH: 7.4)	83–87	[431]
Polyester–cotton blend	Celluclast 1.5 L and β-glucosidase	NaOH/CH_4_N_2_O, NaOH thiourea, and NaOH/CH_4_N_2_O/thiourea	95:5	45	72	Sodium acetate and 0.5 g·L^−1^ sodium azide (pH: 4.8)	91.0	[294]
Cellulose–wool–polyester blend	Cellic CTec3 and Savinase 12T	mQ-H_2_O	NA	50	48	0.67 M·L^−1^, Tris-HCl (pH: 9)	50–90	[426]
Bleached cotton fabric	Celluclast 1.5 L	Ultrasonic treatment	NA	50	1.5	0.2 M·L^−1^, acetate (pH: 5)	54.0	[432]

^1^ Not available.

#### 3.5.3. Pyrolysis

Pyrolysis has emerged as a pioneering approach in the transformation of waste textiles into valuable products, such as fuels and chemicals. In the realm of textile pyrolysis, catalysts exert a substantial impact on product distribution and selectivity. The inception of catalytic pyrolysis for synthetic textiles can be traced back to the early 2000s, when researchers embarked on exploring the potential of various catalysts to augment the pyrolysis process [433]. Over time, a myriad of catalysts, including zeolites [434], mesoporous materials [435], and metal oxides [436], have been tested to enhance the pyrolysis process. Pyrolysis is a suitable technique commonly used for polyolefin recycling, with direct and indirect processes used to produce light olefins [437,438,439,440]. The direct catalytic process for producing light olefins is being developed for industrial use but has challenges, and the dedicated transformation of this knowledge into that on the textile level is an ongoing research effort. In what follows, the main possibilities of pyrolysis are highlighted to provide general insights based on current data and case studies, and for which textile materials pyrolysis is a worthwhile option is explained. Table 5 provides an overview of the research conducted in the field of pyrolysis and gasification.

An important case study is that of Eschenbacher et al. [361], who achieved 85% recovery of light olefins using modified zeolite catalysts in a micropyrolysis reactor. The indirect process leading to pyrolysis oil is preferred by large refineries and petrochemicals but requires pretreatment due to high levels of olefins and cyclic compounds [439]. Kusenberg et al. [388] and Abbas-Abadi et al. [380,441] studied polyolefin pyrolysis and evaluated the challenges of producing light olefins from pyrolysis oil as well as the effect of the presence of mineral and organic contaminations in the oil on product yields and coke formation.

**Figure 20:**
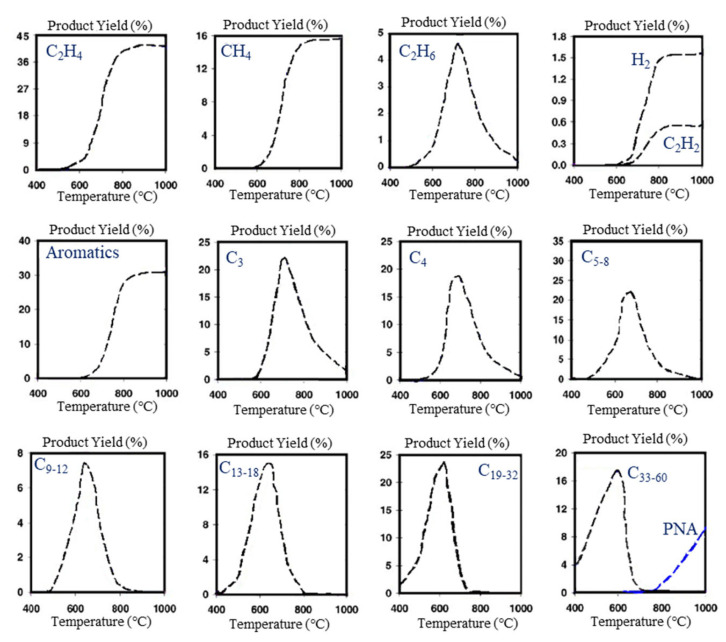
The yields of PE pyrolysis products vs. temperature (adapted from [442]). PNA stands for polynuclear aromatics [442].

Green and Sadrameli [442] investigated the impact of temperature (400–1000 °C) on the products of high-density polyethylene (HDPE) pyrolysis using a continuous fluidized bed (FB) reactor (Figure 20). Complete conversion of PE was achieved at temperatures above 650 °C due to short residence times. At temperatures below 500 °C, C_1–4_ compounds were insignificant, and C_12+_ compounds were predominantly produced. The production of H_2_ and light hydrocarbons started to increase at 500 °C and reached the maximum at 700 °C. Ethylene presented a maximum peak at 900 °C, and methane (CH_4_) increased in the studied temperature range. Interestingly, the propylene and butadiene yields decreased significantly with a slight increase in temperature above 700 °C. The production of aromatics indicated an increasing trend beyond 650 °C, whereas polyaromatics appeared at 800 °C.

The residence time is one of the key parameters that must be considered in the reactor design. It should be long enough to obtain a sufficient conversion of the feedstock into lighter products but short enough so that light olefins are efficiently removed from the reactor, thereby preventing secondary reactions, similarly to extrusion-based modifications [443]. The produced light olefins can act as intermediates in bimolecular reactions and are then converted into aromatic compounds and coke [439,440,444].

**Figure 21:**
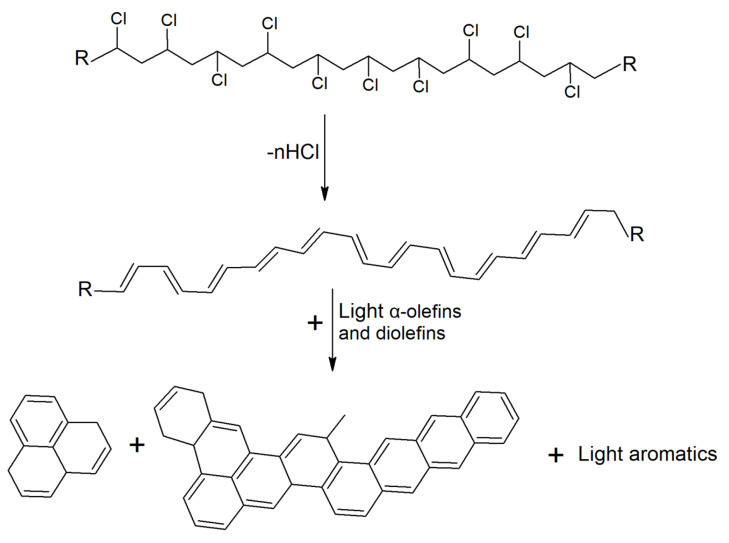
Diagram illustrating PVC degradation and the subsequent reactions with light olefins during the pyrolysis of waste polyolefins [441].

The pyrolysis products of mixed polyolefins (MPOs), which make up a third important case study, are significantly impacted by important parameters, such as the PP/PE ratio, the amounts of other polymers (e.g., PS, PET, PVC, and rubbers), and the presence of mineral and organic contaminants. PVC contamination is particularly noteworthy due to its potential for HCl release and residual structure, which can significantly contribute to coke formation [445]. The polyene left after the release of HCl is a viable option for absorbing light olefins and styrene and for enhancing secondary reactions, such as Diels–Alder reactions (Figure 21). During the degradation of biomass contamination and after deoxygenation, it exhibits characteristics similar to those of PVC and can significantly contribute to coke formation [379].

In any case, the design of a suitable pyrolysis reactor is an important factor in the production of light olefins from MPO waste. The optimal reactor design should have proper feeding, minimum residence time, and proper heat and mass transfer with minimum energy consumption [446]. Different reactor designs, such as vortex [387,447,448], conical spouted bed reactor [449], downer [450], and FB reactors [451], are being investigated. The use of modified zeolite catalysts (promotion, steaming, and mesoporosity) can increase product selectivity and light olefin production with lower energy consumption. Designing robust catalysts remains a challenge for the large-scale implementation of catalytic pyrolysis [391].

**Figure 22:**
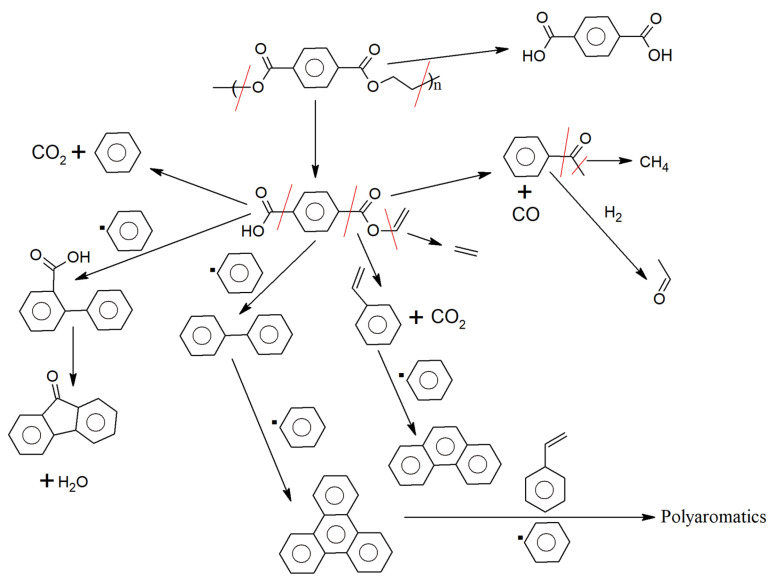
The proposed mechanism of PET degradation at elevated temperatures [411,452,453].

Figure 22 illustrates the typical degradation reactions of PET during pyrolysis [411,452,453]. At high temperatures, the C–O bonds are susceptible to breaking due to their weak bonding energy. The acidic groups attached to benzene (C_6_H_6_) are affected in the subsequent stages. The primary pyrolysis products of PET are C_6_H_6_, CO_2_, and carbon monoxide (CO), as shown in Figure 22. Although some TPA may be produced during pyrolysis, a portion of it will quickly undergo secondary reactions. The formation of coke during PET pyrolysis can be attributed to the presence of phenyl radical groups and styrene (e.g., from contaminants). This leads to subsequent secondary reactions, such as carbonization, Diels–Alder reactions, and ultimately, coke formation (polyaromatics). Process parameters are essential to minimize coke formation during PET and cellulose pyrolysis through fast pyrolysis and using reactors with a minimum (average) residence time [411,452,453].

It should be stressed that pyrolysis is not a suitable method for PET recycling, due to the high production of gases (CO_2_ and CO) therein, and also for other polycondensation polymers. Pyrolysis is a viable option in situations where techniques such as mechanical recycling and solvolysis prove to be unresponsive. Studies have indicated that incorporating catalysts such as iron oxyhydroxide, rutile (titanium dioxide [TiO_2_])/SiO_2_, Zeolite Socony Mobil–5 (ZSM-5), and sulfated zirconia into PET pyrolysis can effectively suppress TPA formation [454]. Below, some key examples of pyrolysis-based research with textile materials are given.

Molto et al. [452] applied a laboratory-scale batch horizontal tube reactor to pyrolyze PET fibers within a temperature range of 650 °C to 1050 °C. The primary products were mostly C_6_H_6_ (2.42–30.09 wt%), CO_2_ (14.85–27.70 wt%), and CO (8.73–16.49 wt%), while ethylene (0.39–3.88 wt%) and CH_4_-ethane mix (1.71–5.70 wt%) were secondary products. The pyrolysis process generated a diverse assortment of compounds, such as aromatics, linear olefins and paraffins, acids, and phenols [452]. Moreover, the formation of organic acids (e.g., TPA and benzoic acid [C_6_H_5_COOH]) as a process issue can cause corrosion and line blockages [453]. During pyrolysis, PET goes through primary and secondary reactions, leading to different products and by-products at high temperatures.

Kumagai et al. [454] conducted a study on the reactivity of PET pyrolysis products with different types of metal oxides. They utilized a Tandem μ Reactor-GC/MS and carried out PET pyrolysis at 450 °C. The results showed that zinc oxide (ZnO), which has a high base strength, effectively promoted the decarboxylation of the principal pyrolysis products of C_6_H_5_COOH and TPA at a low temperature of 700 °C, resulting in the production of C_6_H_6_-rich aromatic hydrocarbons (88.8 area%). On the other hand, MgO, TiO_2_, and zirconium dioxide were found to have lower base strengths than ZnO; hence, a temperature increase of around 50−70 °C is required for decarboxylation. These findings provide valuable insights into feedstock recycling and converting PET waste materials or mixed plastics that contain PET into raw chemical materials through catalytic pyrolysis.

Diaz-Silvarrey et al. [455] pyrolyzed PET using a sulfated zirconia (SZ) catalyst at different temperatures and catalyst/PET ratios. The findings indicate that both temperature and the catalyst/PET ratio significantly affect the production of C_6_H_5_COOH, a widely used precursor in the food and beverage industry. A high temperature (600 °C) and the absence of a catalyst resulted in a 16% increase in the recovery of C_6_H_5_COOH in the wax product compared to other conditions. However, this process is energy-intensive due to the endothermic nature of PET pyrolysis. The addition of the catalyst increased the amount of light hydrocarbons (C_1_–C_4_) produced, from 4 wt% without catalyst to 20 wt% at a 10 wt% catalyst/PET ratio. Nevertheless, the catalyst deactivated due to coke deposition on the catalyst surface and partially decomposed at high pyrolysis temperatures (>525 °C). It was thus suggested that PET catalytic pyrolysis using the SZ catalyst should be performed at temperatures below 525 °C, with catalyst loads below 10 wt% of PET, to obtain high yields of C_6_H_5_COOH and a high value of gas products (i.e., a high proportion of hydrocarbons).

Jia et al. [434] performed catalytic pyrolysis of PET using ZSM-5 zeolite and nickel chloride (NiCl_2_) as catalysts at various temperatures under a N_2_ atmosphere. The results revealed a significant reduction in carboxyl and aliphatic hydroxyl groups in the waxy residue upon zeolite utilization during pyrolysis. A 22% increase in aromatic hydroxyl groups and a 42% decrease in carbonyl groups were observed under the catalyst/PET ratio of 2/1, demonstrating enhanced deoxygenation effects with zeolite addition. Meanwhile, the addition of ZSM-5 and NiCl_2_ reduced the waxy product yield while increasing the gas yield. The use of ZSM-5 had a limited impact on the primary decomposition of PET but promoted secondary volatile reactions, whereas NiCl_2_ enhanced primary decomposition toward liquid products.

Sulfated zirconia has also been recognized as a promising catalyst for obtaining C_6_H_5_COOH from PET. Diaz-Silvarrey et al. [455] reported the highest C_6_H_5_COOH yield of 28 wt% at 600 °C, using a catalyst/feed ratio of 1/10. Additionally, a Co-based catalyst (Co/SiO_2_) could be employed to transform hazardous volatile compounds, such as C_6_H_6_ derivatives and polycyclic aromatic hydrocarbons (PAHs), into syngas. Compared to non-catalytic pyrolysis, CO_2_-assisted catalytic pyrolysis of waste PET yielded over three and eight times higher H_2_ and CO production, respectively. Furthermore, CO_2_-assisted catalytic pyrolysis suppressed carbon deposition on the Co-based catalyst [456].

Besides PET, other common types of plastics found in textiles, such as PE and PP, have been identified as ideal sources for the production of light olefins, as previously discussed [361,457]. However, it should be noted that the catalyst/feed ratio for converting plastics into light olefins was relatively high. Optimizing the conversion of textile waste into valuable bio-oil and gases, along with minimizing catalyst consumption and pyrolysis time, is thus crucial.

Yousef et al. [458] examined the pyrolysis process of waste jeans, a substantial fraction of textile waste, employing heavy metals present in textile dyes as self-catalysts to improve conversion efficacy. A two-stage method—initially performing lab-scale experiments utilizing TGA-FTIR and subsequently conducting pilot-scale assessments in a specially designed pyrolysis reactor—was performed. The results revealed a successful conversion of over 82% of the feedstock into liquid-gas products, with the untreated feedstock generating a 20% higher bio-oil yield compared to the samples without heavy metals.

Furthermore, Phan et al. [459] focused on the pyrolysis of a textile sample made up of mostly cotton, with a small amount of polyester (<5%). The study included experiments at five temperatures, ranging from 350 °C to 700 °C, using a 150–200 g sample for each test. To begin each test, the sample was placed in a fixed bed reactor at room temperature and heated at a rate of 10 °C·min^−1^ until the target temperature was reached, after which it was maintained at that temperature for 2 h. The results showed that the percentage of char decreased from 32% to 19% with rising temperature, while the liquid fraction reached its highest yield of 47% at 600 °C, and the gas yield increased with rising temperature. Phenolic, furan, and anhydrosugar compounds were the predominant products in all reactions, whereas the major gas products were CO_2_, CO, CH_4_, and H_2_.

Zhang et al. [435] performed ex situ catalytic pyrolysis of waste mixed clothing using hierarchical ZSM-5 and commercial CaO as catalysts. Pyrolysis in the temperature range of 450~750 °C primarily resulted in levoglucosan formation. The use of HZSM, possessing Brønsted/Lewis acid sites on microporous and mesoporous structures, contributed considerably to the production of monocyclic and dicyclic chemicals. Moreover, CaO exhibited strong deoxygenation capabilities, facilitating the transformation of the waste into low oxygen-containing chemicals, such as ketones, aliphatic hydrocarbons, and aromatics. This study underscored the potential of catalytic upgrading of waste mixed cloth into valuable chemicals through catalytic pyrolysis over modified ZSM and CaO catalysts.

The valorization of waste teabags through catalytic pyrolysis, examining the effects of CO_2_ and HZSM-11 on pyrolytic products, was investigated by Kim et al. [460]. In non-catalytic pyrolysis, the carrier gas of CO_2_ increased the pyrolytic gas yields while reducing the formation of C_6_H_6_, phenolics, and PAHs compared to N_2_. The HZSM-11 catalyst further enhanced CO_2_ conversion to CO, particularly in a CO_2_ environment. The synergistic use of CO_2_ and HZSM-11 in waste teabag catalysis increased caprolactam production. However, to ensure economic feasibility, a proper waste collection and treatment method, such as separating waste teabags from food waste streams and drying at the source, is essential prior to processing.

More recently, Lee et al. [461] investigated the conversion of waste medical masks (mainly PP and some polyamide), by-products of the COVID-19 pandemic, into value-added chemicals, such as BTEX (C_6_H_6_, toluene, ethylbenzene, and xylene), via catalytic fast pyrolysis using zeolite catalysts. The effect of zeolite properties on pyrolytic product yields and composition was assessed using different catalysts (HBeta, HY, and HZSM-5). The large-pore zeolite catalysts HBeta and HY exhibited 134% and 67% higher BTEX concentrations than HZSM-5, respectively. The mesoporous structures of the catalysts allowed the branched hydrocarbons formed during mask pyrolysis to diffuse within the catalyst pores to convert into aromatic compounds. To recover more valuable gaseous products, multistage catalytic pyrolysis over Ni/SiO_2_ was employed to enhance H_2_ and CH_4_ formation by breaking the C–C and C–H bonds in long-chain hydrocarbons [462]. CO_2_ carrier gas acted as both a reaction medium and a soft oxidant, controlling the H_2_/CO ratio in the product gas. These pioneering investigations demonstrated the potential for converting waste masks into valuable chemicals through catalytic pyrolysis.

**Table 5:** Key contributions to pyrolysis and gasification for textile materials and waste.

Feed	Carrier Gas	Pyrolysis T (°C)	Catalysis T (°C)	Reactor	Catalyst	Cat/ Feed	Product Yield	Major Findings	Ref.
Polyamide	CO_2_	700 (10 °C·min^−1^)	600	Fixed bed	Ni/SiO_2_	1	Syngas:	The production of syngas from CO_2_-assisted catalytic pyrolysis was seven times larger than the inert atmosphere of N_2_ arising from increased CO formation through the catalytic reaction of CO_2_ with pyrolytic products over the catalyst. CO_2_ also offered a great opportunity to suppress catalyst deactivation by suppressing coke deposition or removing the coke deposited on the catalyst surface.	
							CO: 17.5 mol%		
							H_2_: 11.9 mol%		
	N_2_	700 (10 °C·min^−1^)	600	Fixed bed	Ni/SiO_2_	1	Syngas:		
							CO: 5.7 mol%		
							H_2_: 18.7 mol%		
PET	N_2_	600 (45 °C·min^−1^)	600 (45 °C·min^−1^)	Tubular	Sulfated zirconia	0.1	Benzoic acid: 28.0 wt%	Increasing the catalyst/plastic ratio could increase the amount of other valuable products (e.g., light hydrocarbons [C_1_–C_3_]) recovered in the gas.	[455]
PET	N_2_	600	600	Tubular	ZSM-5	6	Solid residual: 10.5 wt%	ZSM-5 has little effect on the primary decomposition of PET, but it promotes the secondary volatile reactions. The use of NiCl_2_ as a catalyst will greatly improve the primary decomposition of PET to generate more liquid products.	[434]
							Wax: 23 wt%		
	N_2_	600	600	Tubular	ZnCl_2_	1	Wax: 68.9 wt%		
HDPE	N_2_	500	500	Spouted bed + fixed bed	HZSM-5	8	Light olefins (C_2_~C_4_): 59 wt%	The low residence time in the catalytic reactor enhances the selectivity of light olefins and attenuates the secondary reactions of coke formation.	[457]
LDPE ^1^	He	550	600	Micropyrolyzer	Phosphorus-modified and steam-treated HZSM-5	80	Light olefins (C_2_~C_4_): 82.8 wt%	Phosphorus-modified and steam-treated HZSM-5 showed almost no deactivation due to the lower coking propensity during 130 runs, with stable conversion to C_5+_ aliphatics and high C_2_–C_4_ olefin selectivity (~75%) using post-consumer mixed polyolefins.	[361]
23 wt% LLDPE ^2^, 7.5 wt% LDPE, 29.5 wt% HDPE, 40 wt% PP	He	550	600	Micropyrolyzer	Phosphorus-modified and steam-treated HZSM-5	80	Light olefins (C_2_~C_4_): 78.8 wt%		
Mixed polyolefins	He	550	600	Micropyrolyzer	Phosphorus steamed HZSM-5	80	Light olefins (C_2_~C_4_): 73.2 wt%		
Waste textiles	N_2_	800 (10 °C·min^−1^)	800 (10 °C·min^−1^)	Fixed bed	Al_2_O_3_	-	Oil: 37.5 wt%	Heavy metals in textile dyes were used as self-catalysts to accelerate the conversion process and decrease the pyrolysis time by 15% and to increase the bio-oil yield by ~20% compared to the textile samples treated without catalysts.	[458]
							Gas: 44.7 wt%		
							Char: 17.8 wt%		
Waste textiles (main component: PET)	CO_2_	720 (10 °C·min^−1^)	650	Tubular	Co/SiO_2_	1	Gas: 80.9%	Catalytic pyrolysis over a Co-based catalyst with threefold- and eightfold-higher production of H_2_ and CO, respectively, compared to non-catalytic pyrolysis. This process also suppressed catalyst deactivation, converting more than 80 wt% of waste textile into syngas and CH_4_.	[456]
							Char: 17.6%		
	N_2_	720 (10 °C·min^−1^)	650	Tubular	Co/SiO_2_	1	Gas: 73.9%		
							Char: 19.1%		
Waste mixed cloth (cotton fibers, acrylic fibers, and PET fibers)	He	650	650	Pyroprobe	HZSM-5	6	Aromatics: 98.9%	The utilization of HZSM with Brønsted/Lewis acid sites on microporous and mesoporous structures significantly contributed to the production of monocyclic/dicyclic chemicals, mainly referring to monoaromatics and naphthalene-based derivatives.	[435]
	He	650	-	Pyroprobe	-	-	Anhydrosugars: 45.7%		
COVID-19 mask	N_2_	550	550	Fixed bed	Hbeta	-	BTEX: 49.4%	The pore sizes of Hbeta and HY were larger than the kinetic diameters of the branched hydrocarbons, allowing the thermally derived branched hydrocarbons to diffuse inside the pores and thereby converting into the aromatic hydrocarbons over acid sites located mainly inside the pores.	[461]
	N_2_	550	550	Fixed bed	HY	-	BTEX: 35.2%		
	N_2_	550	550	Fixed bed	HZSM-5	-	BTEX: 21.1%		
COVID-19 mask (PP, PE, polyamide)	N_2_	600 (10 °C·min^−1^)	600	Tubular	Ni/SiO_2_	1	H_2_: 55.1 mol%	Pyrolysis over a Ni/SiO_2_ catalyst led to the substantial conversion of longer-chain (≥C_2_) HCs into H_2_ and CH_4_.	[462]
							CH_4_: 18.2 mol%		

^1^ LDPE, low-density polyethylene; ^2^ LLDPE, linear low-density polyethylene.

#### 3.5.4. Gasification

High plastic purity is necessary for both mechanical recycling and solvolysis, requiring careful sorting of the textiles to be recycled. However, if textile sorting is difficult and uneconomical, and none of the chemical processes are effective, gasification could provide a viable alternative.

Gasification is a process whereby a carbon-rich feedstock reacts with a gasifying agent, such as CO_2_, steam, or air, to produce syngas at relatively high temperatures (exceeding 700 °C) [463,464]. During gasification, the fuel is broken down into CO_2_ and H_2_ within the temperature range of 550–1000 °C by introducing the gasifying agent in sub-stoichiometric quantities [465]. The process involves four stages: pyrolysis, oxidation, reduction, and tar reforming. Partial oxidation occurs in the process, and the amount of oxygen used is less than what is required for stoichiometric combustion. In the reduction stage, char reacts with steam or CO_2_ to produce CO and H_2_, while in the oxidation stage, char and volatile compounds react with oxygen to create CO_2_. The heat generated in the exothermic oxidation reactions provides energy for the endothermic reducing reactions. In the tar reforming step, tar reacts with steam to produce lighter hydrocarbons [466]. Important contributions are included in Table 5 due to their complementarity with pyrolysis.

Supercritical water gasification technology has become a subject of widespread interest for the clean utilization of fossil energy in recent years. By functioning as a solvent in its supercritical state, nonpolar water can promote homogeneous reactions. Moreover, water can act as a catalyst, boosting reaction rates due to the high ion production near critical points and the formation of H_2_ atom radicals at high temperatures.

Supercritical water is also particularly tolerant of wet materials that have not undergone drying pretreatment, which can decrease polycondensation and coking. Utilizing supercritical water to recycle textile waste is thus a promising prospect [467,468].

In what follows, some key examples of waste textile gasification are presented. Wu et al. [469] investigated the catalytic pyrolysis and gasification of textile waste using metal catalysts. The researchers examined the impact of metal catalysts on the gasification rates of textile waste in the presence of CO_2_ using an atmospheric TGA system. The temperature range of 300–500 °C for the pyrolysis process of textiles was investigated, while 800–1000 °C was maintained for char gasification. The results indicated that ZnO exhibited the strongest catalytic effect on mixed textiles during the pyrolysis process, while Fe_2_O_3_ had the highest catalytic effect during the char gasification process. Among the various catalyst loadings tested, 5 wt% ZnO loaded char showed the most significant enhancement of char pyrolysis reactivity.

Jeong et al. [470] performed gasification experiments on 10 types of plastic waste using a two-stage gasifier with AC. The gases produced had low tar levels and could be used as fuels in gas engines. Aliphatic plastics (polyolefins) produced lower tar content compared to plastics with aromatic rings (PET and PU). Furthermore, the chemical structure of the plastic material significantly affected the yield during gasification, with aromatic plastic materials producing higher char yields. The heteroatoms in the plastic structure reduced the char yield. Accordingly, PP had the highest H_2_ content (about 26 vol%).

Bai et al. [471] explored the impact of various operating conditions on the gasification of PET microplastics and discussed the gasification pathway. They found that increasing the reaction temperature and time promoted PET microplastic cracking, but the reaction pressure did not significantly affect gasification. Seawater utilization was an effective route for plastic gasification, and metal ions in seawater enhanced the process in different ways. The gasification kinetics results showed that biphenyl formation was facilitated for PET plastic and that its cracking was temperature-dependent, hindering gasification. The study achieved 98% carbon conversion at 800 °C and 23 MPa for 10 min.

Lee and Jung [436] investigated the gasification of polyamide through CO_2_-assisted catalytic pyrolysis using nickel (Ni)/SiO_2_ as a catalyst to enhance syngas (H_2_ and CO) production. The research also identified caprolactam, the PA6 monomer, as a recoverable product during pyrolysis, which has also been confirmed by other researchers [460]. Nonetheless, the separation of caprolactam from pyrolytic oil was complicated due to its complex composition. The Ni-based catalyst facilitated the conversion of CO_2_ into CO, promoting H_2_ production and pyrolytic oil degradation. The syngas yield from CO_2_-assisted catalytic pyrolysis was seven times higher than that in non-catalytic processes, which was attributed to the increased CO formation via catalytic reactions. Additionally, CO_2_ aided in mitigating catalyst deactivation by suppressing coke. This research suggests that CO_2_-assisted gasification presents an environmentally friendly approach to converting GHG and plastic waste into value-added syngas while reducing harmful emissions.

#### 3.5.5. Activated Carbon Production

The chemical structures of natural, semi-traditional, PET, and acrylic fibers make them prone to intensifying secondary reactions (e.g., Diels–Alder reactions and carbonization). In contrast to the pyrolysis approach, which necessitates fast pyrolysis and short residence time to limit secondary reactions, AC production involves a slow pyrolysis process with an extended residence time to enhance secondary mechanisms [472,473].

AC involves two methods: physical and chemical. Physical activation includes pyrolysis and carbonization in a neutral medium, followed by physical activation in an oxidizing atmosphere, such as steam and CO_2_, at temperatures ranging from 800 °C to 1100° C [474,475].

In chemical activation, the fiber or carbonized fiber is impregnated with a chemical agent and activated at a lower temperature range (400–900 °C) compared to the physical method. Activating agents mainly react with weak structures in AC, such as defective polyaromatics and functional groups, leading to increased pore formation. Activating agents also create new functional groups on the surface of AC while neutralizing previous ones [476]. Different acid, base, and salt activators, such as H_3_PO_4_ [477], H_2_SO_4_ [478], zinc chloride (ZnCl_2_) [479], potassium carbonate (K_2_CO_3_) [480], lanthanum ferrite [481], NaOH [482], and potassium hydroxide [483], react with degradable parts of feedstock before or after carbonization. Activators increase the activation rate and determine the efficiency and application of AC to some extent by creating specific functional groups on the surface. AC can also be produced in a single stage under an oxidizer atmosphere [484].

Fibrous AC has unique properties, such as distinctive pore structures, fast adsorption rates, a large surface area, specific surface functional groups, and ease of regeneration. Table 6 presents the review of ACs produced from different textile wastes, with key contributions highlighted in what follows. For example, Xu et al. [485] synthesized AC with high mesoporosity from waste PET by utilizing magnesium chloride (MgCl_2_) as an activator and precursor based on a MgO template. The most significant surface area (1307 m^2^·g^−1^) and total pore volume (3.56 cm^3^·g^−1^) were achieved with a mixing ratio of 5:5, a pyrolysis temperature of 900 °C, and a duration of 1.5 h. The results indicated that MgCl_2_ hindered the formation of tars through decarboxylic and dehydrogenation reactions, which led to the favorable formation of an open pore structure. Moreover, MgCl_2_ catalyzed the secondary reactions due to its Lewis acidity, which facilitated the formation of carbonaceous materials during pyrolysis.

Furthermore, Silva et al. [486] employed a slow pyrolysis technique with H_3_PO_4_ to produce fibrous AC from denim fabric waste, which was applied as an efficient adsorbent. H_3_PO_4_ was utilized as a protective agent to maintain the fibrous morphology of the precursor and introduce phosphorus-based functional groups onto the AC surface. The AC exhibited a considerable surface area of 1582 m^2^·g^−1^ and mesoporous features. The findings suggest that the adsorption process may involve chemisorption and can occur through multilayer adsorption.

Rabbi and Dadashian [487] conducted research on how the degree of stabilization, activation temperature, and activation stretching influence both the adsorption and mechanical properties of acrylic-based AC fibers. The ideal conditions for producing fibrous AC with high iodine adsorption and tensile strength were determined to be a stabilization degree of 56%, an activation temperature of 800 °C, and 1% stretching. Under these conditions, the experimental iodine adsorption and tensile strength values of the AC fibers were approximately 865 mg·g^−1^ and 423 MPa, respectively. Brunauer–Emmett–Teller analysis of the optimized sample revealed a surface area of 561 m^2^·g^−1^ with an average pore diameter of 2.74 nm. Polyacrylics that incorporate vinyl chloride as a comonomer exhibit greater stability than other types of polyacrylics. In addition, acrylic fibers have the potential to produce carbon fibers at a low cost.

**Table 6:** Overview of the contributions of AC fibers produced from different textile wastes.

Feed	Reactor	Activation Process		Properties				Ref.
		T (°C)	Heating Rate (°C·min^−1^)	BET ^1^ Surface Area (m^2^·g^−1^)	Micropore Area (m^2^·g^−1^)	Micropore Volume (cm^3^·g^−1^)	Total Pore Volume (cm^3^·g^−1^)	
Waste cotton	Tubular	750–900	5	1044–2562	-	0.41–1.14	0.41–1.35	[488]
Waste cotton	Tubular	700	5	292	255	0.11	0.14	[489]
Aramid woven fabric waste	Furnace	800–1200	300	109–248	-	-	-	[490]
Hemp and flax	Fixed bed	350–900	2	200–900	-	0.2–0.35	0.10–0.72	[491]
Acrylic waste	Fixed bed	700, 800, 900	5	752	-	0.32	-	[492]
Waste wool	Tubular	1100	2	152	-	-	0.15	[493]
PET waste	Muffle furnace	900	10	230–1336	193–589	0.08–0.29	-	[494]
PET waste	Muffle furnace	900	10	171–951	98–480	0.04–0.20	0.15–1.68	[495]
PET waste	Muffle furnace	900	10	171–1364	98–527	0.04–0.23	0.15–2.91	[496]
PET waste	Pipe furnace	900	10	483–1307	24–323	0.01–0.12	1.53–3.56	[485]
PET waste	Pipe furnace	650	10	382–1415	837–1071	0.32–0.43	1.45–2.10	[497]
Cotton wastes	Pipe furnace	400–500	10	510–1855	274–440	0.13–0.20	-	[498]

^1^ BET, Brunauer–Emmett–Teller analysis.

#### 3.5.6. Solvolysis

Solvolysis is a process primarily employed for the depolymerization of polycondensation polymers [415,499,500]. For example, the solvolysis reactions of PET involve the use of various chemicals, such as CH_3_OH (methanolysis) [501], EG (glycolysis) [502], water (hydrolysis) [503], ammonia (NH_3_; ammonolysis) [504], and amines (aminolysis) [505], to facilitate the degradation of compounds. Note that, in complementary reductive depolymerization, the solvent and material used can be different and are generally different from those used in other common methods. As the reaction still necessitates solvent-based media, it has been classified within solvolysis.

**Figure 23:**
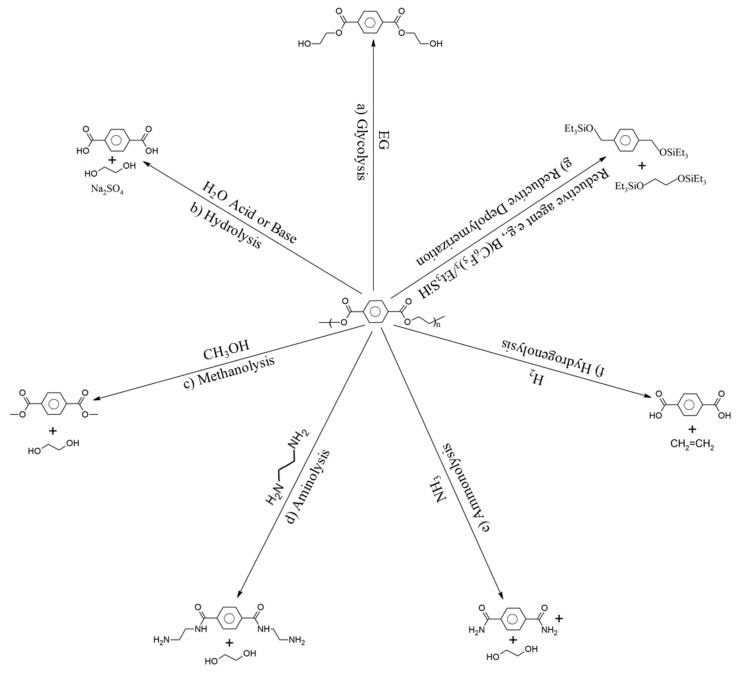
Examples of PET depolymerization via solvolysis.

Generally, the solvolysis process with high monomer recovery is facilitated by low temperatures and high pressures. For example, solvolysis is a promising method for PET fiber recycling, resulting in high-quality monomers without any toxic by-products [506]. Figure 23 shows the common types of PET solvolysis.

In contrast, the commonly reported methods for solvolysis of polyamide, such as hydrolysis and aminolysis, require harsh conditions, including high temperatures of 250 °C to 300 °C. Other methods, such as glycolysis or aminoglycolysis, have been less effective in regenerating polyamides. The presence of robust H_2_ bonds in polyamide hampers depolymerization, resulting in inferior performance compared to polyesters. Hence, there is a need to develop new, efficient, and sustainable depolymerization methods for polyamides to improve their recycling [507].

A high-level overview of the solvolysis methods for polyamide and PET is provided in Table 7, followed by a more detailed discussion, including key examples of prior investigations.

##### Glycolysis

Glycolysis is the most cost-effective and commercially viable process for the chemical recycling of PET bottles. It uses excess glycol, commonly EG, to produce BHET (Figure 23a) and its oligomers for a 180–240°C temperature range and considering a suitable pressure to maintain EG in the liquid state. The mechanism of glycolysis consists of several steps. Glycol first diffuses into the polymer and swells it. The diffused glycol then reacts with an ester bond in the chain. The swelling increases the diffusion rate as well as the reaction rate of glycolysis. The reaction rate increases with the polymer surface area. Hence, it is better to make the PET chip smaller through grinding, cutting, etc. BHET is generally purified by melt filtration under pressure and treated with AC to remove impurities and color [414].

The limited efficiency of the non-catalytic glycolysis of PET has prompted increased interest in utilizing catalysts for this process. Thus, various metal salts and organocatalysts have been used to enhance the reaction rate. Currently, the most frequently utilized catalysts for PET degradation are multiphase catalysts made of metal oxides (e.g., ZnO, manganese oxide, cobalt oxide, and zinc manganese oxide) and homogeneous catalysts made of metal acetates (e.g., zince [Zn], Mn, and Co), ILs ([Bmim][Cl], [Bmim][AlCl_4_], and [Deim][Zn(OAc)_3_]), while zinc acetate exhibits more activity for PET depolymerization [521].

Wang et al. [534] demonstrated that 1,3-dimethylimidazolium-2-carboxylate can serve as an organocatalyst for PET glycolysis. Using a mass ratio of 50/5/0.75 for EG/PET/catalyst and 20 mol% of 1,3-dimethylimidazolium-2-carboxylate relative to the monomer, post-consumer PET was depolymerized completely within 1 h at 185 °C. Upon cooling, the reaction mixture yielded up to 60% BHET as a crystalline precipitate. Compared to the basic IL (i.e., 1,3-dimethylimidazolium acetate), zwitterionic carbonate (i.e., 1,3-dimethylimidazolium-2-carboxylate) proved to be a much more effective catalyst. PET degradation was initiated more quickly at lower temperatures, leading to complete PET degradation within 1 h using 1,3-dimethylimidazolium-2-carboxylate. In contrast, with 1,3-dimethylimidazolium acetate, only around 35% degradation was achieved after 1 h, and up to 3 h were necessary to achieve a similar degree of depolymerization.

Typically, the glycolysis approach is utilized to recycle top-notch PET bottles and fibers. As a preliminary measure, partial glycolysis can be implemented, which involves the use of EG molecules to break down PET molecules into oligomers with a reduced average molar mass. This process of glycolysis between PET bottles and EG is commonly carried out through reactive extrusion. The resulting low-molar-mass oligomers can be utilized as raw material for subsequent steps, such as methanolysis or hydrolysis, or for the repolymerization process [535]. Furthermore, during glycolysis, the undesired by-product diethylene glycol, which is a dimer of EG, is produced, posing challenges in the separation and purification of the intended product.

The recycling challenges associated with specific fiber blends, such as polycotton, elastane with polyester, and elastane with cotton, differ from those associated with other fibers and textiles. Polycotton, a blend of polyester and cotton, presents significant difficulties due to its combination of natural and synthetic fibers, complicating the separation and recycling processes. Methods such as dissolution or glycolysis are often required to separate the polyester from the cotton, but these processes tend to be both energy-intensive and costly, and they must be selective to one of the fibers. Likewise, blends that include elastane with polyester or cotton introduce additional challenges because elastane’s elasticity complicates mechanical recycling, often leading to downcycling or disposal in landfills [59,536,537,538].

To overcome the aforementioned challenges, solutions such as selective dissolution or solvolysis, which targets specific fibers within a blend, or enzymatic methods that break down natural fibers while leaving synthetic ones intact can be considered. The selective removal of one of the fibers or blends can then precede mechanical recycling or chemical recycling techniques. Additionally, recycling could involve techniques such as thermal or chemical processes in which separation can occur after depolymerization [228,289,536].

##### Hydrolysis

Hydrolysis of PET chains produces hydroxyl and carboxylic end groups. A water molecule attacks the chain and causes its scission, creating a carboxylic end group. Although hydrolysis has been actively studied, it is still not a commercially viable option. One major obstacle is the purification problem of recycled TPA. It is difficult to purify TPA from the reaction mixture due to its low solubility and vapor pressure. Some impurities in PET waste that are not soluble in hydrous agents are not separated by transcrystallization using CH_3_COOH/H_2_O [539].

The depolymerization process involves using water to break down PET chains into TPA and EG, which can then be used in TPA-based PET synthesis plants. This process can be carried out under neutral, acidic, or basic conditions, as shown in Figure 23b.

Hydrolysis of PET with water or steam under high pressure and temperature can produce TPA and EG at a neutral pH. The hydrolysis rate is higher in a molten state than in a solid state. Metal salts, such as zinc acetate and sodium acetate, can catalyze hydrolysis reactions. Neutral hydrolysis does not produce undesirable liquid effluents and is not corrosive. However, PET impurities require intensive purification for pure TPA. Purification can be achieved through filtration, crystallization, extraction, or distillation of TPA and EG [535].

Several contributions have described a process for the acidic hydrolysis of PET using concentrated H_2_SO_4_ at atmospheric pressure and temperatures ranging from 25 °C to 100 °C. The resulting hydrolysis product is then neutralized with NaOH, leading to the formation of the corresponding TPA sodium salt (Na_2_SO_4_), which is water-soluble. In the final stage of the process, the resulting solution is acidified again to reprecipitate TPA with 99% purity. This process requires the separation of large amounts of concentrated H_2_SO_4_ and the purification of EG containing H_2_SO_4_, which significantly increases the cost of the process [50,540,541].

Ügdüler et al. [542] investigated the aqueous alkaline hydrolysis of pure PET and post-consumer PET waste to obtain EG and TPA. They found that PET depolymerization had the highest yield at 80 °C, with a particle size lower than 500 µm and after 20 min in a solution containing 40:60 vol% H_2_O:EtOH and 5 wt% NaOH. They also found that the PET conversion rate decreased with an increase in particle size, with the highest conversion rate obtained from monolayer PET films at the smallest particle size. The proposed alkaline hydrolysis process is promising for the chemical recycling of complex PET plastic waste, but the parameters need to be adjusted for environmental sustainability.

Furthermore, polycotton, a popular material used in hospital and hotel service textiles, such as sheets and towels, is a blend of cotton and PET. Recycling cotton-based textiles is a challenge due to their common blending with PET in various ratios, resulting in a structured blend that can hinder enzymes’ access to cotton. Compared to unblended cotton, blended cotton has greater crystallinity and polymerization because it has a higher average molar mass and intermolecular and intramolecular bonds [543].

Palme et al. [544] specifically examined a simple lab-scale method to hydrolyze PET in polycotton waste. The process involved the use of 5–15 wt% NaOH in water at temperatures ranging from 70 °C to 90 °C, which degraded PET to TPA and EG. The process generated three output streams: cotton, TPA, and filtrate containing EG and process chemicals. PET hydrolysis could be achieved in a 10% NaOH solution at 90 °C in just 40 min with the addition of a phase-transfer catalyst (benzyltributylammonium chloride). The yield of cotton cellulose was high, up to 97%, depending on the duration of the sample treatment. While the phase-transfer catalyst was not necessary for separation, longer treatment times were required, resulting in more cellulose degradation.

The use of acidic or basic conditions for hydrolysis can lead to corrosion and pollution issues. Additionally, the formation of TPA and acidic catalysts can promote the production of diethylene glycol [545]. Despite ongoing research, hydrolysis is currently not a feasible option for commercial use due to the difficulty of purifying recycled TPA. Purification of TPA from the reaction mixture is challenging due to its low solubility and vapor pressure. Moreover, the use of CH_3_COOH and H_2_O for transcrystallization may not effectively separate impurities in PET waste that are insoluble in aqueous agents.

The reduction in the degree of polymerization of cellulose by breaking down its β-1,4-glycosidic bonds is known as cotton depolymerization. Various methods can be employed for depolymerizing cotton fibers, such as hydrolysis, enzymolysis, and fermentation. Hydrolysis is affected by several factors, such as the heating source, the type of solvent (acid, base, or neutral), and the process’s duration. Cotton is transformed into glucose and solid fiber residues through the hydrolysis process. This process depolymerizes cellulose completely into its monomer, glucose, which can be used to create other chemicals, such as EtOH, organic acids, cellulose nanocrystal (CNC), and microcrystalline cellulose (MCC) [546].

Sanchis-Sebastiá [547] conducted a two-step acid hydrolysis process on cotton waste to extract glucose. The first step involved dissolving textile waste in a concentrated H_2_SO_4_ solution at a concentration of 60–80 wt% and a temperature of 30 °C for 1 h. The second step used a dilute H_2_SO_4_ solution at a concentration of 5 wt% and a temperature of 121 °C for 1 h in an autoclave to fully break down the cotton and form glucose. After these steps, the mixture was vacuum filtered to obtain liquid and solid fractions for further analysis. The two-step approach resulted in a high glucose yield of around 80–90%, which could not be achieved using concentrated or dilute H_2_SO_4_ alone.

CNC and MCC possess distinct mechanical, optical, and thermal characteristics that render them appealing for a diverse range of applications in industries such as biomedical engineering, electronics, and packaging. CNC can be obtained through different methods, including the use of a H_2_SO_4_/HCl aqueous solution, 60 wt% H_2_SO_4_, and 2,2,6,6-tetramethylpiperidine 1-oxy (TEMPO) oxidation. CNC has been produced from bleached white cotton fabric and indigo-dyed denim fabric via 60 wt% H_2_SO_4_ or TEMPO oxidation. Acid hydrolysis produced CNC yields of 40% and 38% for bleached cotton and denim fabrics, respectively, whereas oxidation generated greater yields of 73% and 79% [548]. Acid hydrolysis and oxidation were also used to extract CNC from cotton fabric, with yields of 90% and 60%, respectively. MCC was obtained with phosphotungstic acid at 140 °C for 6 h, achieving a high yield of 83.4% [548].

Kamimura et al. [532] developed a new technique for depolymerizing PA6 into caprolactam using hydrophilic ILs [emim][BF_4_]. The process was significantly enhanced with the addition of catalytic quantities of N,N-dimethylaminopyridine [DMAP]. Under microwave radiation at 300 °C, PA6 was effectively depolymerized in hydrophilic ILs, with complete conversion within 1 h. As a result of this reaction, by utilizing a catalyst (10 wt%), the recovery rate of [emim][BF_4_] was found to vary from 82% to 89%, while the recovery rate of caprolactam as a PA6 monomer was observed to be between 54% and 62%. Similarly, Kamimura and Yamamoto [549] employed DMAP and various IL solvents to depolymerize PA6 at a temperature of 300 °C. Among the solvents used, N-methyl-N-propylpiperidinium-bis(trifluoromethanesulfonyl)imide exhibited the most favorable outcome, with 86% caprolactam retrieval.

##### Methanolysis

PET methanolysis offers several benefits, including obtaining N,N-dimethyltryptamine (DMT) of identical quality to virgin DMT, simple purification of DMT, and facile recovery of EG and CH_3_OH. This technique exhibits a relatively high tolerance of contaminants, allowing it to process low-quality feedstocks [519].

In the methanolysis process of PET, CH_3_OH can be utilized in three different states: liquid, superheated vapor, and supercritical fluid. The process of PET methanolysis involves breaking down PET chains by reacting them with CH_3_OH under a high temperature (180–280 °C) and pressure (20–40 atm). Transesterification catalysts (e.g., zinc acetate, cobalt acetate, magnesium acetate, and lead dioxide) are commonly utilized to facilitate the reaction. The yield of DMT typically falls between 80% and 85% [550,551]. Alternatively, superheated CH_3_OH vapor can be used instead of liquid CH_3_OH to react with oligomers or crude BHET (obtained through glycolysis) by passing the CH_3_OH vapor through the glycolysis product. The production of DMT increases with higher CH_3_OH feeding rates and reaches its maximum output at a reaction temperature of 250–260 °C and 3–6 atm reaction pressure. This vapor methanolysis method has proven successful in processing PET waste [552].

More recently, the use of supercritical CH_3_OH at temperatures exceeding 300 °C and pressures above 80 atm has also been applied to PET methanolysis [519,540]. Using supercritical CH_3_OH, Liu et al. [553] were able to depolymerize PET into monomer DMT rapidly and completely. The influence of the MeOH-to-PET mass ratio on the response surface was found to be less significant than that of the reaction temperature and time within the studied range of experimental conditions. The optimal operating conditions for methanolysis were determined to be 112 min, 298 °C, and a mass ratio of 6/1 MeOH/PET. The results showed that PET could be quickly and completely decomposed to its monomer DMT under supercritical CH_3_OH conditions. The degree of depolymerization and selectivity for DMT increased with higher CH_3_OH/PET ratios and reaction temperatures. The experimental conditions based on the optimal values led to a high DMT yield of 99.8%, which was in good agreement with the predicted value.

Numerous catalysts have been employed to hasten PET depolymerization, and their effectiveness with regard to the depolymerization rate, environmental impact, by-products, and process expenses has been evaluated. Ongoing research is exploring a range of catalysts for this purpose. For instance, Jiang et al. [521] employed a poly(IL))-Zn^2+^ (PIL) catalyst to carry out the methanolysis of PET in an efficient and eco-friendly manner. The findings suggest that the reaction temperature is a crucial aspect that affects the process. Under conditions in which the CH_3_OH to PET mass ratio was 3:1 and the quantity of PIL-Zn^2+^ was 2% of the PET mass, complete PET conversion and 89% DMT yield were attainable through methanolysis at 170 °C for 60 min. The PIL-Zn^2+^ catalyst was reused six times without significant performance degradation, implying that poly(IL) may replace conventional compounds in the industrial application of PET catalytic methanolysis.

Tang et al. [554] utilized MgO/crystalline sodium Y (NaY) catalysts to perform PET methanolysis. The 21%MgO/NaY catalyst displayed notable catalytic activity in this process. For a CH_3_OH-to-PET mass ratio of 6, a catalyst dosage of 4 wt%, a temperature of 200 °C, and a reaction time of 30 min, the yield of DMT and the conversion of PET reached 91% and 99%, respectively. The excellent catalytic performance of the 21%MgO/NaY catalyst was attributed to its robust basic sites, large pore size, and high specific surface area. Furthermore, the catalyst alkalinity was found to positively affect PET conversion and DMT yield. Remarkably, the catalyst retained its high catalytic activity even after undergoing six repeated cycles. These results demonstrate that the 21%MgO/NaY catalyst has numerous benefits, such as high efficacy, simple recovery, reusability, and robustness. After regeneration, the catalyst’s activity returned to the level of activity of the unused catalyst. Therefore, utilizing 21%MgO/NaY as a catalyst for PET methanolysis is a promising approach for chemical recycling of PET.

##### Aminolysis

PET aminolysis involves the recycling of PET by reacting it with various primary amine solutions, including methylamine, allylamine, ethanolamine, ethylamine, and hydrazine, in aqueous form (Figure 23d). This reaction leads to the formation of the diamides of TPA and EG.

Amines, being organic bases, cause faster depolymerization compared to alcohols. Catalysts and pre-catalysts (e.g., ILs) utilized in other solvolysis procedures can also exhibit effectiveness in this context. However, this process has not yet been upscaled to an industrial scale, which makes it less feasible to use economically. The temperature range for this process is between 20 °C and 100 °C. Meanwhile, using various types of amines in the aminolysis process can lead to the production of other products, such as a hydrogel adsorbent [555] or a high-temperature cross-linking agent for unsaturated polyesters [556].

Notably, using deep eutectic solvents synthesized with choline chloride.x ZnCl_2_ and choline chloride.2 CH_4_N_2_O as catalysts, Musale and Shukla [557] employed aminolysis by ethanolamine and diethanolamine to depolymerize PET waste. Reaction parameters, including the time of aminolysis, the catalyst concentration, and the PET/amine ratio, were optimized. The pure products obtained were TPA, N^1^,N^1^,N^4^,N^4^-tetrakis(2-hydroxyethyl)-terephthalamide, and bis(2-hydroxyethylene) terephthalamide (BHETA), which were obtained in yields of 83, 82, and 95%, respectively.

##### Ammonolysis

The degradation of PET through ammonolysis involves the attack of anhydrous NH_3_ on PET in an EG medium at temperatures between 70 °C and 180 °C under pressure, producing terephthalamide and EG. EG is used in the reaction atmosphere to facilitate heat and reaction exchanges and acts as a partial PET solvent. The process can lead to a high-purity product with a yield of about 90% [558].

Ammonolysis, which involves the use of NH_3_ to depolymerize PET, has not been as extensively researched as other chemical recycling methods. This is because the profitability of the process depends on the economic potential of the resulting degradation products [559].

Furthermore, ammonolysis is a slower process than aminolysis and usually necessitates the use of a catalyst to increase the depolymerization rate. Catalysts, such as zinc acetate or ammonium quaternary salt, can reduce the reaction time and yield a terephthalamide that can be used as an epoxy hardener or intermediate in other applications [539].

##### Hydrogenolysis

In terms of a circular economy, heterogeneous catalysts that are durable and recyclable are better suited for the hydrogenolysis-based recycling of PET [530,531,560]. Unlike reductive depolymerization, which is a hydrogenative recycling type, hydrogenolysis does not require expensive high-pressure equipment. This feature makes it a safer and more convenient option.

For example, Kratish et al. [561] applied a heterogeneous carbon/molybdenum dioxide catalyst for PET hydrogenolysis. This catalyst exhibited remarkable selectivity because its molybdenum (Mo) sites could selectively activate and cleave ester groups. However, it had low efficiency in PET hydrogenolysis due to its poor ability to activate H_2_. More recently, Ye et al. [562] employed ruthenium (Ru)/TiO_2_ to hydrogenolyze the C–C and C–O bonds of PET, converting it into a series of aromatics. Ru/TiO_2_ possesses an exceptional H_2_ activation capacity, enabling it to hydrogenolyze PET. The most efficient catalyst for the conversion of PET to BTX (77%) was the one with a Ru size of ~1.1 nm and a coordination number of ca. 5.0, which had dominant edge/corner sites. Larger-sized Ru catalysts produced more ring hydrogenation and subsequent ring-opening products.

Zeolitic imidazole frameworks (ZIFs) have also shown potential as a support for metal catalysts in recent years. Multimetallic ZIFs and their derivatives may exhibit superior catalytic performance compared to their monometallic counterparts, and they have been shown to be an effective method for creating bimetallic and multimetallic catalysts.

Note that under solvent-free and atmospheric H_2_ pressure conditions, Wu et al. [530] developed a new and effective heterogeneous bimetallic catalyst (CoMo@NC) for PET hydrogenolysis. The catalyst was prepared through pyrolysis of the Mo@ZIF-CoZn precatalyst in a special procedure. In the optimum range of process parameters (catalyst/PET: 0.3, H_2_: 1 atm, 260 °C, 10 h), TPA was obtained with a high yield (91%). This catalytic approach offers numerous benefits, such as high monomer yields, the absence of a need for reagents or solvents, the use of atmospheric H_2_, low cost, and being a reusable catalyst. The Mo sites in the bimetallic catalyst primarily and effectively catalyze β-scission, while the Co sites activate H_2_ and catalyze the hydrogenolysis process. The coordination between the two metals substantially enhances catalytic efficiency.

Kratish and Marks [531] applied a combination of homogeneous (hafnium trifluoromethanesulfonate [Hf-(OTf)_4_]) and heterogeneous (palladium on carbon [Pd/C]) catalysts as a tandem catalytic system to catalyze selective PET hydrogenolysis without the need for solvents. Under a 1 atm pressure of H_2_ and optimum ranges of parameters (PET/Hf/Pd: 400/6/1, 180 °C, 24 h), PET hydrogenolysis resulted in high yields and selective formation of the corresponding monomers (TPA).

Recently, Kumar et al. [563] reported the first experience of hydrogenative depolymerization of conventional polyamide using a green and sustainable approach based on hydrogenation in the presence of a Ru pincer catalyst at 150 °C and 70 bar H_2_. They also demonstrated the hydrogenation of PU to produce diol, diamine, and CH_3_OH using the same catalytic conditions.

The hydrogenation process is definitely a promising process and has high potential, but the long reaction time is still challenging. It is necessary to shorten the reaction time for the recycling process to become economically competitive. Using an extruder to quickly melt the plastic, increasing the H_2_ pressure, and using more active catalysts can be effective in this field.

##### Reductive Depolymerization

Reductive depolymerization has emerged as a promising alternative approach for the utilization of polycondensation plastics in recent years. It has gained popularity because it enables plastic waste to be converted into value-added products that cannot be obtained using other recycling techniques. Catalysts play a crucial role in this process and must possess high activity, low cost, and stability in the presence of air, moisture, organic compounds, and metallic contaminants [564].

Silanes have been employed as reducing agents in the reduction of PET waste into smaller units in the presence of various organic and organometallic catalysts. PET waste was subjected to reductive depolymerization using B(C_6_F_5_)_3_ (2 mol%) and Et_3_SiH (4.3 equiv.) at ambient temperature, which resulted in the formation of two disilylethers: BDM(1,4-benzenedimethanol)-Si and EG-Si, with yields of 85% and 72%, respectively. Upon hydrolysis of these disilylethers using tetrabutylammonium fluoride trihydrate (TBAF·3H_2_O), 1,4-benzenedimethanol and EG were obtained quantitatively (Figure 23f) [529].

**Figure 24:**
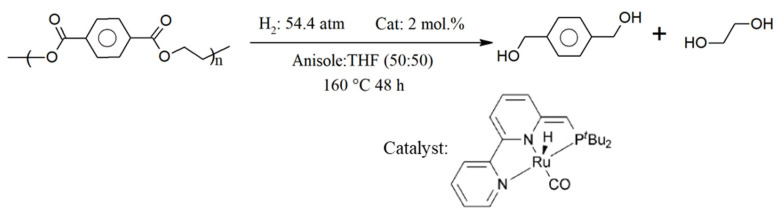
Catalytic hydrogenation of PET via Ru(II) PNN pincer complexes [528].

Different organometallic catalysts have been employed to develop various instances of hydrogenative depolymerization of PET waste. Krall et al. [528] applied Ru(II) PNN pincer complexes (Figure 24) in a controlled catalytic hydrogenation depolymerization technique to depolymerize PET. To overcome the low solubility of PET, a mixture of anisole and tetrahydrofuran (THF) (50:50) was utilized to hydrogenate this polymer. A Ru complex was considered a catalyst at a concentration of 2 mol%, along with 54.4 atm of H_2_ at 160 °C for a duration of 48 h. This process resulted in outstanding conversion rates, yielding 1,4-benzenedimethanol and EG.

**Figure 25:**
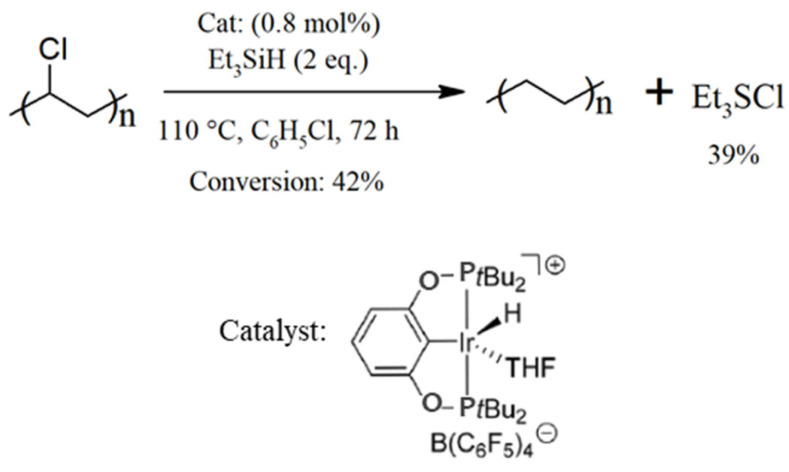
Reductive dechlorination of PVC to PE with the iridium catalyst/Et_3_SiH [565].

The method of regeneration is applicable to various types of plastics, including PVC, and allows for dechlorination, as presented in Figure 25. Monsigny et al. [565] specifically applied an iridium catalyst for the dechlorination of PVC at 110 °C for 72 h, resulting in a conversion rate of approximately 42%. Nevertheless, challenges such as insufficient monomer retrieval, purification complexities, stringent conditions, and the need for hazardous solvents and degradation agents impede the implementation of reductive depolymerization.

Furthermore, this approach frequently necessitates high H_2_ pressures, problematic organic solvents, and additives. In addition, Ru-based homogeneous catalysts are air-sensitive, costly, and challenging to separate and reuse [528].

Polyamides can also be depolymerized using reductive depolymerization, which has the benefit of not generating stoichiometric waste, as with traditional reducing agents. In this field, proficient Ru pincer catalysts have been discovered to hydrogenate amides by cleaving C–N bonds, producing amines and alcohols [566]. Other homogeneous catalysts based on RU, Fe, and Mn have also been reported for this purpose.

## 4. Technology Assessment of Textile Fiber Recycling

Assessing the recycling of textile fibers, whether single or blended, presents numerous challenges. The suitable recycling method varies based on the fiber type, chemical structure, and purity, among other factors [60]. In the initial stage, mechanical recycling is preferred after reuse whenever feasible, particularly in cases of high purity [289,567]. Second, alternative methods, such as dissolution, are often applicable to natural polymers. However, when it comes to synthetic plastics, dissolution typically demands significant energy and solvent usage [194,287,359,365]. If other methods are not viable, chemical recycling techniques, such as pyrolysis for polyolefins and solvolysis for fibers (e.g., polyester and polyamide), can be employed [441,499,538]. For blended fibers, such as those combining polyester and natural fibers, a combined dissolution-solvolysis approach can effectively dissolve natural fibers and facilitate the depolymerization of polyester fibers [536]. 

Table 8 provides an overview of the various methods utilized in the industry to recycle different types of fibers.

## 5. Life Cycle Assessment

As explained above, nowadays, the end-of-life treatment of textiles, both synthetic and natural, mainly involves disposal in landfills and incineration, with a small proportion being recycled using conventional methods, such as mechanical and chemical recycling [569]. Moreover, the most widespread mechanical recycling option often requires downcycling because the loss of textile quality is inevitable. The increase in textile waste is additionally a global problem driven by fast-changing trends, increasing population numbers, and rising purchasing power, particularly in developing countries.

Complementary to the further development of mechanical recycling methods, chemical recycling is thus likely necessary to close the recycling loop [542]. However, the energy intensity of certain processes, as well as reactant consumption, raises the question of whether such a recycling pathway has a lower impact than state-of-the-art processes. An additional question is how to select a preferable technology from among many concepts. In this context, LCA has become a standard [570] method for estimating the overall environmental footprint of a process.

Shen et al. [571], for instance, focused on the chemical recycling of PET through methanolysis to produce DMT and EG. This reaction occurred in the presence of catalysts, such as Zn, Co, or Mg acetates or lead dioxide, at a pressure of 2–4 MPa and a temperature of 180–280 °C. DMT was recovered from the reaction mix through precipitation, centrifugation, and crystallization. The recycled polymer was then converted into fiber via the spinning and finishing processes. The authors considered cradle-to-gate LCA and used a cut-off approach to define the cradle of the recycled polymer as the collection and transportation of PET waste. Three cases—low, high, and average recycled PET—were considered. The low case assumes zero PET loss and 100% recovery of EG, the high case assumes 10% PET loss and no EG recovery, and the average case assumes 5% PET loss and 50% EG recovery. The net CH_3_OH input was assumed to be zero for the low case, 10% for the high case, and 5% for the average case. The resulting carbon footprint, estimated with the global warming potential (GWP) 100a model [572], is 2.71, 3.08, and 3.44 kg of CO_2_ equivalent per kg of treated PET, respectively.

Ügdüler et al. [542] applied a two-step aqueous alkaline hydrolysis approach for PET recycling. Mass and energy balance simulations were conducted using Aspen Plus software, while LCA was performed using open LCA. The hydrolysis process was carried out under mild conditions, yielding approximately 95% of the PET monomers, EG, and TPA in less than 20 min. The purity of these monomers was confirmed through various characterization techniques. The study revealed that the degradation rate is inversely proportional to the particle size and that the decomposition yield is affected by factors such as the thickness and presence of multilayers in PET samples. The researchers performed LCA under system boundaries similar to those in a previous work [571]. Various solid feed/alkaline solution (S/L) ratios were tested, and the results revealed that keeping the S/L ratio at a minimum of 0.04 g mL^−1^ decreases the process’s carbon footprint below virgin production levels.

The LCA results demonstrated that the hydrolysis process has the potential to yield environmental savings in terms of carbon emissions compared to incineration, a common practice for complex PET waste streams, such as PET trays and films. The energy consumption during solvent and product recovery constitutes the largest share (>55%) of GHG emissions (Table 9). The study concluded that industrial optimization of the hydrolysis process can result in significant environmental benefits in terms of reduced carbon emissions. Furthermore, the process has the potential to recycle other polymers from multilayer structures, such as PE, from PET trays. Moreover, the successful removal of various colors, including carbon black, from the hydrolysate demonstrates the versatility of this method, paving the way for increased recycling rates of PET trays and films, which have historically been challenging to recycle due to their complex compositions and poor collection rates.

In the case of natural fibers, such as cotton, open-loop chemical recycling is prevalent over closed-loop recycling. In this framework, Paunonen et al. [573] investigated the cellulose carbamate (CCA) process as a compelling alternative for producing man-made fibers. It is compatible with traditional viscose spinning processes, allowing existing mills to easily adopt it. In the CCA process, dissolving pulp is combined with CH_4_N_2_O at high temperatures, causing CH_4_N_2_O to break down into isocyanic acid (HNCO) and NH_3_. The highly reactive HNCO then forms carbamates with cellulose hydroxyl groups. These newly created carbamate groups enhance the solubility of cellulose in aqueous NaOH, enabling the dissolved CCA to be transformed into textile fibers within an acidic coagulation bath. The authors assume that the properties of such fibers are comparable to those of traditional viscose fibers; hence, the cut-off cradle-to-gate system boundary was selected.

In greater detail, it includes sorting discarded cotton textiles, transportation, and the CCA process itself, from shredding the cotton textile to CCA fiber spinning and bailing. Two scenarios were considered: one with a standalone CCA factory and one integrated with a pulp mill setup. The standalone facility has shown a significantly higher carbon footprint than typical viscose production from wood pulp. In contrast, the integrated scenario has proven to be able to reduce GHG by up to 40% below the reference value. Besides GHG reduction, the CCA process consumes significantly less water, only 31 L per kg of produced fiber (integrated case), whereas a conventional viscose factory consumes 445 L, and cotton growing and processing consume up to 4342 L.

**Figure 26:**
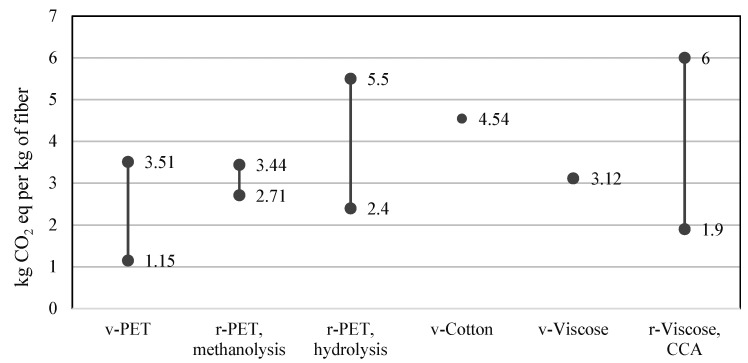
Carbon footprint ranges of state-of-the-art virgin and recycled fiber production. v-PET = virgin PET; r-PET = recycled PET via methanolysis pathway [571]; r-PET, hydrolysis = PET recycled via the alkaline hydrolysis pathway [542]; v-Cotton and v-Viscose = virgin fibers; r-Viscose, CCA = cellulose carbamate fiber derived from waste cotton [573].

To fairly compare the described processes, the cradle-to-gate carbon footprints of recycled fibers have also been benchmarked against corresponding virgin fiber production with state-of-the-art processes. The impacts of the latter have been quantified, for instance, with the SimaPro package and the ecoinvent 3.8 database, using the GWP 100 impact assessment method. Interestingly, this baseline approach (Figure 26) is consistent with the values disseminated in the literature [573].

Since the typical end-of-life scenario for polymer waste, particularly in Belgium, is incineration [574], it is necessary to include its impact in the comparison because the benefit of recycling is a substitute process for both virgin material production and waste end-of-life treatment. Hence, the baseline carbon footprint is expressed in Figure 26 as a range, in which the lower limit is the contribution of the production process alone, and the upper boundary includes waste treatment as well.

## 6. Eco-Design

To address the environmental challenges associated with textile waste, it is crucial not only to develop commercially feasible collection, sorting, pretreatment, and recycling processes but also to change how textile products are designed in the first place. By implementing certain design principles, the need for recycling as an end-of-pipe solution for textile waste can be reduced, and the efficiency of end-of-life processes can be enhanced. These principles, commonly called “eco-design”, focus on making textiles easier to recycle, extending product lifecycles, and reducing waste at the source. “Design for recycling” is a subset of eco-design concerned with facilitating recycling processes [575].

The first eco-design principle is designing for longevity, which emphasizes using high-quality fibers, reinforcing weak points (e.g., seams), and incorporating repairable, replaceable, or modular features (e.g., attached via zippers), thus creating durable and easily customizable products. For garments specifically, it is also important to both design the aesthetics to be timeless rather than what is encouraged by fast fashion trends [576] and to encourage modularity and adaptability, such as by allowing consumers to change their clothing by attaching or detaching sleeves, hoods, etc. This approach extends the lifespan of textile products, thus reducing waste generation, the overall demand for replacement products, and the associated environmental impact. Extending a garment’s life by just three months, for example, has been found to reduce its carbon, water, and waste footprints by 5–10% each [577].

A second eco-design principle is related to material selection, as the environmental impact of a virgin product and its recyclability are directly tied to the material. For instance, using renewable, biodegradable, and recycled materials ensures that textiles have a lower environmental impact and are likely to be recovered at the end of their lives. Organic cotton, Tencel^TM^, and recycled polyester are favored for their lower energy requirements and recyclability. Purposefully using mono-materials (textiles made from a single type of fiber) is also crucial because it simplifies the recycling process, removing the need for separation and sorting of mixed textiles, which is challenging and resource-intensive. Mono-materials thus allow for streamlined recycling processes that yield higher-quality secondary raw materials [578].

A third eco-design principle concerns additives in textile products. While products without any additives would be the easiest to recycle, they are necessary for the product’s function (e.g., dyes in clothing) and may increase its lifespan. Hence, this principle may contradict the design-for-longevity principle. For example, applying durable press finishes or hydrophobic finishes to garments may reduce their need for care and maintenance, thus reducing the environmental impact of such processes (e.g., laundering) and extending the garments’ lifespans (washing clothes is detrimental to their lifespans). However, many of these finishing compounds (e.g., fluorocarbon-based hydrophobic finishes) interfere with recycling processes and are difficult to remove, thus remaining in the recycled product and lowering its quality. Colorants have a less ambiguous nature and should be chosen to be easily removable from textile waste with a common treatment process [262].

A fourth eco-design principle is design for disassembly. Garments incorporating multiple components, such as buttons, zippers, and linings, can slow the recycling process down. Designing textiles with fewer fasteners or using fasteners that can be easily removed or recycled alongside the fabric significantly improves recyclability [312]. A recent example is the manufacture of textile articles with special stitching threads that can be disintegrated at the end of the product’s life cycle without damaging the rest of the product. This means no cutting occurs, so the textile pieces maintain their maximum size (i.e., allowing high-quality mechanical recycling). The downsides are that the system is limited to stitched goods and that identifying the thread at the end of an article’s life cycle may be challenging [194,269]. To date, two companies offer these yarns: Resortecs and Wear2. The Resortecs threads can be broken down by applying heat (between 150 °C and 190 °C) [579], while the Wear2 threads contain metallic particles and can thus be disintegrated by applying microwaves [580].

Before disassembly can take place, textile waste needs to be collected and sorted. Hence, a fifth eco-design principle is design for identification. If a waste product can be fully identified with a simple system at the end of its life cycle, that information can be used to sort it into the correct waste category without any external analysis. Concepts such as RFID tags and photonic crystals, are excellent examples of this principle [208,209].

A final example of a design principle concerns efficient production techniques. Reducing the amount of water, energy, and chemicals required during manufacturing can lead to significant environmental savings. For example, techniques such as cold dyeing and digital printing reduce energy and water use compared to traditional methods [581].

While not a principle of product design, it is important to note that exploring new business models that can disrupt traditional linear consumption patterns is vital to the industry’s sustainability. Companies can implement take-back schemes, such as collecting and repurposing old products or adopting rental and leasing services, particularly for items typically used infrequently, such as formal wear. These models not only reduce waste but also create new revenue streams and customer engagement opportunities [582,583].

Addressing the aforementioned eco-design principles and business models collectively can drive significant progress toward a more sustainable textile industry. A holistic, integrated approach mitigates environmental impacts and fosters a more responsible and resilient industry capable of adapting to the growing demand for sustainability from consumers and regulators alike.

Several manufacturers are leading the integration of recycling processes in textile production. REPREVE^®^ produces performance fibers from recycled plastic bottles and textile waste, while Evrnu^®^ develops engineered fibers from discarded clothing. Vivify Textiles specializes in sustainable fabrics made from recycled materials like PET plastic and textile waste. Additionally, Pure Waste Textiles creates fabrics using 100% recycled materials sourced from garment factory waste. These companies exemplify the growing trend toward sustainable practices in the textile industry [584,585].

## 7. Conclusions and Perspectives

This review has underscored the escalating environmental burden of textile waste, which is rapidly growing in volume, diversity, and complexity. To address this, a shift toward more efficient and sustainable end-of-life technologies is essential. In this work we have explored both existing and emerging methods for textile waste collection, sorting, and recycling, identifying key advancements while highlighting areas that require further development.

Automated collection systems, powered by smart tags and sensor networks, are emerging as a game-changer in textile waste management. These systems improve efficiency in tracking and sorting, yet they require refinement to reduce costs and ensure widespread adoption. The success of these systems will hinge on enhanced infrastructure, active participation from consumers and producers, and supportive policies such as extended producer responsibility (EPR) legislation.

For sorting, advanced technologies like near-infrared (NIR) and hyperspectral imaging (HSI) stand at the forefront, enabling precise fiber separation based on chemical composition. However, challenges remain, particularly with complex or multilayered textiles that these technologies struggle to analyze. Future development should focus on tackling the increasing complexity of blended fabrics and integrating hybrid sorting technologies that combine spectroscopic methods with AI-based detection.

Pretreatment is another critical area, especially removing non-textile components, colorants, and finishing compounds, which interfere with recycling processes. While automated disassembly technologies are promising, they are still in the early stages and need significant speed, accuracy, and cost-efficiency improvements to become viable. Similarly, scalable and cost-effective methods for removing colorants and finishes are urgently needed to improve the quality of recycled fibers.

Recycling technologies are broadly categorized into mechanical and chemical methods. Mechanical recycling, while cost-effective and environmentally favorable, is limited by its inability to maintain fiber quality over repeated cycles, especially for blended fabrics. Polymer recycling, which involves melting or dissolving waste polymers, offers higher-quality outputs but is more resource-intensive. Chemical recycling methods like solvolysis and pyrolysis hold the potential for producing virgin-quality monomers, particularly for synthetic fibers, while biological recycling shows promise for natural fibers. However, these methods face significant scalability challenges due to high energy consumption and operational costs.

Looking ahead, the future of textile recycling depends on the integration of advanced sorting technologies, scalable pretreatment solutions, and efficient recycling processes. Innovations such as automated disassembly, engineered enzymes for biolysis, and improved chemical recycling techniques will be pivotal in closing the material loop. Reducing energy inputs, optimizing processes, and developing solvent recovery systems will be key to making chemical recycling more viable on a larger scale.

Beyond technological advancements, collaboration between policymakers, industry leaders, and researchers is crucial to driving change. Policies that incentivize eco-design and encourage EPR will be vital, as will industry cooperation to standardize recycling processes and adopt innovative technologies. Consumer engagement will also play a central role, both in reducing waste generation and participating in textile recycling efforts.

In conclusion, while significant strides have been made in end-of-life technologies for textiles, much work remains to overcome the current challenges. By combining advanced sorting, pretreatment, and recycling technologies with strong policy support and industry collaboration, the textile sector can move toward a sustainable, closed-loop future—one that minimizes waste, maximizes material recovery, and significantly reduces its environmental footprint.

## Figures and Tables

**Table 3:** Overview of physical recycling (dissolution) of textile wastes.

Fiber	Solvent	Feed/Solvent	Process Conditions	Complementary Processes and Pretreatments	Products	Advantages	Ref.
Blends of polyester and cotton	AMIMCl ^1^	2–10 wt%	80 °C, 6 h	-	Cotton, polyester	The IL was recovered. Cotton can be regenerated using water as the coagulated solvent. Selective dissolution of the cotton component using ionic liquid. Fully recovered and almost-pure polyester and cotton.	[376]
An orange 50:50 polyester–cotton blend and a blue 40:60 polyester–viscose blend	NMMO ^2^	18 wt%	120 °C, 2 h	Two-day enzymatic hydrolysis (yield: 85%) and one-day fermentation (yield: 89%) of the man-made cotton and viscose	Polyester, cotton, viscose, ethanol, biogas	The polyesters were purified as fibers after the NMMO treatments. Up to 95% of the cellulose fibers were regenerated and collected. It is possible to recover the solvent efficiently. This process might be economically feasible. Possible NMMO degradation and cellulose oxidation, and requires antioxidants for stabilization.	[377]
Waste polyester-cotton jeans	AmimCl ^3^	4 wt%	80 °C, 3 h	-	PET, cotton	Cotton components can be directly dissolved and regenerated. The recovered PET fibers can be recycled and reused in textiles. The resulting cellulose solution dope can be used for dry-jet wet spinning to obtain lyocell-type fibers.	[378]
Dyed PET, cotton, PA6,6 fibers	DMSO, ethylene carbonate, glycerol, tetramethylurea	20 wt%	80–100 °C, 0.5 h	-	Decolorized fibers	One hundred percent of the disperse dyes, acid dyes, and direct dyes were separated from PET, PA6,6, and cotton, respectively. Dye removal did not change the structures or dyeability of the dyes. The molecular weights of the polymers after dye removal were almost the same.	[362]
Colored waste garment	[Bmim]OAc/DMSO	Max. 18 wt%	80 °C, 0.5 h	NaOH hydrolysis, wet spinning	Decolorized fibers	The solubility of a solvent system is also strongly influenced by the molecular weight of the cellulose. The use of a binary solvent containing a low IL amount reduces the drawbacks (e.g., high dope viscosity). The binary solvent reduces the cost of the solvent by 77%. The solvent can be recovered and reused via distillation.	[349]
Blue waste jeans (80/20 cotton/polyester)	DMCHA ^4^	1/40–1/120 g·mL^−1^	50 °C, full dissolution	Dye leaching (HNO_3_)	Decolorized cotton fiber and polyester with high purity	A green dissolution process was employed to dissolve polyester. Polyester was extracted from the solution by changing the hydrophilicity of the solvent. The recycling rate of the technology was >96%. The economic returns are up to $1629/ton of waste, and the carbon footprint is reduced by 1440 kg of CO_2_-eq/t of waste.	[284]

^1^ 1-allyl-3-methylimidazolium chloride; ^2^ N-methylmorpholine-N-oxide; ^3^ 1-allyl-3-methylimidazolium; ^4^ N,N-dimethylcyclohexylamine.

**Table 7:** An overview of common solvolysis processes.

Polymer	Method	Agent	Catalyst	Catalyst: Polymer	T (°C)	t (min)	Reactor	P (atm)	Yield %	Ref.
PET	Glycolysis	EG	Di-n-butylamine	1:4 (mol%)	160	90	Three-necked flask	1	76.8 BHET	[508]
			Fe_3_O_4_-boosted ^1^ MWCNT	1:19 (wt%)	190	120	Stainless steel cylinder	1	100 BHET	[509]
			^2^ TBD:^3^ MSA	1:99 (wt%)	180	120	Schlenck flask	1	91.0 BHET	[510]
			^4^ POM:^5^ WZn_3_	1:2 (wt%)	190	40	Three-necked flask		84.5 BHET	[511]
			(Dimim)[FeCl_4_]	1:4 (wt%)	170	120	Three-necked flask	1	99.0 BHET	[512]
			^6^ [Ch][For]	1:19 (wt%)	180	180	Three-necked flask	1	84.5 BHET	[513]
			^7^ [Ch][OAC]	1:19 (wt%)	180	240			85.2 BHET	
			MnO_2_/holey GO nanosheets	1:10,000 (wt%)	200	10	Three-necked flask		100 BHET	[514]
			Acetaminde/ZnCl_2_	1:250 (wt%)	195	25	Three-necked flask	1	83.2 BHET	[515]
			DES@Zif-8							
			Orange peel ash	1:10 (wt%)	190	90	Two-necked flask	1	79.0 BHET	[516]
			Fe_3_O_4_@SiO_2_@	3:20 (wt%)	180	1440	Round bottom flask		100 BHET	[517]
			(mim)[FeCl_4_]							
			RZnO	1:100 (wt%)	196	120	Three-necked flask		50.0 BHET	[518]
			Co/RZnO	1:100 (wt%)	196	120			80.0 BHET	
	Methanolysis	MeOH	BLA	1:5 (wt%)	200	120	Autoclave	1	78.0 DMT	[519]
			Modified ZnO	7:1000 (wt%)	170	15	Autoclave		95.0 DMT	[520]
			PIL-Zn^2+^	1:50 (wt%)	170	60	Bottle reactor		90.3 DMT	[521]
		MeOH/CH_2_Cl_2_	K_2_CO_3_	1:5 (mol%)	25	1440	Round bottom flask	1	93.1 DMT	[501]
			CH_3_OK	1:5 (mol%)	25	1440			81.9 DMT	
	Hydrolysis	H_2_O	Marine water (metallic ions)	7:20 (wt%)	205	120	Stirred reactor	32.5	96.0 TPA	[522]
			NaHCO_3_ + KHCO_3_	8:20 (wt%)	195	120	Stirred reactor	34.5	95.7 TPA	
			-	-	230	30	Microwave	~29	77.0 TPA	[523]
			-	-	200	30	Microwave		12.0 TPA	
			H^+^@ZSM-5	1:2 (wt%)	230	30	Microwave		98.5 TPA	
			H^+^@ZSM-5	1:2 (wt%)	200	30	Microwave		87.0 TPA	
	Aminolysis	Hexyl-amine	-	-	180	30	Microwave	1	64.0 ^8^ DHTA	[524]
		Ethanol-amine	-	-	200	10	Microwave		91.0 ^9^ BHTA	
		Furfuryl-amine	-	-	200	60	Microwave		82.0 ^10^ BFTA	
		Allyl-amine	-	-	280	15	Microwave		61.0 ^11^ DAA	
		Isophorenediamine	Zn(OAc)_2_	1:10 (wt%)	200	240	Four-necked flask		90.0 TPA	[525]
		Ethanol-amine	^12^ mHpb	5:32 (wt%)	190	3	Three-necked flask	1	100 ^13^ BHETA	[526]
		Ethanol-amine	TBD	7:192 (wt%)	120	120	Schlenk flask	1	93.0 TPA	[527]
	Reductive depolymerization	H_2_	Ruthenium(II) PNN pincer	2:100 (mol%)	160	2880	Schlenk tube	54.4	>99 TPA, EG	[528]
		Et_3_SiH	B(C_6_F_5_)_3_	2:100 (mol%)	25	180	Stirred reactor		91.0 BDM-SI	[529]
	Hydrogenolysis	H_2_	CoMo@NC	3:10 (wt%)	260	1200	Sealed tube	1	91.0 TPA	[530]
		H_2_	Hf-(OTf)_4_: Pd/C	Pd/Hf/PET: 1:6:400	180	1440	Schlenk flask	1	95–98 TPA	[531]
PA6		^14^ IL	-	-	280	60	Microwave	1	0 caprolactam	[532]
		IL	-	-	300	60	Microwave		36 caprolactam	
		IL	^15^ DMAP	1:10 (wt%)	300	60	Microwave		55 caprolactam	
		IL	DMAP	1:10 (wt%)	300	30	Microwave		24 caprolactam	
		IL	DMAP	1:10 (wt%)	310	60	Microwave		54 caprolactam	
	Acid hydrolysis	HCl	-	-	80	120	Round bottom flask	1	72.2 ^16^ DBHMD	[533]

^1^ MWCNT, multiwall carbon nanotube; ^2^ TBD, 1,5,7-triazabicyclo[4.4.0]dec-5-ene; ^3^ MSA, methanesulfonic acid; ^4^ POM, polyoxometalates; ^5^ WZn_3_, Na_12_ [WZn_3_(H_2_O)_2_(ZnW_9_O_34_)_2_]; ^6^ [Ch][For], choline formate; ^7^ [Ch][OAC], choline acetate; ^8^ DHTA, dihexylterephthalamide; ^9^ BHTA, bis(2-hydroxyethyl)terephthalamide; ^10^ BFTA, bis(furan-2-ylmethyl)terephthalamide; ^11^ DAA, diallylterephthalamide; ^12^ mHpb, methyl 4-(2,3,4,6,7,8-hexahydro-1H-pyrimido [1,2-a]pyrimidine-1-carbonyl) benzoate; ^13^ BHETA, bis(2-hydroxyethyl) terephthalamide; ^14^ IL, hydrophilic ionic liquids [emim][BF_4_]; ^15^ DMAP, N,N-dimethylaminopyridine; ^16^ DBHMD, dibenzoyl derivative of hexamethylenediamine.

**Table 8:** Technology assessment of closed-material-loop end-of-life options for different fiber types.

	Fiber Type		
Closed-loop end-of-life option	Natural fibers	Synthetic fibers	Blends elastane + cotton/polyamide/PET
Reuse	If textiles or fibers are reusable, they are a more suitable option than the other alternatives [189].		
Fiber recycling	Good option, but shortens fiber length [60,270,310]	Good option, but needs high purity and shortens fiber length [289,567,568]	Elastane makes this incompatible [312,315].
Polymer recycling (re-extrusion) via melting	-	Good option [322,325,326]	-
Polymer recycling (re-extrusion) via dissolution	Good option [287,365]	Good option for removing additives and contaminants in polymers, but high energy consumption; under research [194,359,362]	Good option for separating cotton [40,364]
Monomer recycling via solvolysis	-	Good option for pure PET and polyamide [499,500,515,532,533]	Selectivity elastane vs. PET needed (complicated but possible) [59,536,538]
Monomer recycling via pyrolysis	-	Good option for polyolefins [434,437,438,441]	-
Monomer recycling via biological processes	Good option [419,421,426,428]	-	-
Monomer recycling via gasification	When options are limited due to low purity and high degree of contamination, gasification can be a suitable alternative [467,469,470,471].		
Combination of dissolution + solvolysis	-	-	Good option [289,536]
Combination of biological processes + solvolysis	-	-	Good option [66,536]

**Table 9:** Results of the LCA work presented in Ügdüler et al. [542].

S/L Ratio	Estimated GHG Emissions [kg CO_2_ eq/kg of Recycled PET]
0.02	5.6
0.03	3.9
0.04	3.0
0.05	2.4

## References

[1] Elsasser V.H. (2005). Textiles: Concepts and Principles.

[2] Horrocks A.R., Anand S.C. (2016). Handbook of Technical Textiles: Technical Textile Applications.

[3] Hasan M.M., Salman M.S., Hasan M.N., Rehan A.I., Awual M.E., Rasee A.I., Waliullah R.M., Hossain M.S., Kubra K.T., Sheikh M.C. (2023). Facial conjugate adsorbent for sustainable Pb(II) ion monitoring and removal from contaminated water. Colloids Surf. A Physicochem. Eng. Asp..

[4] Rehan A.I., Rasee A.I., Awual M.E., Waliullah R.M., Hossain M.S., Kubra K.T., Salman M.S., Hasan M.M., Hasan M.N., Sheikh M.C. (2023). Improving toxic dye removal and remediation using novel nanocomposite fibrous adsorbent. Colloids Surf. A Physicochem. Eng. Asp..

[5] Kubra K.T., Hasan M.M., Hasan M.N., Salman M.S., Khaleque M.A., Sheikh M.C., Rehan A.I., Rasee A.I., Waliullah R.M., Awual M.E. (2023). The heavy lanthanide of Thulium(III) separation and recovery using specific ligand-based facial composite adsorbent. Colloids Surf. A Physicochem. Eng. Asp..

[6] Loccufier E., Geltmeyer J., Daelemans L., D’hooge D.R., De Buysser K., De Clerck K. (2018). Azeotrope Separation: Silica Nanofibrous Membranes for the Separation of Heterogeneous Azeotropes. Adv. Funct. Mater..

[7] Swanckaert B., Geltmeyer J., Rabaey K., De Buysser K., Bonin L., De Clerck K. (2022). A review on ion-exchange nanofiber membranes: Properties, structure and application in electrochemical (waste)water treatment. Sep. Purif. Technol..

[8] Swanckaert B., Loccufier E., Geltmeyer J., Rabaey K., De Buysser K., Bonin L., De Clerck K. (2023). Sulfonated silica-based cation-exchange nanofiber membranes with superior self-cleaning abilities for electrochemical water treatment applications. Sep. Purif. Technol..

[9] Swanckaert B., Vande Velde N., Loccufier E., De Buysser K., Bonin L., De Clerck K. (2023). High capacity, silica-based anion-exchange nanofiber membranes for the selective recovery of lactic acid. Sustain. Mater. Technol..

[10] Van Eygen G., Keuppens S., De Breuck X., Swankaert B., Boura P., Loccufier E., Kosek J., Ramasamy D., Nahra F., Buekenhoudt A. (2025). Comparison of distinctive polymeric membrane structures as support materials for membrane extraction of chiral amines. Sep. Purif. Technol..

[11] Li M., Loccufier E., Geltmeyer J., D’hooge D.R., De Buysser K., De Clerck K. (2024). Playing with Chlorine-Based Post-modification Strategies for Manufacturing Silica Nanofibrous Membranes Acting as Stable Hydrophobic Separation Barriers. Adv. Fiber Mater..

[12] Siliņa L., Dāboliņa I., Lapkovska E. (2024). Sustainable textile industry—Wishful thinking or the new norm: A review. J. Eng. Fibers Fabr..

[13] Estévez S., Mosca Angelucci D., Moreira M.T., Tomei M.C. (2024). Techno-environmental and economic assessment of color removal strategies from textile wastewater. Sci. Total Environ..

[14] Brundtland Report (1987). Report of the World Commission on Environment and Development: Our Common Future.

[15] Böttcher T.P., Empelmann S., Weking J., Hein A., Krcmar H. (2024). Digital sustainable business models: Using digital technology to integrate ecological sustainability into the core of business models. Inf. Syst. J..

[16] Elkington J. (1998). Cannibals with Forks: The Triple Bottom Line of 21st Century Business.

[17] Statista Distribution of Textile Fibers Production Worldwide in 2021, by Type 2021. https://www.statista.com/statistics/1250812/global-fiber-production-share-type/.

[18] OEC (2021). Textiles. https://oec.world/en/profile/hs/textiles?yearSelector1=2021.

[19] (2022). Textile Market (by Raw-Material: Cotton, Chemical, Wool, Silk; by Product: Natural Fibers, Polyesters, Nylon; by Application: Household, Technical, Fashion & Clothing)—Global Industry Analysis, Size, Share, Growth, Trends, Revenue, Regional Outlook 20222030. Vision Research Reports. https://www.visionresearchreports.com/textile-market/39190.

[20] (2022). Facts & Key Figures.

[21] Common Objective Faces and Figures: Who Makes Our Clothes?. https://www.commonobjective.co/article/faces-and-figures-who-makes-our-clothes.

[22] The World Bank (2023). Labor Force, Total. https://data.worldbank.org/indicator/SL.TLF.TOTL.IN.

[23] Grace Annapoorani S., Muthu S.S. (2017). Social Sustainability in Textile Industry. Sustainability in the Textile Industry.

[24] Riba J.-R., Cantero R., Canals T., Puig R. (2020). Circular economy of post-consumer textile waste: Classification through infrared spectroscopy. J. Clean. Prod..

[25] (2022). The Impact of Textile Production and Waste on the Environment (Infographic). European Parliament. https://www.eumonitor.eu/9353000/1/j9vvik7m1c3gyxp/vlf0bgvqy6vx?ctx=vjxzjv7ta8z1.

[26] Abbate S., Centobelli P., Cerchione R., Nadeem S.P., Riccio E. (2024). Sustainability trends and gaps in the textile, apparel and fashion industries. Environ. Dev. Sustain..

[27] (2022). Textiles and the Environment: The Role of Design in Europe’s Circular Economy. European Environment Agency. https://www.eea.europa.eu/publications/textiles-and-the-environment-the/textiles-and-the-environment-the.

[28] (2017). A New Textiles Economy: Redesigning Fashion’s Future.

[29] Beans C. (2023). Can nature inspire sustainable fashion?. Proc. Natl. Acad. Sci. USA.

[30] Napper I.E., Thompson R.C. (2016). Release of synthetic microplastic plastic fibres from domestic washing machines: Effects of fabric type and washing conditions. Mar. Pollut. Bull..

[31] Leal Filho W., Dinis M.A.P., Liakh O., Paço A., Dennis K., Shollo F., Sidsaph H. (2024). Reducing the carbon footprint of the textile sector: An overview of impacts and solutions. Text. Res. J..

[32] Samant L., Pavan M., Goel A., Kaur M., Sadhna K.R., Greeshma S. (2024). Impact of the Textile Industry on Global Climate Change. Climate Action Through Eco-Friendly Textiles.

[33] Remy N., Speelman E., Swartz S. (2016). Style That’s Sustainable: A New Fast-Fashion Formula. McKinsey Sustainability. https://www.mckinsey.com/capabilities/sustainability/our-insights/style-thats-sustainable-a-new-fast-fashion-formula.

[34] Soares B., Ramos M., Martinho G. (2024). Factors to consider for the implementation of a municipal scheme for the separate collection of textile waste. Sustain. Futures.

[35] Kurniawan T.A., Meidiana C., Goh H.H., Zhang D., Othman M.H.D., Aziz F., Anouzla A., Sarangi P.K., Pasaribu B., Ali I. (2024). Unlocking synergies between waste management and climate change mitigation to accelerate decarbonization through circular-economy digitalization in Indonesia. Sustain. Prod. Consum..

[36] Wang S., Salmon S. (2022). Progress toward Circularity of Polyester and Cotton Textiles. Sustain. Chem..

[37] Riba J.-R., Cantero R., Puig R. (2022). Classification of Textile Samples Using Data Fusion Combining Near-and Mid-Infrared Spectral Information. Polymers.

[38] Daelemans L., Van Paepegem W., D’hooge D.R., De Clerck K. (2019). Excellent nanofiber adhesion for hybrid polymer materials with high toughness based on matrix interdiffusion during chemical conversion. Adv. Funct. Mater..

[39] Tian R., Lv Z., Fan Y., Wang T., Sun M., Xu Z. (2024). Qualitative classification of waste garments for textile recycling based on machine vision and attention mechanisms. Waste Manag..

[40] Phan K., Ügdüler S., Harinck L., Denolf R., Roosen M., O’Rourke G., De Vos D., Van Speybroeck V., De Clerck K., De Meester S. (2023). Analysing the potential of the selective dissolution of elastane from mixed fiber textile waste. Resour. Conserv. Recycl..

[41] Delva L., Van Kets K., Kuzmanovic M., Demets R., Hubo S., Mys N., De Meester S., Ragaert K. (2019). Mechanical Recycling of Polymers for Dummies. Capture-Plast. Resour. https://www.ugent.be/ea/match/cpmt/en/research/topics/circular-plastics/mechanicalrecyclingfordummiesv2.pdf.

[42] Bigambo P., Carr C.M., Sumner M., Rigout M. (2021). Investigation into the removal of pigment, sulphur and vat colourants from cotton textiles and implications for waste cellulosic recycling. Color. Technol..

[43] Kamble Z., Behera B.K. (2021). Upcycling textile wastes: Challenges and innovations. Text. Prog..

[44] Huang X., Tan Y., Huang J., Zhu G., Yin R., Tao X., Tian X. (2024). Industrialization of open- and closed-loop waste textile recycling towards sustainability: A review. J. Clean. Prod..

[45] Yang K., Wang M., Wang X., Shan J., Zhang J., Tian G., Yang D., Ma J. (2024). Polyester/Cotton-Blended Textile Waste Fiber Separation and Regeneration via a Green Chemistry Approach. ACS Sustain. Chem. Eng..

[46] Amundarain I., López-Montenegro S., Fulgencio-Medrano L., Leivar J., Iruskieta A., Asueta A., Miguel-Fernández R., Arnaiz S., Pereda-Ayo B. (2024). Improving the Sustainability of Catalytic Glycolysis of Complex PET Waste through Bio-Solvolysis. Polymers.

[47] Yadav A., Yadav P., Bojjagani S., Srivastava J.K., Raj A. (2024). Investigation of the speciation and environmental risk of heavy metals in biochar produced from textile sludge waste by pyrolysis at different temperatures. Chemosphere.

[48] Seifali Abbas-Abadi M., Nekoomanesh Haghighi M., McDonald A.G., Yeganeh H. (2015). Estimation of pyrolysis product of LDPE degradation using different process parameters in a stirred reactor. Polyolefins J..

[49] Zhang G., Chen Z., Chen T., Jiang S., Evrendilek F., Huang S., Tang X., Ding Z., He Y., Xie W. (2024). Energetic, bio-oil, biochar, and ash performances of co-pyrolysis-gasification of textile dyeing sludge and Chinese medicine residues in response to K_2_CO_3_, atmosphere type, blend ratio, and temperature. J. Environ. Sci..

[50] Bengtsson J., Peterson A., Idström A., de la Motte H., Jedvert K. (2022). Chemical Recycling of a Textile Blend from Polyester and Viscose, Part II: Mechanism and Reactivity during Alkaline Hydrolysis of Textile Polyester. Sustainability.

[51] Provin A.P., Cubas A.L.V., Dutra A.R.d.A., Schulte N.K. (2021). Textile industry and environment: Can the use of bacterial cellulose in the manufacture of biotextiles contribute to the sector?. Clean Technol. Environ. Policy.

[52] Juanga-Labayen J.P., Labayen I.V., Yuan Q. (2022). A Review on Textile Recycling Practices and Challenges. Textiles.

[53] Sandberg E., Pal R. (2024). Exploring supply chain capabilities in textile-to-textile recycling—A European interview study. Clean. Logist. Supply Chain.

[54] Pensupa N. (2020). Recycling of end-of-life clothes. Sustainable Technologies for Fashion and Textiles.

[55] Sulakhe V.N. (2022). Introduction to Semisynthetic and Synthetic Fiber Based Composites. Natural and Synthetic Fiber Reinforced Composites: Synthesis, Properties and Applications.

[56] Shabbir M., Naim M. (2019). Introduction to Textiles and the Environment. Textiles and Clothing.

[57] Rajak D.K., Wagh P.H., Linul E. (2022). A review on synthetic fibers for polymer matrix composites: Performance, failure modes and applications. Materials.

[58] McCauley E., Jestratijevic I. (2023). Exploring the Business Case for Textile-to-Textile Recycling Using Post-Consumer Waste in the US: Challenges and Opportunities. Sustainability.

[59] Loo S.-L., Yu E., Hu X. (2023). Tackling critical challenges in textile circularity: A review on strategies for recycling cellulose and polyester from blended fabrics. J. Environ. Chem. Eng..

[60] Damayanti D., Wulandari L.A., Bagaskoro A., Rianjanu A., Wu H.-S. (2021). Possibility Routes for Textile Recycling Technology. Polymers.

[61] Shahid M.A., Hossain M.T., Habib M.A., Islam S., Sharna K., Hossain I., Mortuza Limon M.G. (2024). Prospects and challenges of recycling and reusing post-consumer garments: A review. Clean. Eng. Technol..

[62] Tripathi M., Sharma M., Bala S., Thakur V.K., Singh A., Dashora K., Hart P., Gupta V.K. (2024). Recent technologies for transforming textile waste into value-added products: A review. Curr. Res. Biotechnol..

[63] Iezzi B., Shtein M., Wang T., Rothschild M. (2024). Fiber and Fabric-Integrated Tracing Technologies for Textile Sorting and Recycling. Technology Innovation for the Circular Economy.

[64] Baldia C.M., Armitage R.A. (2023). Archaeological Textiles as Secondary Plant and Animal Products. Handbook of Archaeological Sciences.

[65] Miles D.C., Briston J.H. (1965). Polymer Technology.

[66] Harmsen P., Scheffer M., Bos H. (2021). Textiles for Circular Fashion: The Logic behind Recycling Options. Sustainability.

[67] Textile Exchange (2023). Materials Market Report. https://textileexchange.org/app/uploads/2023/11/Materials-Market-Report-2023.pdf.

[68] Wang K., Wang Q., Zou L., Su Y., Liu K., Li W., Zhang K., Wang H., Song J. (2023). Study on thermal protection and temperature of PMMA plastic optical fiber for concentrated sunlight transmission in daylighting. Sol. Energy.

[69] Al-Furjan M.S.H., Shan L., Shen X., Zarei M.S., Hajmohammad M.H., Kolahchi R. (2022). A review on fabrication techniques and tensile properties of glass, carbon, and Kevlar fiber reinforced rolymer composites. J. Mater. Res. Technol..

[70] Sabbatini B., Cambriani A., Cespi M., Palmieri G.F., Perinelli D.R., Bonacucina G. (2021). An Overview of Natural Polymers as Reinforcing Agents for 3D Printing. ChemEngineering.

[71] McIntyre J.E., Daniels P.N., Terms T.I.T., Committee D. (1995). Textile Terms and Definitions.

[72] Binczarski M.J., Malinowska J.Z., Berlowska J., Cieciura-Wloch W., Borowski S., Cieslak M., Puchowicz D., Witonska I.A. (2022). Concept for the Use of Cotton Waste Hydrolysates in Fermentation Media for Biofuel Production. Energies.

[73] TextileExchange. https://textileexchange.org/plant-fibers/.

[74] Nagdeve T., Dhara S., Tandulkar H., Jangde P., Ukani N., Chakole S. Design and Synthesis of Chassis of Automated Seed Sowing Robot for BT Cotton Seed. Proceedings of the 2020 IEEE International Students’ Conference on Electrical, Electronics and Computer Science (SCEECS).

[75] Frank E., Bauch V., Schultze-Gebhardt F., Herlinger K.-H. (2011). Fibers, 1. Survey. Ullmann’s Encyclopedia of Industrial Chemistry.

[76] Feughelman M. (2002). Natural protein fibers. J. Appl. Polym. Sci..

[77] Li Y., Hu Y.P., Hu C.J., Yu Y.H. (2008). Microstructures and Mechanical Properties of Natural Fibers. Adv. Mater. Res..

[78] Djafari Petroudy S.R., Fan M., Fu F. (2017). 3—Physical and mechanical properties of natural fibers. Advanced High Strength Natural Fibre Composites in Construction.

[79] Lee S.M. (1992). Handbook of Composite Reinforcements.

[80] Whitmer M. (2024). Types of Asbestos. www.asbestos.com.

[81] Lee T., Mischler S.E., Wolfe C. (2024). Classification of asbestos and their nonasbestiform analogues using FTIR and multivariate data analysis. J. Hazard. Mater..

[82] Gualtieri A.F., Tartaglia A. (2000). Thermal decomposition of asbestos and recycling in traditional ceramics. J. Eur. Ceram. Soc..

[83] Mustafayevich J.S., Xolmurodovich O.S. (2024). Inorganic Heat Insulation Materials. Gospodarka i Innowacje. https://gospodarkainnowacje-pl.openconference.us/index.php/issue_view_32/article/download/2259/2092.

[84] Algranti E., Ramos-Bonilla J.P., Terracini B., Santana V.S., Comba P., Pasetto R., Mazzeo A., Cavariani F., Trotta A., Marsili D. (2019). Prevention of Asbestos Exposure in Latin America within a Global Public Health Perspective. Ann. Glob. Health.

[85] Naz M., Rafiq A., Ikram M., Haider A., Ahmad S.O.A., Haider J., Naz S. (2021). Elimination of dyes by catalytic reduction in the absence of light: A review. J. Mater. Sci..

[86] Liu F., Pan L., Liu Y., Zhai G., Sha Z., Zhang X., Zhang Z., Liu Q., Yu S., Zhu L. (2024). Biobased fibers from natural to synthetic: Processing, manufacturing, and application. Matter.

[87] Gupta V.B., Gupta V.B., Kothari V.K. (1997). Solution-spinning processes. Manufactured Fibre Technology.

[88] Preston J. (2022). Man-Made Fibre. Encyclopedia Britannica. https://www.britannica.com/technology/man-made-fiber.

[89] Gupta V.B., Gupta V.B., Kothari V.K. (1997). Melt-spinning processes. Manufactured Fibre Technology.

[90] Hufenus R., Yan Y., Dauner M., Kikutani T. (2020). Melt-Spun Fibers for Textile Applications. Materials.

[91] Qin Y., Qin Y. (2016). 3—A brief description of textile fibers. Medical Textile Materials.

[92] Xia L., Xi P., Cheng B. (2015). A comparative study of UHMWPE fibers prepared by flash-spinning and gel-spinning. Mater. Lett..

[93] Hugill R., Ley K., Rademan K. (2020). Coming Full Circle: Innovating Towards Sustainable Man-Made Cellulosic Fibres.

[94] Gupta N., Kanth N. (2023). Heat Transfer Model for Silk Finishing Calender. Frontiers in Industrial and Applied Mathematics: Proceedings of the FIAM-2021, Punjab, India, 21–22 December 2021.

[95] Gobalakrishnan M., Saravanan D., Das S. (2020). Sustainable finishing process using natural ingredients. Sustainability in the Textile and Apparel Industries: Production Process Sustainability.

[96] Ramasamy R., Subramanian R.B. (2021). Synthetic textile and microfiber pollution: A review on mitigation strategies. Environ. Sci. Pollut. Res..

[97] Wang S., Lu A., Zhang L. (2016). Recent advances in regenerated cellulose materials. Prog. Polym. Sci..

[98] Shaikh T., Chaudhari S., Varma A. (2012). Viscose rayon: A legendary development in the manmade textile. Int. J. Eng. Res. Appl..

[99] Balkissoon S., Andrew J., Sithole B. (2022). Dissolving wood pulp production: A review. Biomass Convers. Biorefin..

[100] Karthik T., Gopalakrishnan D. (2014). Environmental analysis of textile value chain: An overview. Roadmap to Sustainable Textiles and Clothing: Environmental and Social Aspects of Textiles and Clothing Supply Chain.

[101] Mendes I.S., Prates A., Evtuguin D.V. (2021). Production of rayon fibres from cellulosic pulps: State of the art and current developments. Carbohydr. Polym..

[102] Kuchtová G., Herink P., Herink T., Chýlková J., Mikulášek P., Dušek L. (2023). From lab-scale to pilot-scale treatment of real wastewater from the production of rayon fiber. Process Saf. Environ. Prot..

[103] Zainul Armir N.A., Zulkifli A., Gunaseelan S., Palanivelu S.D., Salleh K.M., Che Othman M.H., Zakaria S. (2021). Regenerated cellulose products for agricultural and their potential: A review. Polymers.

[104] El Seoud O.A., Kostag M., Jedvert K., Malek N.I. (2020). Cellulose regeneration and chemical recycling: Closing the “cellulose gap” using environmentally benign solvents. Macromol. Mater. Eng..

[105] Sayyed A.J., Deshmukh N.A., Pinjari D.V. (2019). A critical review of manufacturing processes used in regenerated cellulosic fibres: Viscose, cellulose acetate, cuprammonium, LiCl/DMAc, ionic liquids, and NMMO based lyocell. Cellulose.

[106] Chawla S.P., Kanatt S.R., Sharma A.K., Ramawat K.G., Mérillon J.-M. (2015). Chitosan. Polysaccharides: Bioactivity and Biotechnology.

[107] Rinaudo M. (2006). Chitin and chitosan: Properties and applications. Prog. Polym. Sci..

[108] Kim C.-H., Park S.J., Yang D.H., Chun H.J., Chun H.J., Park K., Kim C.-H., Khang G. (2018). Chitosan for Tissue Engineering. Novel Biomaterials for Regenerative Medicine.

[109] Zhang S., Chen C., Duan C., Hu H., Li H., Li J., Liu Y., Ma X., Stavik J., Ni Y. (2018). Regenerated cellulose by the lyocell process, a brief review of the process and properties. BioResources.

[110] Parisi O.I., Curcio M., Puoci F., Puoci F. (2015). Polymer Chemistry and Synthetic Polymers. Advanced Polymers in Medicine.

[111] Su W.-F. (2013). Radical chain polymerization. Principles of Polymer Design and Synthesis.

[112] Braunecker W.A., Matyjaszewski K. (2007). Controlled/living radical polymerization: Features, developments, and perspectives. Prog. Polym. Sci..

[113] D’hooge D.R., Van Steenberge P.H.M., Reyniers M.-F., Marin G.B. (2016). The strength of multi-scale modeling to unveil the complexity of radical polymerization. Prog. Polym. Sci..

[114] Yilmaz G., Yagci Y. (2020). Light-induced step-growth polymerization. Prog. Polym. Sci..

[115] Van Steenberge P.H.M., Vandenbergh J., Reyniers M.-F., Junkers T., D’hooge D.R., Marin G.B. (2017). Kinetic Monte Carlo Generation of Complete Electron Spray Ionization Mass Spectra for Acrylate Macromonomer Synthesis. Macromolecules.

[116] Adegbola T., Agboola O., Fayomi O. (2020). Review of polyacrylonitrile blends and application in manufacturing technology: Recycling and environmental impact. Results Eng..

[117] Smirnova O., Kharitonov A., Belentsov Y. (2019). Influence of polyolefin fibers on the strength and deformability properties of road pavement concrete. J. Traffic Transp. Eng. Engl. Ed..

[118] Gurera D., Bhushan B. (2018). Fabrication of bioinspired superliquiphobic synthetic leather with self-cleaning and low adhesion. Colloids Surf. A Physicochem. Eng. Asp..

[119] Agrawal A., Kaur R., Walia R. (2017). PU foam derived from renewable sources: Perspective on properties enhancement: An overview. Eur. Polym. J..

[120] Novikov M.B., Borodulina T.A., Kotomin S.V., Kulichikhin V.G., Feldstein M.M. (2005). Relaxation properties of pressure-sensitive adhesives upon withdrawal of bonding pressure. J. Adhes..

[121] Gadhave R.V., Vineeth S. (2023). Synthesis of Microcrystalline Cellulose—Polyvinyl Alcohol Stabilized Polyvinyl Acetate Emulsion. Green Sustain. Chem..

[122] Aydemir D. (2014). The Lap Joint Shear Strength of Wood Materials Bonded by Cellulose Fiber-Reinforced Polyvinyl Acetate. Bioresources.

[123] Islam M.S. (2020). Polyvinyl Alcohol and Polyvinyl Acetate. Industrial Applications of Biopolymers and Their Environmental Impact.

[124] Bossion A., Heifferon K.V., Meabe L., Zivic N., Taton D., Hedrick J.L., Long T.E., Sardon H. (2019). Opportunities for organocatalysis in polymer synthesis via step-growth methods. Prog. Polym. Sci..

[125] Jaffe M., Easts A.J., Feng X. (2020). Polyester fibers. Thermal Analysis of Textiles and Fibers.

[126] Fiorillo C., Edeleva M., Trossaert L., Van Steenberge P., Cardon L., D’hooge D. Understanding the hydrolytic stability of the (co-)polyester polymer family. Proceedings of the Annual Meeting of the Belgian Polymer Group 2022 (BPG 2022).

[127] Trossaert L., De Vel M., Cardon L., Edeleva M. (2022). Lifting the Sustainability of Modified Pet-Based Multilayer Packaging Material with Enhanced Mechanical Recycling Potential and Processing. Polymers.

[128] Rabiei N., Kish M.H. (2022). Aminolysis of polyesters for cracking and structure clarifying: A review. Polym. Adv. Technol..

[129] Heidrich D., Gehde M. (2022). The 3-Phase Structure of Polyesters (PBT, PET) after Isothermal and Non-Isothermal Crystallization. Polymers.

[130] Vouyiouka S.N., Karakatsani E.K., Papaspyrides C.D. (2005). Solid state polymerization. Prog. Polym. Sci..

[131] Pang K., Kotek R., Tonelli A. (2006). Review of conventional and novel polymerization processes for polyesters. Prog. Polym. Sci..

[132] Thiele U.K. (2007). Polyester Bottle Resins Production, Processing, Properties and Recycling.

[133] Duh B. (2001). Reaction kinetics for solid-state polymerization of poly (ethylene terephthalate). J. Appl. Polym. Sci..

[134] Ketema A., Worku A. (2020). Review on intermolecular forces between dyes used for polyester dyeing and polyester fiber. J. Chem..

[135] Shogren R., Wood D., Orts W., Glenn G. (2019). Plant-based materials and transitioning to a circular economy. Sustain. Prod. Consum..

[136] Zhao S., Gao Z., Jiang G., Wang J., Miao X., Wan A. (2021). Effect of the dyeing process on thermal and dyeing properties of poly(butylene terephthalate) fibers. Text. Res. J..

[137] Sahoo S.K., Dash A.K. (2023). Sustainable polyester and caprolactam fibres. Sustainable Fibres for Fashion and Textile Manufacturing.

[138] Shukla D.K., Dey A., Singh A., Tripathi S.N., Bonda S., Saha S., Iyer P.K., Srivastava V.K., Jasra R.V. (2022). Disentangled ultrahigh molecular weight polyethylene thin film as a transparent substrate for flexible flat panel display. J. Appl. Polym. Sci..

[139] Ding Q., Soccio M., Lotti N., Cavallo D., Androsch R. (2019). Melt Crystallization of Poly(butylene 2,6-naphthalate). Chin. J. Polym. Sci..

[140] de Albuquerque T.L., Júnior J.E.M., de Queiroz L.P., Ricardo A.D.S., Rocha M.V.P. (2021). Polylactic acid production from biotechnological routes: A review. Int. J. Biol. Macromol..

[141] Soleyman E., Aberoumand M., Rahmatabadi D., Soltanmohammadi K., Ghasemi I., Baniassadi M., Abrinia K., Baghani M. (2022). Assessment of controllable shape transformation, potential applications, and tensile shape memory properties of 3D printed PETG. J. Mater. Res. Technol..

[142] Vasanthan N. (2009). Polyamide fiber formation: Structure, properties and characterization. Handbook of Textile Fibre Structure.

[143] Fan W., Wang Y., Liu R., Zou J., Yu X., Liu Y., Zhi C., Meng J. (2024). Textile production by additive manufacturing and textile waste recycling: A review. Environ. Chem. Lett..

[144] Vojdani M., Giti R. (2015). Polyamide as a denture base material: A literature review. J. Dent..

[145] Shioya M., Kikutani T., Sinclair R. (2015). Chapter 7—Synthetic Textile Fibres: Non-polymer Fibres. Textiles and Fashion.

[146] Polymer Science Learning Center Is Inorganic Glass an Inorganic Polymer?. https://pslc.ws/macrog/glass.htm.

[147] Chawla K.K., Buschow K.H.J., Cahn R.W., Flemings M.C., Ilschner B., Kramer E.J., Mahajan S., Veyssière P. (2001). Glass Fibers. Encyclopedia of Materials: Science and Technology.

[148] Chaudhary A., Gupta V., Teotia S., Nimanpure S., Rajak D.K., Brabazon D. (2021). Electromagnetic Shielding Capabilities of Metal Matrix Composites. Encyclopedia of Materials: Composites.

[149] Li A.-j., Xu J., Zhang F.-z., Song Y.-h., Wang J.-h., Ye C., Zhu S.-p. (2024). A microstructure-based model for the thermal conductivity of carbon fibers. Mater. Sci. Eng. B.

[150] Chennam P.K., Kachlík M., Říhová M., Čičmancová V., Maca K., Macak J.M. (2024). Synthesis of centrifugally spun polyacrylonitrile-carbon fibers. J. Mater. Res. Technol..

[151] Fazeli M., Islam S., Baniasadi H., Abidnejad R., Schlapp-Hackl I., Hummel M., Lipponen J. (2024). Exploring the potential of regenerated Ioncell fiber composites: A sustainable alternative for high-strength applications. Green Chem..

[152] Krithikaa D., Chandramohan P., Suresh G., Rathinasabapathi G., Madheswaran D.K., Faisal A.M. (2024). A study on: Design and fabrication of E-glass fiber reinforced IPN composite chain plates for low duty chain drives. Mater. Today Proc..

[153] Ilyas R., Zuhri M., Norrrahim M.N.F., Misenan M.S.M., Jenol M.A., Samsudin S.A., Nurazzi N., Asyraf M., Supian A., Bangar S.P. (2022). Natural fiber-reinforced polycaprolactone green and hybrid biocomposites for various advanced applications. Polymers.

[154] Muthukumar C., Krishnasamy S., Thiagamani S.M.K., Nagarajan R., Siengchin S. (2022). Thermal characterization of the natural fiber-based hybrid composites: An overview. Natural Fiber-Reinforced Composites: Thermal Properties and Applications.

[155] Kakati A., Banerjee A., Das P., Saha B., Goyary D., Karmakar S., Kishor S., Bhutia Y.D., Chattopadhyay P. (2023). Development of insecticide-impregnated polyester/cotton blend fabric and assessment of their repellent characteristics against Cimex lectularius and dengue vectors Aedes albopictus and Aedes aegypti. Parasites Vectors.

[156] Li Y., Sun L., Wang H., Wang S., Jin X., Lu Z., Dong C. (2023). A novel composite coating containing P/N/B and bio-based compounds for flame retardant modification of polyester/cotton blend fabrics. Colloids Surf. A Physicochem. Eng. Asp..

[157] Nesa S.H.S., Tarangini K. (2023). A review on augmentation of natural fabric materials with novel bio/nanomaterials and their multifunctional perspectives. Hybrid Adv..

[158] Birkocak D.T. (2022). Effects of Needle Size and Sewing Thread on Seam Quality of Traditional Fabrics. Text. Appar..

[159] Wakida T., Tokuyama T., Doi C., Lee M., Jeong D.S., Ishida S. (2004). Mechanical properties of polyester/cotton and polyester/rayon fabrics treated with ammonia-gas. Sen’i Gakkaishi.

[160] İlhan Ö. (2013). Effects of pre-and intermediate causticisation on pattern formation and fastness properties of three-and two-bath dyeings of woven polyester/cationic dyeable polyester/rayon fabrics. Text. Appar..

[161] Najar S.S., Amani M., Hasani H. (2003). Analysis of blend irregularities and fiber migration index of wool/acrylic blended worsted yarns by using an image-analysis technique. J. Text. Inst..

[162] Matayeva A., Madsen A.S., Biller P. (2023). Evaluation of different fiber impurities on hydrothermal liquefaction of mixed textile waste. Resour. Conserv. Recycl..

[163] Koch H.C., Schmelzeisen D., Gries T. (2021). 4D textiles made by additive manufacturing on pre-stressed textiles—An overview. Actuators.

[164] Panda H. (2016). Modern Technology of Textile Dyes & Pigments.

[165] Özer M.S., Gaan S. (2022). Recent developments in phosphorus based flame retardant coatings for textiles: Synthesis, applications and performance. Prog. Org. Coat..

[166] Gulati R., Sharma S., Sharma R.K. (2022). Antimicrobial textile: Recent developments and functional perspective. Polym. Bull..

[167] Sankaran A., Kamboj A., Samant L., Jose S. (2021). Synthetic and natural UV protective agents for textile finishing. Innovative and Emerging Technologies for Textile Dyeing and Finishing.

[168] Mahapatra A., Patil S., Arputharaj A., Gotmare V., Patil P. (2020). Effect of textile softeners on BTCA treated cotton fabric. Indian J. Fibre Text. Res..

[169] Haule L.V., Nambela L. (2023). Advances in waterproof technologies in textiles. Functional and Technical Textiles.

[170] Almasry S., Rabea H., Hany H., Gerges M., Rafaat Y., Maamoun D., Mohamed H., Khattab T.A. (2023). Eco-Friendly Multi-Finishing Properties of Polyester Fabrics. J. Text. Color. Polym. Sci..

[171] Kahoush M., Kadi N. (2022). Towards sustainable textile sector: Fractionation and separation of cotton/polyester fibers from blended textile waste. Sustain. Mater. Technol..

[172] Chen Z., Sun H., Kong W., Chen L., Zuo W. (2023). Closed-loop utilization of polyester in the textile industry. Green Chem..

[173] Stefan D.S., Bosomoiu M., Stefan M. (2022). Methods for Natural and Synthetic Polymers Recovery from Textile Waste. Polymers.

[174] Ruschel-Soares R., Contin B., Siqueira M.U., Fernandes P.R.B., Soares N.R., Baruque-Ramos J. (2022). Environmental Impacts of Polyester-Cotton Blend Compared to Cotton Fiber in Brazil. Mater. Circ. Econ..

[175] El Darai T., Ter-Halle A., Blanzat M., Despras G., Sartor V., Bordeau G., Lattes A., Franceschi S., Cassel S., Chouini-Lalanne N. (2024). Chemical recycling of polyester textile wastes: Shifting towards sustainability. Green Chem..

[176] Allen E., Henninger C.E., Garforth A., Asuquo E. (2024). Microfiber Pollution: A Systematic Literature Review to Overcome the Complexities in Knit Design to Create Solutions for Knit Fabrics. Environ. Sci. Technol..

[177] Franco Urquiza E.A. (2024). Advances in Additive Manufacturing of Polymer-Fused Deposition Modeling on Textiles: From 3D Printing to Innovative 4D Printing—A Review. Polymers.

[178] Sawant Y., Admuthe L. (2024). Characterization of needle-punched nonwoven fabric air filter using computer vision—A review. J. Text. Inst..

[179] Barman N.K., Bhattacharya S.S., Alagirusamy R. (2024). Textile structures in concrete reinforcement. Text. Prog..

[180] Šajn Gorjanc D., Kostajnšek K. (2024). Permeable Properties of Hygienic Nonwovens Bonded Using Mechanical, Chemical, and Thermal Techniques. Polymers.

[181] Klemm C., Kaufman S. (2024). The importance of circular attributes for consumer choice of fashion and textile products in Australia. Sustain. Prod. Consum..

[182] Kilinc M., Korkmaz G., Kilinc N., Kut D., Jose S., Thomas S., Basu G. (2024). Chapter 19—The use of wool fiber in technical textiles and recent developments. The Wool Handbook.

[183] Zhang Q., Cheng H., Zhang S., Li Y., Li Z., Ma J., Liu X. (2024). Advancements and challenges in thermoregulating textiles: Smart clothing for enhanced personal thermal management. Chem. Eng. J..

[184] Maity S., Singha K., Pandit P., Maity S., Singha K., Pandit P. (2023). 1—Introduction to functional and technical textiles. Functional and Technical Textiles.

[185] Panneerselvam D., Murugesan P., Moses J.A. (2024). Silk fibroin and prospective applications in the food sector. Eur. Polym. J..

[186] Deutsches Institut für Normung (2023). DIN EN ISO 5157, Textilien—Umweltaspekte—Begriffe (ISO 5157:2023): Textiles—Environmental Aspects—Vocabulary (ISO 5157:2023).

[187] Dodampegama S., Hou L., Asadi E., Zhang G., Setunge S. (2024). Revolutionizing construction and demolition waste sorting: Insights from artificial intelligence and robotic applications. Resour. Conserv. Recycl..

[188] (2013). Plastics—Vocabulary. Kunststoffe–Fachwörterverzeichnis.

[189] Sandin G., Peters G.M. (2018). Environmental impact of textile reuse and recycling—A review. J. Clean. Prod..

[190] Azad A.K., Haq U.N., Khairul Akter M.M., Uddin M.A., Muthu S.S. (2024). Recycling Practices of Pre-Consumer Waste Generated from Textile Industry. Sustainable Manufacturing Practices in the Textiles and Fashion Sector.

[191] (2007). Standard Guide for Development of ASTM Standards Relating to Recycling and Use of Recycled Plastics.

[192] Muthu S.S., Li Y., Hu J.Y., Ze L. (2012). Carbon footprint reduction in the textile process chain: Recycling of textile materials. Fibers Polym..

[193] (2020). Directive 2008/122/EC of the European parliament and of the council. Fundamental Texts On European Private Law.

[194] Stubbe B., Van Vrekhem S., Huysman S., Tilkin R.G., De Schrijver I., Vanneste M. (2024). White Paper on Textile Fibre Recycling Technologies. Sustainability.

[195] Zamani B., Sandin G., Peters G.M. (2017). Life cycle assessment of clothing libraries: Can collaborative consumption reduce the environmental impact of fast fashion?. J. Clean. Prod..

[196] Sood K., Gosselin S., Seifali Abbas-Abadi M., De Coensel N., Lizardo-Huerta J.-C., El Bakali A., Van Geem K.M., Gasnot L., Tran L.-S. (2024). Experimental Detection of Oxygenated Aromatics in an Anisole-Blended Flame. Energy Fuels.

[197] United States Environmental Protection Agency Textiles: Material-Specific Data. https://www.epa.gov/facts-and-figures-about-materials-waste-and-recycling/textiles-material-specific-data.

[198] Baskan-Bayrak H., Karakas H., Jose S., Thomas S., Basu G. (2024). Chapter 8—Morphology and chemical structure of a wool fiber. The Wool Handbook.

[199] Fazalur R., Fatima A., Adeel S., Qayyum M.A., Tanveer H.A., Ahmed S., Shabbir M. (2024). Biosynthesis Application and Modification of Protein Fiber. Biopolymers in the Textile Industry: Opportunities and Limitations.

[200] Kadam V., Saini H., Verma K., Dubey I., Verma P., Jose S., Thomas S., Basu G. (2024). Chapter 26—Prospects of wool and woolen products. The Wool Handbook.

[201] Hole G., Hole A.S. (2020). Improving recycling of textiles based on lessons from policies for other recyclable materials: A minireview. Sustain. Prod. Consum..

[202] Li X., Wang L., Ding X. (2021). Textile supply chain waste management in China. J. Clean. Prod..

[203] Cura K., Rintala N., Kamppuri T., Saarimäki E., Heikkilä P. (2021). Textile Recognition and Sorting for Recycling at an Automated Line Using Near Infrared Spectroscopy. Recycling.

[204] Karell E., Niinimäki K. (2019). Addressing the dialogue between design, sorting and recycling in a circular economy. Des. J..

[205] Jordeva S., Tomovska E., Zhezhova S., Golomeova S., Dimitrijeva V. (2020). Textile waste management practices. Contemporary trends and innovations in the textile industry CT&ITI 2020. Mech. Eng..

[206] Tang K.H.D. (2023). State of the Art in Textile Waste Management: A Review. Textiles.

[207] Chavan R. (2014). Environmental sustainability through textile recycling. J. Text. Sci. Eng..

[208] Yalcin-Enis I., Kucukali-Ozturk M., Sezgin H., Gothandam K.M., Ranjan S., Dasgupta N., Lichtfouse E. (2019). Risks and Management of Textile Waste. Nanoscience and Biotechnology for Environmental Applications.

[209] Duhoux T., Lingås D. (2024). Volumes and destruction of returned and unsold textiles in Europe’s circular economy. ETC CE Rep..

[210] Puglia M., Parker L., Clube R.K.M., Demirel P., Aurisicchio M. (2024). The circular policy canvas: Mapping the European Union’s policies for a sustainable fashion textiles industry. Resour. Conserv. Recycl..

[211] Charnley F., Cherrington R., Mueller F., Jain A., Nelson C., Wendland S., Ventosa S. (2024). Retaining product value in post-consumer textiles: How to scale a closed-loop system. Resour. Conserv. Recycl..

[212] Büyükaslan E., Jevšnık S., Kalaoglu F. (2015). A Sustainable Approach to Collect Post-Consumer Textile Waste in Developing Countries. Marmara Fen Bilim. Derg..

[213] Palm D., Elander M., Watson D., Kiørboe N., Salmenperä H., Dahlbo H., Moliis K., Lyng K.A., Valente C., Gíslason S. (2014). Towards a Nordic Textile Strategy: Collection, Sorting, Reuse and Recycling of Textiles.

[214] Wang Y. (2010). Fiber and Textile Waste Utilization. Waste Biomass Valorization.

[215] Manglani H., Hodge G.L., Oxenham W. (2019). Application of the Internet of Things in the textile industry. Text. Prog..

[216] Hack-Polay D., Rahman M., Billah M.M., Al-Sabbahy H.Z. (2020). Big data analytics and sustainable textile manufacturing. Manag. Decis..

[217] Wojnowska-Baryła I., Bernat K., Zaborowska M., Kulikowska D. (2024). The Growing Problem of Textile Waste Generation—The Current State of Textile Waste Management. Energies.

[218] Dursun E., Ulker Y., Gunalay Y. (2023). Blockchain’s potential for waste management in textile industry. Manag. Environ. Qual. Int. J..

[219] Martikkala A., Mayanti B., Helo P., Lobov A., Ituarte I.F. (2023). Smart textile waste collection system—Dynamic route optimization with IoT. J. Environ. Manag..

[220] Lingås D., Manshoven S., Mortensen L. (2023). EU exports of used textiles in Europe’s circular economy. ETC CE Rep..

[221] Hicks C., Dietmar R., Eugster M. (2005). The recycling and disposal of electrical and electronic waste in China—Legislative and market responses. Environ. Impact Assess. Rev..

[222] Abagnato S., Rigamonti L., Grosso M. (2024). Life cycle assessment applications to reuse, recycling and circular practices for textiles: A review. Waste Manag..

[223] Feng Y., Hao H., Lu H., Chow C.L., Lau D. (2024). Exploring the development and applications of sustainable natural fiber composites: A review from a nanoscale perspective. Compos. Part B Eng..

[224] Shu D., Li W., Han B., An F., Zhang Y., Cao S., Liu R. (2024). Cleaner reactive dyeing with the recycled dyeing wastewater. J. Environ. Chem. Eng..

[225] Akter M., Anik H.R., Mahmud S., Arya R.K., Verros G.D., Verma O.P., Hussain C.M. (2024). Conversion of Textile Waste to Wealth and Their Industrial Utilization. From Waste to Wealth.

[226] Zhou C., Han G., Via B.K., Song Y., Gao S., Jiang W. (2019). Rapid identification of fibers from different waste fabrics using the near-infrared spectroscopy technique. Text. Res. J..

[227] Pettersson A. (2015). Towards Recycling of Textile Fibers: Separation and Characterization of Textile Fibers and Blends.

[228] Bianchi S., Bartoli F., Bruni C., Fernandez-Avila C., Rodriguez-Turienzo L., Mellado-Carretero J., Spinelli D., Coltelli M.-B. (2023). Opportunities and Limitations in Recycling Fossil Polymers from Textiles. Macromol.

[229] Iezzi B., Coon A., Cantley L., Perkins B., Doran E., Wang T., Rothschild M., Shtein M. (2023). Polymeric Photonic Crystal Fibers for Textile Tracing and Sorting. Adv. Mater. Technol..

[230] Zhou J., Yu L., Ding Q., Wang R. (2019). Textile Fiber Identification Using Near-Infrared Spectroscopy and Pattern Recognition. Autex Res. J..

[231] Suciyati S.W., Manurung P., Sembiring S., Situmeang R. (2021). Comparative study of *Cladophora* sp. cellulose by using FTIR and XRD. J. Phys. Conf. Ser..

[232] Singh R.K., Ruj B., Sadhukhan A.K., Gupta P. (2020). A TG-FTIR investigation on the co-pyrolysis of the waste HDPE, PP, PS and PET under high heating conditions. J. Energy Inst..

[233] Zhang L., Li X., Zhang S., Gao Q., Lu Q., Peng R., Xu P., Shang H., Yuan Y., Zou H. (2021). Micro-FTIR combined with curve fitting method to study cellulose crystallinity of developing cotton fibers. Anal. Bioanal. Chem..

[234] Peets P., Kaupmees K., Vahur S., Leito I. (2019). Reflectance FT-IR spectroscopy as a viable option for textile fiber identification. Herit. Sci..

[235] Peets P., Leito I., Pelt J., Vahur S. (2017). Identification and classification of textile fibres using ATR-FT-IR spectroscopy with chemometric methods. Spectrochim. Acta Part A Mol. Biomol. Spectrosc..

[236] Blanch-Perez-del-Notario C., Saeys W., Lambrechts A. (2019). Hyperspectral imaging for textile sorting in the visible–near infrared range. J. Spectr. Imaging.

[237] Alpert C., Turkowski M., Tasneem T. (2021). Scalability Solutions for Automated Textile Sorting: A Case Study on How Dynamic Capabilities can Overcome Scalability Challenges.

[238] Chen H., Tan C., Lin Z. (2020). Quantitative determination of the fiber components in textiles by near-infrared spectroscopy and extreme learning machine. Anal. Lett..

[239] Liu Z., Li W., Wei Z. (2019). Qualitative classification of waste textiles based on near infrared spectroscopy and the convolutional network. Text. Res. J..

[240] Mäkelä M., Geladi P. (2018). Hyperspectral near infrared imaging quantifies the heterogeneity of carbon materials. Sci. Rep..

[241] Jiang Y., Li C. (2015). mRMR-based feature selection for classification of cotton foreign matter using hyperspectral imaging. Comput. Electron. Agric..

[242] Du W., Zheng J., Li W., Liu Z., Wang H., Han X. (2022). Efficient Recognition and Automatic Sorting Technology of Waste Textiles Based on Online Near infrared Spectroscopy and Convolutional Neural Network. Resour. Conserv. Recycl..

[243] Li X., Xu X., Liu Z. (2020). Cryogenic grinding performance of scrap tire rubber by devulcanization treatment with ScCO_2_. Powder Technol..

[244] Wang Z., Jiang Y., Pan C. (2021). Mechanochemical devulcanization of waste tire rubber in high pressure water jet pulverization. Prog. Rubber Plast. Recycl. Technol..

[245] Wang Z., Zeng D. (2021). Preparation of devulcanized ground tire rubber with supercritical carbon dioxide jet pulverization. Mater. Lett..

[246] Abbas-Abadi M.S., Kusenberg M., Shirazi H.M., Goshayeshi B., Van Geem K.M. (2022). Towards full recyclability of end-of-life tires: Challenges and opportunities. J. Clean. Prod..

[247] Grammelis P., Margaritis N., Dallas P., Rakopoulos D., Mavrias G. (2021). A Review on Management of End of Life Tires (ELTs) and Alternative Uses of Textile Fibers. Energies.

[248] Aljannahi A., Alblooshi R.A., Alremeithi R.H., Karamitsos I., Ahli N.A., Askar A.M., Albastaki I.M., Ahli M.M., Modak S. (2022). Forensic Analysis of Textile Synthetic Fibers Using a FT-IR Spectroscopy Approach. Molecules.

[249] Rasheed A., Ahmad S., Rasheed A., Nawab Y. (2020). Classification of Technical Textiles. Fibers for Technical Textiles.

[250] Bartl A., Letcher T.M., Vallero D.A. (2019). Chapter 16—End-of-Life Textiles. Waste.

[251] Rao N., Salvidge C., Doriza A., Downing P. (2022). Citizen Insights: Estimating the Longevity of Home Textiles in the UK.

[252] Bukhari M.A., Carrasco-Gallego R., Ponce-Cueto E. (2018). Developing a national programme for textiles and clothing recovery. Waste Manag. Res..

[253] Paras M.K., Ekwall D., Pal R., Curteza A., Chen Y., Wang L. (2018). An Exploratory Study of Swedish Charities to Develop a Model for the Reuse-Based Clothing Value Chain. Sustainability.

[254] Palm D., Elander M., Watson D., Kiørboe N., Salmenperä H., Dahlbo H., Rubach S., Hanssen O.J., Gíslason S., Ingulfsvann A.S. (2015). A Nordic Textile Strategy: Part II: A Proposal for Increased Collection, Sorting, Reuse and Recycling of Textiles.

[255] Ivana K. (2023). Waste Framework Directive: A More Sustainable Use of Natural Resources. EPRS: European Parliamentary Research Service. https://policycommons.net/artifacts/10880460/waste-framework-directive/11758402/.

[256] Hardy D., Wickenden R., McLaren A. (2020). Electronic textile reparability. J. Clean. Prod..

[257] All-Party Parliamentary Sustainable Resource Group (2014). Remanufacturing: Towards a Resource Efficient Economy. https://www.policyconnect.org.uk/research/report-remanufacturing-towards-resource-efficient-economy.

[258] Lund R.T. (1984). Remanufacturing: The Experience of the United States and Implications for Developing Countries.

[259] Sinha P., Muthu S.S., Dissanayake G., Sinha P., Muthu S.S., Dissanayake G. (2016). The Remanufacturing Industry and Fashion. Remanufactured Fashion.

[260] Stanescu M.D. (2021). State of the art of post-consumer textile waste upcycling to reach the zero waste milestone. Environ. Sci. Pollut. Res..

[261] Ghosh B. (2024). Climate Change and the Female Sex: An Intangible Connection. Gender, Environment and Sustainable Development.

[262] Shirvanimoghaddam K., Motamed B., Ramakrishna S., Naebe M. (2020). Death by waste: Fashion and textile circular economy case. Sci. Total Environ..

[263] Biermaier C., Petz P., Bechtold T., Pham T. (2023). Investigation of the Functional Ageing of Conductive Coated Fabrics under Simulated Washing Conditions. Materials.

[264] Bresee R.R. (1986). General effects of ageing on textiles. J. Am. Inst. Conserv..

[265] Hawkins W.L. (1984). Polymer Degradation and Stabilization.

[266] Castellani V., Sala S., Mirabella N. (2015). Beyond the throwaway society: A life cycle-based assessment of the environmental benefit of reuse. Integr. Environ. Assess. Manag..

[267] Commission E., Duhoux T., Maes E., Hirschnitz-Garbers M., Peeters K., Asscherickx L., Christis M., Stubbe B., Directorate-General for Internal Market, Industry, Entrepreneurship and SMEs (2021). Study on the Technical, Regulatory, Economic and Environmental Effectiveness of Textile Fibres Recycling—Final Report.

[268] (2023). Terra Technical Monitoring of Optical Sorting, Recognition and Disassembly Technologies for Textiles at European Scale. Re-fashion. https://refashion.fr/pro/sites/default/files/rapport-etude/240428_Synth%C3%A8se_Veille-technos-tri-d%C3%A9lissage_VF-EN.pdf.

[269] Record A., Harscoet E., Chouvenc S. (2022). Chemical and physico-chemical recycling of plastic waste. Tech. L’ingenieur.

[270] Egan J., Wang S., Shen J., Baars O., Moxley G., Salmon S. (2023). Enzymatic textile fiber separation for sustainable waste processing. Resour. Environ. Sustain..

[271] Haule L.V. (2016). Textile Recycling: A Review. https://api.semanticscholar.org/CorpusID:113949423.

[272] Malik R.K., Goswami K.K., Goswami K.K. (2018). 14—Processing and finishing in carpet. Advances in Carpet Manufacture.

[273] Maione D. (2023). Recrafting Futures: Post-Material Transformations Toward Clothing Longevity.

[274] Ecole Nationale Supérieure des Arts et Industries Textiles (2014). Étude des Perturbateurs et Facilitateurs au Recyclage des Textiles et Linges de Maison. Refashion. https://refashion.fr/eco-design/sites/default/files/fichiers/%C3%89tude%20des%20perturbateurs%20et%20facilitateurs%20au%20recyclage%20des%20textiles%20et%20linges%20de%20maison.pdf.

[275] Valvan Textile Sorting Solutions for the Recycling Industry. https://www.valvan.com/en/solutions/textile-sorting-recycling.

[276] CETIA Innovation Platform Dedicated to the Recyclability of Textile and Leather Articles. https://cetia.tech/home-en/.

[277] Schuch A. (2016). The chemical recycle of cotton. Rev. Produção E Desenvolv..

[278] Sharma S., Kaur A. (2018). Various methods for removal of dyes from industrial effluents—A review. Indian J. Sci. Technol..

[279] Verma A.K., Dash R.R., Bhunia P. (2012). A review on chemical coagulation/flocculation technologies for removal of colour from textile wastewaters. J. Environ. Manag..

[280] Hossain M.S., Shenashen M.A., Awual M.E., Rehan A.I., Rasee A.I., Waliullah R.M., Kubra K.T., Salman M.S., Sheikh M.C., Hasan M.N. (2024). Benign separation, adsorption, and recovery of rare-earth Yb(III) ions with specific ligand-based composite adsorbent. Process Saf. Environ. Prot..

[281] Awual M.E., Salman M.S., Hasan M.M., Hasan M.N., Kubra K.T., Sheikh M.C., Rasee A.I., Rehan A.I., Waliullah R.M., Hossain M.S. (2024). Ligand imprinted composite adsorbent for effective Ni(II) ion monitoring and removal from contaminated water. J. Ind. Eng. Chem..

[282] Awual M.R. (2019). Innovative composite material for efficient and highly selective Pb(II) ion capturing from wastewater. J. Mol. Liq..

[283] Rasee A.I., Awual E., Rehan A.I., Hossain M.S., Waliullah R.M., Kubra K.T., Sheikh M.C., Salman M.S., Hasan M.N., Hasan M.M. (2023). Efficient separation, adsorption, and recovery of Samarium(III) ions using novel ligand-based composite adsorbent. Surf. Interfaces.

[284] Yousef S., Tatariants M., Tichonovas M., Kliucininkas L., Lukošiūtė S.-I., Yan L. (2020). Sustainable green technology for recovery of cotton fibers and polyester from textile waste. J. Clean. Prod..

[285] Slama N.E.H., Masmoudi G., Fizer M., Mariychuk R., Dhaouadi H. (2024). Comprehensive study of Fenton reaction efficiency on textile wastewater treatment from dye solution to real effluent with emphasis on Fukui function analysis. J. Mol. Liq..

[286] Liu X., Wang J. (2024). Decolorization and degradation of crystal violet dye by electron beam radiation: Performance, degradation pathways, and synergetic effect with peroxymonosulfate. Environ. Pollut..

[287] Powar A. (2021). LCA and Eco-Design in the Field of Chemicals Removal from Textile Waste for Textile Recycling.

[288] Määttänen M., Gunnarsson M., Wedin H., Stibing S., Olsson C., Köhnke T., Asikainen S., Vehviläinen M., Harlin A. (2021). Pre-treatments of pre-consumer cotton-based textile waste for production of textile fibres in the cold NaOH(aq) and cellulose carbamate processes. Cellulose.

[289] Baloyi R.B., Gbadeyan O.J., Sithole B., Chunilall V. (2024). Recent advances in recycling technologies for waste textile fabrics: A review. Text. Res. J..

[290] Le K. (2018). Textile Recycling Technologies, Colouring and Finishing Methods. UBC Sustainability Scholars Report. https://sustain.ubc.ca/about/resources/textile-recycling-technologies-colouring-and-finishing-methods.

[291] Schmidt C., Berghahn E., Ilha V., Granada C.E. (2019). Biodegradation potential of Citrobacter cultures for the removal of amaranth and congo red azo dyes. Int. J. Environ. Sci. Technol..

[292] Haslinger S., Wang Y., Rissanen M., Lossa M.B., Tanttu M., Ilen E., Määttänen M., Harlin A., Hummel M., Sixta H. (2019). Recycling of vat and reactive dyed textile waste to new colored man-made cellulose fibers. Green Chem..

[293] Björquist S. (2017). Separation for Regeneration-Chemical Recycling of Cotton and Polyester Textiles.

[294] Gholamzad E., Karimi K., Masoomi M. (2014). Effective conversion of waste polyester–cotton textile to ethanol and recovery of polyester by alkaline pretreatment. Chem. Eng. J..

[295] Haule L.V., Carr C.M., Rigout M. (2014). Investigation into the removal of an easy-care crosslinking agent from cotton and the subsequent regeneration of lyocell-type fibres. Cellulose.

[296] Rescoll INDAR Debonding Process: Structural Debondable Adhesive Used for Ground Testing of GAIA Segments. https://rescoll.fr/indar-debonding-process-structural-debondable-adhesive-used-for-ground-testing-of-gaia-segments__trashed/.

[297] Olive M., Bergara T., Di-Tomaso J., Plouraboue T. Latest Achievements in the Field of Assembling Metals and Composites. https://rescoll.fr/wp-content/uploads/2015/12/MMP2015-28-paper.x66374.pdf.

[298] Collins D.M. Separating Polymer from Composite Structures. https://patents.google.com/patent/WO2018035565A1/en.

[299] Ball D.L., Hance M.H. Process for Recycling Denim Waste. https://patents.google.com/patent/US5369861A/en.

[300] Sheikh M.C., Hasan M.M., Hasan M.N., Salman M.S., Kubra K.T., Awual M.E., Waliullah R.M., Rasee A.I., Rehan A.I., Hossain M.S. (2023). Toxic cadmium(II) monitoring and removal from aqueous solution using ligand-based facial composite adsorbent. J. Mol. Liq..

[301] Yurtaslan Ö., Altun Kurtoğlu Ş., Yılmaz D. (2022). Closed-loop Mechanical Recycling Opportunities in Industrial Cotton Wastes. J. Nat. Fibers.

[302] Panda S.K.B.C., Sen K., Mukhopadhyay S. (2021). Sustainable pretreatments in textile wet processing. J. Clean. Prod..

[303] Piribauer B., Bartl A. (2019). Textile recycling processes, state of the art and current developments: A mini review. Waste Manag. Res..

[304] Saxena S., Raja A., Arputharaj A. (2017). Challenges in sustainable wet processing of textiles. Textiles and Clothing Sustainability: Sustainable Textile Chemical Processes.

[305] Allen T.W. Garnett Machine. https://patents.google.com/patent/US1630158A/en.

[306] Lambert M. (2004). ’Cast-off Wearing Apparell’: The consumption and distribution of second-hand clothing in northern England during the long eighteenth century. Text. Hist..

[307] Pensupa N., Leu S.-Y., Hu Y., Du C., Liu H., Jing H., Wang H., Lin C.S.K. (2017). Recent Trends in Sustainable Textile Waste Recycling Methods: Current Situation and Future Prospects. Top. Curr. Chem..

[308] Wang Y. (2006). Recycling in Textiles.

[309] Gulich B. (2006). 9—Development of products made of reclaimed fibres. Recycling in Textiles.

[310] Mowafi S., Mashaly H., El-Sayed H. (2020). Towards water-saving textile wet processing. Part 1: Scouring and dyeing. Egypt. J. Chem..

[311] Lindström K., Sjöblom T., Persson A., Kadi N. (2020). Improving Mechanical Textile Recycling by Lubricant Pre-Treatment to Mitigate Length Loss of Fibers. Sustainability.

[312] Payne A., Muthu S.S. (2015). 6—Open- and closed-loop recycling of textile and apparel products. Handbook of Life Cycle Assessment (LCA) of Textiles and Clothing.

[313] Madhav S., Ahamad A., Singh P., Mishra P.K. (2018). A review of textile industry: Wet processing, environmental impacts, and effluent treatment methods. Environ. Qual. Manag..

[314] Khan W.S., Asmatulu E., Uddin M.N., Asmatulu R. (2022). Recycling and Reusing of Engineering Materials: Recycling for Sustainable Developments.

[315] Langley K.D., Kim Y.K. (2006). Manufacturing nonwovens and other products using recycled fibers containing spandex. Recycling in Textiles.

[316] Textile Exchange (2021). Textile Exchange Guide to Recycled Inputs. https://textileexchange.org/app/uploads/2021/09/GRS-202-V1.0-Textile-Exchange-Guide-to-Recycled-Inputs.pdf.

[317] Dissanayake D.G.K., Weerasinghe D.U. (2021). Fabric Waste Recycling: A Systematic Review of Methods, Applications, and Challenges. Mater. Circ. Econ..

[318] Tshifularo C.A., Patnaik A., Nayak R. (2020). 13—Recycling of plastics into textile raw materials and products. Sustainable Technologies for Fashion and Textiles.

[319] Cao H., Cobb K., Yatvitskiy M., Wolfe M., Shen H. (2022). Textile and Product Development from End-of-Use Cotton Apparel: A Study to Reclaim Value from Waste. Sustainability.

[320] Esteve-Turrillas F.A., de la Guardia M. (2017). Environmental impact of Recover cotton in textile industry. Resour. Conserv. Recycl..

[321] Aronsson J., Persson A. (2020). Tearing of post-consumer cotton T-shirts and jeans of varying degree of wear. J. Eng. Fibers Fabr..

[322] De Smit K., Wieme T., Marien Y.W., Van Steenberge P.H.M., D’Hooge D.R., Edeleva M. (2022). Multi-scale reactive extrusion modelling approaches to design polymer synthesis, modification and mechanical recycling. React. Chem. Eng..

[323] Ragaert K., Delva L., Van Geem K. (2017). Mechanical and chemical recycling of solid plastic waste. Waste Manag..

[324] Ogutgen M.K., Vatansevdi M.K., Ozturk B., Caliskan Akduman M. Plastic Recycling in a R&d Office: Recycling of Plastic Waste as Granules and Fibre. Proceedings of the 11th Global Conference on Global Warming (GCGW 2023).

[325] Kunchi Mon S.Z.B. (2019). Polyamide 6 Fibre Recycling by Twin-Screw Melt Extrusion of Mixed Thermoplastic Polymers.

[326] Ozmen S.C., Ozkoc G., Serhatli I.E. (2021). Effect of reactive extrusion process parameters on thermal, mechanical, and physical properties of recycled polyamide-6: Comparison of two novel chain extenders. J. Macromol. Sci. Part B.

[327] Luiken A., Brinks G., BMA-Techne, Almelo Reflow Amsterdam Circular Textiles Pilot-A Primer on Textile Recycling. https://reflowproject.eu/wp-content/uploads/2021/05/REFLOW_BOOKLET_TEXTILE_WHEEL-compressed.pdf.

[328] Altun S., Ulcay Y. (2004). Improvement of Waste Recycling in PET Fiber Production. J. Polym. Environ..

[329] Qin Y., Qu M., Kaschta J., Allen V., Schubert D.W. (2020). Studies on recycled polyester. Recycled Polyester: Manufacturing, Properties, Test Methods, and Identification.

[330] Ceretti D.V., Edeleva M., Cardon L., D’hooge D.R. (2023). Molecular pathways for polymer degradation during conventional processing, additive manufacturing, and mechanical recycling. Molecules.

[331] Jang J.Y., Sadeghi K., Seo J. (2022). Chain-extending modification for value-added recycled PET: A review. Polym. Rev..

[332] Guillén-Mallette J., Ríos-Soberanis C.R., Enríquez-Reyes J. (2021). Discoloration of green PET bottles recycled with chemical agents by reactive extrusion. J. Elastomers Plast..

[333] Wang Y., Chen S., Guang S., Wang Y., Zhang X., Chen W. (2019). Continuous post-polycondensation of high-viscosity poly (ethylene terephthalate) in the molten state. J. Appl. Polym. Sci..

[334] Benvenuta-Tapia J.J., Vivaldo-Lima E., Guerrero-Santos R. (2019). Effect of copolymers synthesized by nitroxide-mediated polymerization as chain extenders of postconsumer poly(ethylene terephthalate) waste. Polym. Eng. Sci..

[335] De Smit K., Marien Y.W., Van Steenberge P.H.M., D’Hooge D.R., Edeleva M. (2023). Playing with process conditions to increase the industrial sustainability of poly(lactic acid)-based materials. React. Chem. Eng..

[336] Edeleva M., De Smit K., Debrie S., Verberckmoes A., Marien Y.W., D’Hooge D.R. (2023). Molecular scale-driven upgrading of extrusion technology for sustainable polymer processing and recycling. Curr. Opin. Green Sustain. Chem..

[337] Wu W.-J., Sun X.-L., Chen Q., Qian Q. (2022). Recycled Poly(Ethylene Terephthalate) from Waste Textiles with Improved Thermal and Rheological Properties by Chain Extension. Polymers.

[338] Yang Z., Xin C., Mughal W., Li X., He Y. (2018). High-melt-elasticity poly(ethylene terephthalate) produced by reactive extrusion with a multi-functional epoxide for foaming. J. Appl. Polym. Sci..

[339] Ozmen S.C., Ozkoc G., Serhatli E. (2019). Thermal, mechanical and physical properties of chain extended recycled polyamide 6 via reactive extrusion: Effect of chain extender types. Polym. Degrad. Stab..

[340] Tuna B., Benkreira H. (2019). Reactive extrusion of polyamide 6 using a novel chain extender. Polym. Eng. Sci..

[341] Li J., Li B., Huang S., Luo S., He S., Gao C., Liu S. (2022). Epoxy chain extender grafted pyrophyllite/poly(ethylene terephthalate) composites with enhanced crystallinity and mechanical properties. Polym. Compos..

[342] Santos R.M., Costa A.R., Almeida Y.M., Carvalho L.H., Delgado J.M., Lima E.S., Magalhães H.L., Gomez R.S., Leite B.E., Rolim F.D. (2022). Thermal and Rheological Characterization of Recycled PET/Virgin HDPE Blend Compatibilized with PE-g-MA and an Epoxy Chain Extender. Polymers.

[343] Scremin D.M., Miyazaki D.Y., Lunelli C.E., Silva S.A., Zawadzki S.F. (2019). PET recycling by alcoholysis using a new heterogeneous catalyst: Study and its use in polyurethane adhesives preparation. Macromol. Symp..

[344] Berg D., Pich A., Möller M. (2018). Post-Consumer Poly (Ethylene Terephthalate)-Properties, Problems During Reprocessing, and Modification by Reactive Extrusion.

[345] Lee S.J., Hahm W.G., Kikutani T., Kim B.C. (2009). Effects of clay and POSS nanoparticles on the quiescent and shear-induced crystallization behavior of high molecular weight polyethylene terephthalate. Polym. Eng. Sci..

[346] Makkam S., Harnnarongchai W. (2014). Rheological and mechanical properties of recycled PET modified by reactive extrusion. Energy Procedia.

[347] Johnson S., Echeverria D., Venditti R., Jameel H., Yao Y. (2020). Supply Chain of Waste Cotton Recycling and Reuse: A Review. AATCC J. Res..

[348] Liu W., Liu S., Liu T., Liu T., Zhang J., Liu H. (2019). Eco-friendly post-consumer cotton waste recycling for regenerated cellulose fibers. Carbohydr. Polym..

[349] Ma Y., Zeng B., Wang X., Byrne N. (2019). Circular Textiles: Closed Loop Fiber to Fiber Wet Spun Process for Recycling Cotton from Denim. ACS Sustain. Chem. Eng..

[350] Regel. https://regel.world/.

[351] OBBOTEC-SPEX SPEX: Recycle Plastic by Dissolution. https://obbotec-spex.com/en/spex-technologie/.

[352] PureCycle. https://www.purecycle.com/.

[353] TeijinAramid. https://www.teijinaramid.com/en/sustainability.

[354] Antonini P.G., Palluau M., Safran C.H.B. (2020). Recyclage Chimique des Plastiques Application aux Plastiques Issus des DEEE. https://www.arbe-regionsud.org/Block/download/?id=175234&filename=ecosystem-recyclage-physique-des-deee-2020.pdf.

[355] Biswas M.C., Dwyer R., Jimenez J., Su H.-C., Ford E. (2021). Strengthening Regenerated Cellulose Fibers Sourced from Recycled Cotton T-Shirt Using Glucaric Acid for Antiplasticization. Polysaccharides.

[356] Liu R.-G., Shen Y.-Y., Shao H.-L., Wu C.-X., Hu X.-C. (2001). An Analysis of Lyocell Fiber Formation as a Melt–spinning Process. Cellulose.

[357] Li X., Zhang Y., Tang J., Lan A., Yang Y., Gibril M., Yu M. (2016). Efficient preparation of high concentration cellulose solution with complex DMSO/ILs solvent. J. Polym. Res..

[358] A Study on Technologies for Recycling and Re-Use of Textile Scraps. Tex-Med Alliances. https://www.enicbcmed.eu/sites/default/files/2023-01/A%20Study%20on%20technologies%20for%20recycling%20and%20re-use%20of%20textile%20scraps%20-%20TMA%20WP6.pdf.

[359] Triebert D., Hanel H., Bundt M., Wohnig K. (2021). Solvent-based recycling. Circular Economy of Polymers: Topics in Recycling Technologies.

[360] Cecon V.S., Da Silva P.F., Curtzwiler G.W., Vorst K.L. (2021). The challenges in recycling post-consumer polyolefins for food contact applications: A review. Resour. Conserv. Recycl..

[361] Eschenbacher A., Varghese R.J., Delikonstantis E., Mynko O., Goodarzi F., Enemark-Rasmussen K., Oenema J., Abbas-Abadi M.S., Stefanidis G.D., Van Geem K.M. (2022). Highly selective conversion of mixed polyolefins to valuable base chemicals using phosphorus-modified and steam-treated mesoporous HZSM-5 zeolite with minimal carbon footprint. Appl. Catal. B Environ..

[362] Mu B., Yang Y. (2022). Complete separation of colorants from polymeric materials for cost-effective recycling of waste textiles. Chem. Eng. J..

[363] Abbas-Abadi M.S., Haghighi M.N., Yeganeh H., McDonald A.G. (2014). Evaluation of pyrolysis process parameters on polypropylene degradation products. J. Anal. Appl. Pyrolysis.

[364] Wang L., Huang S., Wang Y. (2022). Recycling of Waste Cotton Textile Containing Elastane Fibers through Dissolution and Regeneration. Membranes.

[365] Sherwood J. (2020). Closed-loop recycling of polymers using solvents: Remaking plastics for a circular economy. Johns. Matthey Technol. Rev..

[366] Serad S.L. Polyester Dissolution for Polyester/Cotton Blend Recycle. https://patents.google.com/patent/US5342854A/en.

[367] Brinks G.J., Bouwhuis G.H., Agrawal P.B., Gooijer H. Processing of Cotton-Polyester Waste Textile. https://patents.google.com/patent/WO2014081291A1/en.

[368] WornAgain. https://wornagain.co.uk/.

[369] TextileChange. https://textilechange.com/.

[370] Sarian A.K., Handermann A.C., Jones S., Davis E.A., Adhya A. Recovery of Polyamides from Composite Articles. https://patents.google.com/patent/US5849804A/en.

[371] Haeggblom J., Budde I. (2021). Circular Design as a Key Driver for Sustainability in Fashion and Textiles. Sustainable Textile and Fashion Value Chains.

[372] ECOPET. https://www.ecopet.info/en/.

[373] Tournier V., Topham C.M., Gilles A., David B., Folgoas C., Moya-Leclair E., Kamionka E., Desrousseaux M.L., Texier H., Gavalda S. (2020). An engineered PET depolymerase to break down and recycle plastic bottles. Nature.

[374] Yousef S., Tatariants M., Tichonovas M., Sarwar Z., Jonuškienė I., Kliucininkas L. (2019). A new strategy for using textile waste as a sustainable source of recovered cotton. Resour. Conserv. Recycl..

[375] Haslinger S., Hummel M., Anghelescu-Hakala A., Määttänen M., Sixta H. (2019). Upcycling of cotton polyester blended textile waste to new man-made cellulose fibers. Waste Manag..

[376] De Silva R., Wang X., Byrne N. (2014). Recycling textiles: The use of ionic liquids in the separation of cotton polyester blends. RSC Adv..

[377] Jeihanipour A., Karimi K., Niklasson C., Taherzadeh M.J. (2010). A novel process for ethanol or biogas production from cellulose in blended-fibers waste textiles. Waste Manag..

[378] Xia G., Han W., Xu Z., Zhang J., Kong F., Zhang J., Zhang X., Jia F. (2021). Complete recycling and valorization of waste textiles for value-added transparent films via an ionic liquid. J. Environ. Chem. Eng..

[379] Abbas-Abadi M.S., Ureel Y., Eschenbacher A., Vermeire F.H., Varghese R.J., Oenema J., Stefanidis G.D., Van Geem K.M. (2023). Challenges and opportunities of light olefin production via thermal and catalytic pyrolysis of end-of-life polyolefins: Towards full recyclability. Prog. Energy Combust. Sci..

[380] Abbas-Abadi M.S., Zayoud A., Kusenberg M., Roosen M., Vermeire F., Yazdani P., Van Waeyenberg J., Eschenbacher A., Hernandez F.J.A., Kuzmanović M. (2022). Thermochemical recycling of end-of-life and virgin HDPE: A pilot-scale study. J. Anal. Appl. Pyrolysis.

[381] De Keer L., Kilic K.I., Van Steenberge P.H.M., Daelemans L., Kodura D., Frisch H., De Clerck K., Reyniers M.-F., Barner-Kowollik C., Dauskardt R.H. (2021). Computational prediction of the molecular configuration of three-dimensional network polymers. Nat. Mater..

[382] Zayoud A., Thi H.D., Kusenberg M., Eschenbacher A., Kresovic U., Alderweireldt N., Djokic M., Van Geem K.M. (2022). Pyrolysis of end-of-life polystyrene in a pilot-scale reactor: Maximizing styrene production. Waste Manag..

[383] Godiya C.B., Gabrielli S., Materazzi S., Pianesi M.S., Stefanini N., Marcantoni E. (2019). Depolymerization of waste poly (methyl methacrylate) scraps and purification of depolymerized products. J. Environ. Manag..

[384] Moens E.K.C., De Smit K., Marien Y.W., Trigilio A.D., Van Steenberge P.H.M., Van Geem K.M., Dubois J.-L., D’hooge D.R. (2020). Progress in Reaction Mechanisms and Reactor Technologies for Thermochemical Recycling of Poly(methyl methacrylate). Polymers.

[385] Dogu O., Eschenbacher A., John Varghese R., Dobbelaere M., D’Hooge D.R., Van Steenberge P.H.M., Van Geem K.M. (2023). Bayesian tuned kinetic Monte Carlo modeling of polystyrene pyrolysis: Unraveling the pathways to its monomer, dimers, and trimers formation. Chem. Eng. J..

[386] De Smit K., Marien Y.W., Van Geem K.M., Van Steenberge P.H.M., D’Hooge D.R. (2020). Connecting polymer synthesis and chemical recycling on a chain-by-chain basis: A unified matrix-based kinetic Monte Carlo strategy. React. Chem. Eng..

[387] Goshayeshi B., Kumar R., Wang Y., Varghese R.J., Roy S., Baruah B., Lemonidou A.A., Van Geem K.M. (2025). Enhancing Polystyrene Recycling: Temperature-Responsive of Pyrolysis in a Pilot-Scale Vortex Reactor. J. Anal. Appl. Pyrolysis.

[388] Kusenberg M., Zayoud A., Roosen M., Thi H.D., Abbas-Abadi M.S., Eschenbacher A., Kresovic U., De Meester S., Van Geem K.M. (2022). A comprehensive experimental investigation of plastic waste pyrolysis oil quality and its dependence on the plastic waste composition. Fuel Process. Technol..

[389] Abbas-Abadi M.S., Haghighi M.N., Yeganeh H. (2013). Evaluation of pyrolysis product of virgin high density polyethylene degradation using different process parameters in a stirred reactor. Fuel Process. Technol..

[390] Abbas-Abadi M.S., Haghighi M.N., Yeganeh H. (2012). The effect of temperature, catalyst, different carrier gases and stirrer on the produced transportation hydrocarbons of LLDPE degradation in a stirred reactor. J. Anal. Appl. Pyrolysis.

[391] Eschenbacher A., Goodarzi F., Varghese R.J., Enemark-Rasmussen K., Kegnæs S., Abbas-Abadi M.S., Van Geem K.M. (2021). Boron-Modified Mesoporous ZSM-5 for the Conversion of Pyrolysis Vapors from LDPE and Mixed Polyolefins: Maximizing the C2–C4 Olefin Yield with Minimal Carbon Footprint. ACS Sustain. Chem. Eng..

[392] Abbas-Abadi M.S. (2021). The effect of process and structural parameters on the stability, thermo-mechanical and thermal degradation of polymers with hydrocarbon skeleton containing PE, PP, PS, PVC, NR, PBR and SBR. J. Therm. Anal. Calorim..

[393] Monsores K.G.d.C., Silva A.O.d., Oliveira S.d.S.A., Rodrigues J.G.P., Weber R.P. (2019). Influence of ultraviolet radiation on polymethylmethacrylate (PMMA). J. Mater. Res. Technol..

[394] Abbas-Abadi M.S., Haghighi M.N., Yeganeh H. (2012). Effect of the melt flow index and melt flow rate on the thermal degradation kinetics of commercial polyolefins. J. Appl. Polym. Sci..

[395] Abudabbus M.M., Jevremović I., Nešović K., Perić-Grujić A., Rhee K.Y., Mišković-Stanković V. (2018). In situ electrochemical synthesis of silver-doped poly (vinyl alcohol)/graphene composite hydrogels and their physico-chemical and thermal properties. Compos. Part B Eng..

[396] Abbas-Abadi M.S., Van Geem K.M., Alvarez J., Lopez G. (2022). The pyrolysis study of polybutadiene rubber under different structural and process parameters: Comparison with polyvinyl chloride degradation. J. Therm. Anal. Calorim..

[397] Hamou K.B., Kaddami H., Dufresne A., Boufi S., Magnin A., Erchiqui F. (2018). Impact of TEMPO-oxidization strength on the properties of cellulose nanofibril reinforced polyvinyl acetate nanocomposites. Carbohydr. Polym..

[398] Ahire J.J., Neveling D.P., Dicks L.M.T. (2018). Polyacrylonitrile (PAN) nanofibres spun with copper nanoparticles: An anti-Escherichia coli membrane for water treatment. Appl. Microbiol. Biotechnol..

[399] Tamri Z., Yazdi A.V., Haghighi M.N., Abbas-Abadi M.S., Heidarinasab A. (2018). The effect of temperature, heating rate, initial cross-linking and zeolitic catalysts as key process and structural parameters on the degradation of natural rubber (NR) to produce the valuable hydrocarbons. J. Anal. Appl. Pyrolysis.

[400] Seifali Abbas-Abadi M., Nekoomanesh Haghighi M. (2017). The Consideration of Different Effective Zeolite Based Catalysts and Heating Rate on the Pyrolysis of Styrene Butadiene Rubber (SBR) in a Stirred Reactor. Energy Fuels.

[401] Salmasi S.S.Z., Abbas-Abadi M.S., Haghighi M.N., Abedini H. (2015). The effect of different zeolite based catalysts on the pyrolysis of poly butadiene rubber. Fuel.

[402] Lubna M.M., Salem K.S., Sarker M., Khan M.A. (2018). Modification of Thermo-Mechanical Properties of Recycled PET by Vinyl Acetate (VAc) Monomer Grafting Using Gamma Irradiation. J. Polym. Environ..

[403] Pramoda K.P., Liu T., Liu Z., He C., Sue H.-J. (2003). Thermal degradation behavior of polyamide 6/clay nanocomposites. Polym. Degrad. Stab..

[404] Trovati G., Sanches E.A., Neto S.C., Mascarenhas Y.P., Chierice G.O. (2010). Characterization of polyurethane resins by FTIR, TGA, and XRD. J. Appl. Polym. Sci..

[405] Zhou H., Long Y., Meng A., Chen S., Li Q., Zhang Y. (2015). A novel method for kinetics analysis of pyrolysis of hemicellulose, cellulose, and lignin in TGA and macro-TGA. Rsc Adv..

[406] Mofokeng J.P., Luyt A.S. (2015). Morphology and thermal degradation studies of melt-mixed poly(lactic acid) (PLA)/poly(ε-caprolactone) (PCL) biodegradable polymer blend nanocomposites with TiO_2_ as filler. Polym. Test..

[407] Denardin E.L., Samios D., Janissek P.R., de Souza G.P. (2001). Thermal degradation of aged chloroprene rubber studied by thermogravimetric analysis. Rubber Chem. Technol..

[408] Ye Q., Ma X., Li B., Jin Z., Xu Y., Fang C., Zhou X., Ge Y., Ye F. (2019). Development and Investigation of Lanthanum Sulfadiazine with Calcium Stearate and Epoxidised Soyabean Oil as Complex Thermal Stabilizers for Stabilizing Poly (vinyl chloride). Polymers.

[409] Yusof N., Ismail A. (2012). Post spinning and pyrolysis processes of polyacrylonitrile (PAN)-based carbon fiber and activated carbon fiber: A review. J. Anal. Appl. Pyrolysis.

[410] Seifali Abbas-Abadi M., Fathi M., Ghadiri M. (2019). Effect of Different Process Parameters on the Pyrolysis of Iranian Oak Using a Fixed Bed Reactor and TGA Instrument. Energy Fuels.

[411] Kumagai S., Yamasaki R., Kameda T., Saito Y., Watanabe A., Watanabe C., Teramae N., Yoshioka T. (2017). Tandem μ-reactor-GC/MS for online monitoring of aromatic hydrocarbon production via CaO-catalysed PET pyrolysis. React. Chem. Eng..

[412] Lin Y.-C., Cho J., Tompsett G.A., Westmoreland P.R., Huber G.W. (2009). Kinetics and mechanism of cellulose pyrolysis. J. Phys. Chem. C.

[413] Jomaa G., Goblet P., Coquelet C., Morlot V. (2015). Kinetic modeling of polyurethane pyrolysis using non-isothermal thermogravimetric analysis. Thermochim. Acta.

[414] Barnard E., Arias J.J.R., Thielemans W. (2021). Chemolytic depolymerisation of PET: A review. Green Chem..

[415] Quicker P., Seitz M., Vogel J. (2022). Chemical recycling: A critical assessment of potential process approaches. Waste Manag. Res..

[416] Filip D., Macocinschi D., Vlad S. (2011). Thermogravimetric study for polyurethane materials for biomedical applications. Compos. Part B Eng..

[417] Ge Y., Zhang Q., Zhang Y., Liu F., Han J., Wu C. (2018). High-performance natural rubber latex composites developed by a green approach using ionic liquid-modified multiwalled carbon nanotubes. J. Appl. Polym. Sci..

[418] Hanif M.U., Zwawi M., Algarni M., Bahadar A., Iqbal H., Capareda S.C., Hanif M.A., Waqas A., Hossain N., Siddiqui M.T.H. (2022). The effects of using pretreated cotton gin trash on the production of biogas from anaerobic co-digestion with cow manure and sludge. Energies.

[419] Shen F., Xiao W., Lin L., Yang G., Zhang Y., Deng S. (2013). Enzymatic saccharification coupling with polyester recovery from cotton-based waste textiles by phosphoric acid pretreatment. Bioresour. Technol..

[420] Venkatramanan V., Aravinth S., Prabhu C.S., Nithya M., Bama K.S. (2014). Bioethanol production from cotton waste using cellulase extracted from Fusarium species. Int. J. ChemTech Res..

[421] Wang Y., Zhao Y., Deng Y. (2008). Effect of enzymatic treatment on cotton fiber dissolution in NaOH/urea solution at cold temperature. Carbohydr. Polym..

[422] Vecchiato S., Skopek L., Jankova S., Pellis A., Ipsmiller W., Aldrian A., Mueller B., Herrero Acero E., Guebitz G.M. (2018). Enzymatic Recycling of High-Value Phosphor Flame-Retardant Pigment and Glucose from Rayon Fibers. ACS Sustain. Chem. Eng..

[423] Wojnowska-Baryła I., Bernat K., Zaborowska M. (2022). Strategies of recovery and organic recycling used in textile waste management. Int. J. Environ. Res. Public Health.

[424] Navone L., Speight R. (2018). Understanding the dynamics of keratin weakening and hydrolysis by proteases. PLoS ONE.

[425] Navone L., Moffitt K., Hansen K.-A., Blinco J., Payne A., Speight R. (2020). Closing the textile loop: Enzymatic fibre separation and recycling of wool/polyester fabric blends. Waste Manag..

[426] Quartinello F., Vecchiato S., Weinberger S., Kremenser K., Skopek L., Pellis A., Guebitz G.M. (2018). Highly selective enzymatic recovery of building blocks from wool-cotton-polyester textile waste blends. Polymers.

[427] Kuo C., Lin P., Lee C. (2010). Enzymatic saccharification of dissolution pretreated waste cellulosic fabrics for bacterial cellulose production by *Gluconacetobacter xylinus*. J. Chem. Technol. Biotechnol..

[428] Vasconcelos A., Cavaco-Paulo A. (2006). Enzymatic removal of cellulose from cotton/polyester fabric blends. Cellulose.

[429] Hong F., Guo X., Zhang S., Han S.-F., Yang G., Jönsson L.J. (2012). Bacterial cellulose production from cotton-based waste textiles: Enzymatic saccharification enhanced by ionic liquid pretreatment. Bioresour. Technol..

[430] Karbalaei S., Golieskardi A., Watt D.U., Boiret M., Hanachi P., Walker T.R., Karami A. (2019). Analysis and inorganic composition of microplastics in commercial Malaysian fish meals. Mar. Pollut. Bull..

[431] Kaabel S., Arciszewski J., Borchers T.H., Therien J.P.D., Friščić T., Auclair K. (2022). Solid-State Enzymatic Hydrolysis of Mixed PET/Cotton Textiles**. ChemSusChem.

[432] Szabo O.E., Csiszar E. (2017). Some factors affecting efficiency of the ultrasound-aided enzymatic hydrolysis of cotton cellulose. Carbohydr. Polym..

[433] Czernik S., Elam C.C., Evans R.J., Meglen R.R., Moens L., Tatsumoto K. (1998). Catalytic pyrolysis of nylon-6 to recover caprolactam. J. Anal. Appl. Pyrolysis.

[434] Jia H., Ben H., Luo Y., Wang R. (2020). Catalytic fast pyrolysis of poly (ethylene terephthalate)(PET) with zeolite and nickel chloride. Polymers.

[435] Zhang J., Gu J., Yuan H., Chen Y. (2021). Catalytic fast pyrolysis of waste mixed cloth for the production of value-added chemicals. Waste Manag..

[436] Lee S., Jung S., Lin K.-Y.A., Tsang Y.F., Kwon E.E. (2021). Use of CO_2_ and nylon as the raw materials for flammable gas production through a catalytic thermo-chemical process. Green Chem..

[437] Kaminsky W. (2021). Chemical recycling of plastics by fluidized bed pyrolysis. Fuel Commun..

[438] Akin O., Varghese R.J., Eschenbacher A., Oenema J., Abbas-Abadi M.S., Stefanidis G.D., Van Geem K.M. (2023). Chemical recycling of plastic waste to monomers: Effect of catalyst contact time, acidity and pore size on olefin recovery in ex-situ catalytic pyrolysis of polyolefin waste. J. Anal. Appl. Pyrolysis.

[439] Goshayeshi B., Alexandros Theofanidis S., Abbas-Abadi M.S., Mahmoudi E., Akin O., John Varghese R., Lemonidou A., Van Geem K.M. (2024). Selective catalytic conversion of model olefin and diolefin compounds of waste plastic pyrolysis oil: Insights for light olefin production and coke minimization. Chem. Eng. J..

[440] Dogu O., Pelucchi M., Van de Vijver R., Van Steenberge P.H.M., D’Hooge D.R., Cuoci A., Mehl M., Frassoldati A., Faravelli T., Van Geem K.M. (2021). The chemistry of chemical recycling of solid plastic waste via pyrolysis and gasification: State-of-the-art, challenges, and future directions. Prog. Energy Combust. Sci..

[441] Abbas-Abadi M.S., Kusenberg M., Zayoud A., Roosen M., Vermeire F., Madanikashani S., Kuzmanović M., Parvizi B., Kresovic U., De Meester S. (2023). Thermal pyrolysis of waste versus virgin polyolefin feedstocks: The role of pressure, temperature and waste composition. Waste Manag..

[442] Green A., Sadrameli S. (2004). Analytical representations of experimental polyethylene pyrolysis yields. J. Anal. Appl. Pyrolysis.

[443] De Smit K., Edeleva M., Trigilio A.D., Marien Y.W., Van Steenberge P.H.M., D’Hooge D.R. (2023). Kinetic Monte Carlo residence time distributions and kinetics in view of extrusion-based polymer modification and recycling. React. Chem. Eng..

[444] Milne B.J., Behie L.A., Berruti F. (1999). Recycling of waste plastics by ultrapyrolysis using an internally circulating fluidized bed reactor. J. Anal. Appl. Pyrolysis.

[445] Kusenberg M., Eschenbacher A., Djokic M.R., Zayoud A., Ragaert K., De Meester S., Van Geem K.M. (2022). Opportunities and challenges for the application of post-consumer plastic waste pyrolysis oils as steam cracker feedstocks: To decontaminate or not to decontaminate?. Waste Manag..

[446] Frączak D. (2021). Chemical Recycling of Polyolefins (PE, PP): Modern Technologies and Products. Waste Material Recycling in the Circular Economy-Challenges and Developments.

[447] Ouyang Y., Manzano M.N., Beirnaert K., Heynderickx G.J., Van Geem K.M. (2021). Micromixing in a gas–liquid vortex reactor. AIChE J..

[448] Lang X.J., Ouyang Y., Vandewalle L.A., Goshayeshi B., Chen S.Y., Madanikashani S., Perreault P., Van Geem K.M. (2022). Gas-solid hydrodynamics in a stator-rotor vortex chamber reactor. Chem. Eng. J..

[449] Orozco S., Alvarez J., Lopez G., Artetxe M., Bilbao J., Olazar M. (2021). Pyrolysis of plastic wastes in a fountain confined conical spouted bed reactor: Determination of stable operating conditions. Energy Convers. Manag..

[450] Pan X., Lian W., Yang J., Wang J., Zhang Z., Hao X., Abudula A., Guan G. (2022). Downer reactor simulation and its application on coal pyrolysis: A review. Carbon Resour. Convers..

[451] Luo H., Wang X., Liu X., Wu X., Shi X., Xiong Q. (2022). A review on CFD simulation of biomass pyrolysis in fluidized bed reactors with emphasis on particle-scale models. J. Anal. Appl. Pyrolysis.

[452] Moltó J., Font R., Conesa J.A. (2006). Study of the organic compounds produced in the pyrolysis and combustion of used polyester fabrics. Energy Fuels.

[453] Marco I.d., Caballero B., Torres A., Laresgoiti M.F., Chomon M.J., Cabrero M.A. (2002). Recycling polymeric wastes by means of pyrolysis. J. Chem. Technol. Biotechnol. Int. Res. Process Environ. Clean Technol..

[454] Kumagai S., Yamasaki R., Kameda T., Saito Y., Watanabe A., Watanabe C., Teramae N., Yoshioka T. (2020). Catalytic Pyrolysis of Poly (ethylene terephthalate) in the Presence of Metal Oxides for Aromatic Hydrocarbon Recovery Using Tandem μ-Reactor-GC/MS. Energy Fuels.

[455] Diaz-Silvarrey L.S., McMahon A., Phan A.N. (2018). Benzoic acid recovery via waste poly(ethylene terephthalate) (PET) catalytic pyrolysis using sulphated zirconia catalyst. J. Anal. Appl. Pyrolysis.

[456] Kwon D., Yi S., Jung S., Kwon E.E. (2021). Valorization of synthetic textile waste using CO2 as a raw material in the catalytic pyrolysis process. Environ. Pollut..

[457] Artetxe M., Lopez G., Amutio M., Elordi G., Bilbao J., Olazar M. (2013). Cracking of high density polyethylene pyrolysis waxes on HZSM-5 catalysts of different acidity. Ind. Eng. Chem. Res..

[458] Yousef S., Eimontas J., Striūgas N., Tatariants M., Abdelnaby M.A., Tuckute S., Kliucininkas L. (2019). A sustainable bioenergy conversion strategy for textile waste with self-catalysts using mini-pyrolysis plant. Energy Convers. Manag..

[459] Phan A.N., Ryu C., Sharifi V.N., Swithenbank J. (2008). Characterisation of slow pyrolysis products from segregated wastes for energy production. J. Anal. Appl. Pyrolysis.

[460] Kim S., Lee N., Lee S.W., Kim Y.T., Lee J. (2021). Upcycling of waste teabags via catalytic pyrolysis in carbon dioxide over HZSM-11. Chem. Eng. J..

[461] Lee S.B., Lee J., Tsang Y.F., Kim Y.-M., Jae J., Jung S.-C., Park Y.-K. (2021). Production of value-added aromatics from wasted COVID-19 mask via catalytic pyrolysis. Environ. Pollut..

[462] Jung S., Lee S., Dou X., Kwon E.E. (2021). Valorization of disposable COVID-19 mask through the thermo-chemical process. Chem. Eng. J..

[463] Wang M., Mao M., Zhang M., Wen G., Yang Q., Su B., Ren Q. (2019). Highly efficient treatment of textile dyeing sludge by CO_2_ thermal plasma gasification. Waste Manag..

[464] Athanasopoulos P., Zabaniotou A. (2022). Post-consumer textile thermochemical recycling to fuels and biocarbon: A critical review. Sci. Total Environ..

[465] Li S., Vela I.C., Järvinen M., Seemann M. (2021). Polyethylene terephthalate (PET) recycling via steam gasification–The effect of operating conditions on gas and tar composition. Waste Manag..

[466] Gholami Z., Gholami F., Tišler Z., Tomas M., Vakili M. (2021). A review on production of light olefins via fluid catalytic cracking. Energies.

[467] Dhaka A.K., Kaushal R., Pal Y. (2022). Assessing the power generation potential and quality of producer gas from blended of the cotton stalk and pistachio shell in an open core downdraft gasifier. Int. J. Ambient Energy.

[468] Abdpour S., Santos R.M. (2021). Recent advances in heterogeneous catalysis for supercritical water oxidation/gasification processes: Insight into catalyst development. Process Saf. Environ. Prot..

[469] Wu Y., Wen C., Chen X., Jiang G., Liu G., Liu D. (2017). Catalytic pyrolysis and gasification of waste textile under carbon dioxide atmosphere with composite Zn-Fe catalyst. Fuel Process. Technol..

[470] Jeong Y.-S., Kim J.-W., Ra H.W., Seo M.W., Mun T.-Y., Kim J.-S. (2022). Characteristics of Air Gasification of 10 Different Types of Plastic in a Two-Stage Gasification Process. ACS Sustain. Chem. Eng..

[471] Bai B., Liu Y., Zhang H., Zhou F., Han X., Wang Q., Jin H. (2020). Experimental investigation on gasification characteristics of polyethylene terephthalate (PET) microplastics in supercritical water. Fuel.

[472] Li W., Wanninayake N., Gao X., Li M., Pu Y., Kim D.-Y., Ragauskas A.J., Shi J. (2020). Mechanistic insight into lignin slow pyrolysis by linking pyrolysis chemistry and carbon material properties. ACS Sustain. Chem. Eng..

[473] Abbas-Abadi M.S., Van Geem K.M., Fathi M., Bazgir H., Ghadiri M. (2021). The pyrolysis of oak with polyethylene, polypropylene and polystyrene using fixed bed and stirred reactors and TGA instrument. Energy.

[474] Ghadiri M., Ghasemzadeh N., Behrooz Sarand A., Seifali Abbas Abadi M. (2024). Wastewater treatment by new high-performance activated carbon from Semecarpus Anacardium and Quercus Infectoria nutshells: Applications- kinetic and equilibrium studies. Iran. J. Chem. Chem. Eng..

[475] Bazgir H., Rostami M.R., Tavakkol S., Issaabadi Z., Shirazi H.M., Goshayeshi B., Van Geem K.M., Haghighi M.N., Abbas-Abadi M.S. (2023). The chemical process of producing activated carbon using walnut shells and plastic wastes. J. Therm. Anal. Calorim..

[476] Xu Z., Sun Z., Zhou Y., Chen W., Zhang T., Huang Y., Zhang D. (2019). Insights into the pyrolysis behavior and adsorption properties of activated carbon from waste cotton textiles by FeCl_3_-activation. Colloids Surf. A Physicochem. Eng. Asp..

[477] Akkouche F., Boudrahem F., Yahiaoui I., Vial C., Audonnet F., Aissani-Benissad F. (2021). Cotton textile waste valorization for removal of tetracycline and paracetamol alone and in mixtures from aqueous solutions: Effects of H_3_PO_4_ as an oxidizing agent. Water Environ. Res..

[478] Gumus H., Buyukkidan B. (2023). A Simple and Green Preparation Route of Waste Textile Based Photocatalytic Biochars for Pollution Removal. Chem. Afr..

[479] Parmakoğlu E.Ü., Çay A., Yanık J. (2023). Valorization of Solid Wastes from Textile Industry as an Adsorbent Through Activated Carbon Production. AATCC J. Res..

[480] Zou Z., Liu X., Ding J., Chen T., Wang X. (2020). Activated carbon powder derived from cashmere guard hair. J. Ind. Text..

[481] Deng H., Mao Z., Xu H., Zhang L., Zhong Y., Sui X. (2019). Synthesis of fibrous LaFeO_3_ perovskite oxide for adsorption of Rhodamine B. Ecotoxicol. Environ. Saf..

[482] Beyan S.M., Prabhu S.V., Sissay T.T., Getahun A.A. (2021). Sugarcane bagasse based activated carbon preparation and its adsorption efficacy on removal of BOD and COD from textile effluents: RSM based modeling, optimization and kinetic aspects. Bioresour. Technol. Rep..

[483] Keawploy N., Venkatkarthick R., Wangyao P., Zhang X., Liu R., Qin J. (2020). Eco-friendly conductive cotton-based textile electrodes using silver-and carbon-coated fabrics for advanced flexible supercapacitors. Energy Fuels.

[484] Nieto-Delgado C., Partida-Gutierrez D., Rangel-Mendez J.R. (2019). Preparation of activated carbon cloths from renewable natural fabrics and their performance during the adsorption of model organic and inorganic pollutants in water. J. Clean. Prod..

[485] Xu Z., Yuan Z., Zhang D., Chen W., Huang Y., Zhang T., Tian D., Deng H., Zhou Y., Sun Z. (2018). Highly mesoporous activated carbon synthesized by pyrolysis of waste polyester textiles and MgCl_2_: Physiochemical characteristics and pore-forming mechanism. J. Clean. Prod..

[486] Silva T.L., Cazetta A.L., Souza P.S.C., Zhang T., Asefa T., Almeida V.C. (2018). Mesoporous activated carbon fibers synthesized from denim fabric waste: Efficient adsorbents for removal of textile dye from aqueous solutions. J. Clean. Prod..

[487] Rabbi A., Dadashian F. (2019). Simultaneous improvement in tensile strength and adsorption capacity of activated carbon fibers during stabilization and activation of acrylic fibers. Diam. Relat. Mater..

[488] Kim J., Kwon W., Bai B.C., Jeong E. (2022). Recycling of cotton clothing into activated carbon fibers. Carbon Lett..

[489] Wanassi B., Hariz I.B., Ghimbeu C.M., Vaulot C., Hassen M.B., Jeguirim M. (2017). Carbonaceous adsorbents derived from textile cotton waste for the removal of Alizarin S dye from aqueous effluent: Kinetic and equilibrium studies. Environ. Sci. Pollut. Res..

[490] Karthik D., Baheti V., Militky J., Naeem M.S., Tunakova V., Ali A. (2021). Activated Carbon Derived from Carbonization of Kevlar Waste Materials: A Novel Single Stage Method. Materials.

[491] Williams P.T., Reed A.R. (2003). Pre-formed activated carbon matting derived from the pyrolysis of biomass natural fibre textile waste. J. Anal. Appl. Pyrolysis.

[492] Nahil M.A., Williams P.T. (2012). Surface chemistry and porosity of nitrogen-containing activated carbons produced from acrylic textile waste. Chem. Eng. J..

[493] Zhu X., Li Q., Qiu S., Liu X., Xiao L., Ai X., Yang H., Cao Y. (2016). Hard Carbon Fibers Pyrolyzed from Wool as High-Performance Anode for Sodium-Ion Batteries. JOM.

[494] Xu Z., Zhang D., Yuan Z., Chen W., Zhang T., Tian D., Deng H. (2017). Physicochemical and adsorptive characteristics of activated carbons from waste polyester textiles utilizing MgO template method. Environ. Sci. Pollut. Res..

[495] Yuan Z., Xu Z., Zhang D., Chen W., Huang Y., Zhang T., Tian D., Deng H., Zhou Y., Sun Z. (2018). Mesoporous activated carbons synthesized by pyrolysis of waste polyester textiles mixed with Mg-containing compounds and their Cr(VI) adsorption. Colloids Surfaces A Physicochem. Eng. Asp..

[496] Yuan Z., Xu Z., Zhang D., Chen W., Zhang T., Huang Y., Gu L., Deng H., Tian D. (2018). Box-Behnken design approach towards optimization of activated carbon synthesized by co-pyrolysis of waste polyester textiles and MgCl_2_. Appl. Surf. Sci..

[497] Xu Z., Tian D., Sun Z., Zhang D., Zhou Y., Chen W., Deng H. (2019). Highly porous activated carbon synthesized by pyrolysis of polyester fabric wastes with different iron salts: Pore development and adsorption behavior. Colloids Surfaces A Physicochem. Eng. Asp..

[498] Xia M., Shao X., Sun Z., Xu Z. (2020). Conversion of cotton textile wastes into porous carbons by chemical activation with ZnCl_2_, H_3_PO_4_, and FeCl_3_. Environ. Sci. Pollut. Res..

[499] Kumar S. (2022). Modeling PET Solvolysis.

[500] Kárpáti L., Fogarassy F., Kovácsik D., Vargha V. (2019). One-pot depolymerization and polycondensation of PET based random oligo-and polyesters. J. Polym. Environ..

[501] Pham D.D., Cho J. (2021). Low-energy catalytic methanolysis of poly (ethyleneterephthalate). Green Chem..

[502] Shirazimoghaddam S., Amin I., Faria Albanese J.A., Shiju N.R. (2023). Chemical Recycling of Used PET by Glycolysis Using Niobia-Based Catalysts. ACS Eng. Au.

[503] Štrukil V. (2021). Highly Efficient Solid-State Hydrolysis of Waste Polyethylene Terephthalate by Mechanochemical Milling and Vapor-Assisted Aging. ChemSusChem.

[504] Cao F., Wang L., Zheng R., Guo L., Chen Y., Qian X. (2022). Research and progress of chemical depolymerization of waste PET and high-value application of its depolymerization products. RSC Adv..

[505] Gupta P., Bhandari S. (2019). Chemical depolymerization of PET bottles via ammonolysis and aminolysis. Recycling of Polyethylene Terephthalate Bottles.

[506] Pegoretti A. (2021). Towards sustainable structural composites: A review on the recycling of continuous-fiber-reinforced thermoplastics. Adv. Ind. Eng. Polym. Res..

[507] Vollmer I., Jenks M.J., Roelands M.C., White R.J., van Harmelen T., de Wild P., van Der Laan G.P., Meirer F., Keurentjes J.T., Weckhuysen B.M. (2020). Beyond mechanical recycling: Giving new life to plastic waste. Angew. Chem. Int. Ed..

[508] Liu B., Fu W., Lu X., Zhou Q., Zhang S. (2018). Lewis Acid–Base Synergistic Catalysis for Polyethylene Terephthalate Degradation by 1,3-Dimethylurea/Zn(OAc)_2_ Deep Eutectic Solvent. ACS Sustain. Chem. Eng..

[509] Guo Z., Adolfsson E., Tam P.L. (2021). Nanostructured micro particles as a low-cost and sustainable catalyst in the recycling of PET fiber waste by the glycolysis method. Waste Manag..

[510] Jehanno C., Flores I., Dove A.P., Müller A.J., Ruipérez F., Sardon H. (2018). Organocatalysed depolymerisation of PET in a fully sustainable cycle using thermally stable protic ionic salt. Green Chem..

[511] Fang P., Liu B., Xu J., Zhou Q., Zhang S., Ma J., Lu X. (2018). High-efficiency glycolysis of poly(ethylene terephthalate) by sandwich-structure polyoxometalate catalyst with two active sites. Polym. Degrad. Stab..

[512] Scé F., Cano I., Martin C., Beobide G., Castillo Ó., de Pedro I. (2019). Comparing conventional and microwave-assisted heating in PET degradation mediated by imidazolium-based halometallate complexes. New J. Chem..

[513] Liu Y., Yao X., Yao H., Zhou Q., Xin J., Lu X., Zhang S. (2020). Degradation of poly(ethylene terephthalate) catalyzed by metal-free choline-based ionic liquids. Green Chem..

[514] Bin Jin S., Jeong J.-M., Son S.G., Park S.H., Lee K.G., Choi B.G. (2021). Synthesis of two-dimensional holey MnO_2_/graphene oxide nanosheets with high catalytic performance for the glycolysis of poly(ethylene terephthalate). Mater. Today Commun..

[515] Wang R., Wang T., Yu G., Chen X. (2020). A new class of catalysts for the glycolysis of PET: Deep eutectic solvent@ZIF-8 composite. Polym. Degrad. Stab..

[516] Lalhmangaihzuala S., Laldinpuii Z., Lalmuanpuia C., Vanlaldinpuia K. (2020). Glycolysis of Poly(Ethylene Terephthalate) Using Biomass-Waste Derived Recyclable Heterogeneous Catalyst. Polymers.

[517] Cano I., Martin C., Fernandes J.A., Lodge R.W., Dupont J., Casado-Carmona F.A., Lucena R., Cardenas S., Sans V., de Pedro I. (2020). Paramagnetic ionic liquid-coated SiO_2_@Fe_3_O_4_ nanoparticles—The next generation of magnetically recoverable nanocatalysts applied in the glycolysis of PET. Appl. Catal. B Environ..

[518] Fuentes C.A., Gallegos M.V., García J.R., Sambeth J., Peluso M.A. (2019). Catalytic Glycolysis of Poly (ethylene terephthalate) Using Zinc and Cobalt Oxides Recycled from Spent Batteries. Waste Biomass Valorization.

[519] Laldinpuii Z.T., Khiangte V., Lalhmangaihzuala S., Lalmuanpuia C., Pachuau Z., Lalhriatpuia C., Vanlaldinpuia K. (2022). Methanolysis of PET Waste Using Heterogeneous Catalyst of Bio-waste Origin. J. Polym. Environ..

[520] Du J.-T., Sun Q., Zeng X.-F., Wang D., Wang J.-X., Chen J.-F. (2020). ZnO nanodispersion as pseudohomogeneous catalyst for alcoholysis of polyethylene terephthalate. Chem. Eng. Sci..

[521] Jiang Z., Yan D., Xin J., Li F., Guo M., Zhou Q., Xu J., Hu Y., Lu X. (2022). Poly(ionic liquid)s as efficient and recyclable catalysts for methanolysis of PET. Polym. Degrad. Stab..

[522] Stanica-Ezeanu D., Matei D. (2021). Natural depolymerization of waste poly(ethylene terephthalate) by neutral hydrolysis in marine water. Sci. Rep..

[523] Kang M.J., Yu H.J., Jegal J., Kim H.S., Gil Cha H. (2020). Depolymerization of PET into terephthalic acid in neutral media catalyzed by the ZSM-5 acidic catalyst. Chem. Eng. J..

[524] Bäckström E., Odelius K., Hakkarainen M. (2021). Ultrafast microwave assisted recycling of PET to a family of functional precursors and materials. Eur. Polym. J..

[525] Karpati L., Fejer M., Kalocsai D., Molnar J., Vargha V. (2019). Synthesis and characterization of isophorondiamine based epoxy hardeners from aminolysis of PET. Express Polym. Lett..

[526] Nica S., Duldner M., Hanganu A., Iancu S., Cursaru B., Sarbu A., Filip P., Bartha E. (2018). Functionalized 1,5,7-triazabicyclo [4.4.0] dec-5-ene (TBD) as Novel Organocatalyst for Efficient Depolymerization of Polyethylene Terephthalate (PET) Wastes. Rev. Chim..

[527] Fukushima K., Lecuyer J.M., Wei D.S., Horn H.W., Jones G.O., Al-Megren H.A., Alabdulrahman A.M., Alsewailem F.D., McNeil M.A., Rice J.E. (2012). Advanced chemical recycling of poly(ethylene terephthalate) through organocatalytic aminolysis. Polym. Chem..

[528] Krall E.M., Klein T.W., Andersen R.J., Nett A.J., Glasgow R.W., Reader D.S., Dauphinais B.C., Mc Ilrath S.P., Fischer A.A., Carney M.J. (2014). Controlled hydrogenative depolymerization of polyesters and polycarbonates catalyzed by ruthenium (II) PNN pincer complexes. Chem. Commun..

[529] Feghali E., Cantat T. (2015). Room temperature organocatalyzed reductive depolymerization of waste polyethers, polyesters, and polycarbonates. ChemSusChem.

[530] Wu P., Lu G., Cai C. (2021). Cobalt–molybdenum synergistic catalysis for the hydrogenolysis of terephthalate-based polyesters. Green Chem..

[531] Kratish Y., Marks T.J. (2022). Efficient polyester hydrogenolytic deconstruction via tandem catalysis. Angew. Chem. Int. Ed..

[532] Kamimura A., Shiramatsu Y., Kawamoto T. (2019). Depolymerization of polyamide 6 in hydrophilic ionic liquids. Green Energy Environ..

[533] Patil D.B., Madhamshettiwar S.V. (2014). Kinetics and Thermodynamic Studies of Depolymerization of Nylon Waste by Hydrolysis Reaction. J. Appl. Chem..

[534] Wang L., Nelson G.A., Toland J., Holbrey J.D. (2020). Glycolysis of PET Using 1,3-Dimethylimidazolium-2-Carboxylate as an Organocatalyst. ACS Sustain. Chem. Eng..

[535] Shafaghat H., Lee H.W., Tsang Y.F., Oh D., Jae J., Jung S.-C., Ko C.H., Lam S.S., Park Y.-K. (2019). In-situ and ex-situ catalytic pyrolysis/co-pyrolysis of empty fruit bunches using mesostructured aluminosilicate catalysts. Chem. Eng. J..

[536] Choudhury K., Tsianou M., Alexandridis P. (2024). Recycling of Blended Fabrics for a Circular Economy of Textiles: Separation of Cotton, Polyester, and Elastane Fibers. Sustainability.

[537] Wu Y., Che Y., Wei X., Hu Q., Xu J., Guo B., Niu Z. (2024). Nondestructive Recovery of Cotton from Waste Polycotton Textiles by Catalytic Hydrolysis. ACS Sustain. Chem. Eng..

[538] Andini E., Bhalode P., Gantert E., Sadula S., Vlachos D.G. (2024). Chemical recycling of mixed textile waste. Sci. Adv..

[539] Shojaei B., Abtahi M., Najafi M. (2020). Chemical recycling of PET: A stepping-stone toward sustainability. Polym. Adv. Technol..

[540] Wu H.-S. (2021). Strategic possibility routes of recycled PET. Polymers.

[541] Thiyagarajan S., Maaskant-Reilink E., Ewing T.A., Julsing M.K., Van Haveren J. (2022). Back-to-monomer recycling of polycondensation polymers: Opportunities for chemicals and enzymes. RSC Adv..

[542] Ügdüler S., Van Geem K.M., Denolf R., Roosen M., Mys N., Ragaert K., De Meester S. (2020). Towards closed-loop recycling of multilayer and coloured PET plastic waste by alkaline hydrolysis. Green Chem..

[543] Zhang Y.H.P., Lynd L.R. (2004). Toward an aggregated understanding of enzymatic hydrolysis of cellulose: Noncomplexed cellulase systems. Biotechnol. Bioeng..

[544] Palme A., Peterson A., de la Motte H., Theliander H., Brelid H. (2017). Development of an efficient route for combined recycling of PET and cotton from mixed fabrics. Text. Cloth. Sustain..

[545] Yang Y., Lu Y., Xiang H., Xu Y., Li Y. (2002). Study on methanolytic depolymerization of PET with supercritical methanol for chemical recycling. Polym. Degrad. Stab..

[546] Fockink D.H., Maceno M.A.C., Ramos L.P. (2015). Production of cellulosic ethanol from cotton processing residues after pretreatment with dilute sodium hydroxide and enzymatic hydrolysis. Bioresour. Technol..

[547] Sanchis-Sebastiá M., Ruuth E., Stigsson L., Galbe M., Wallberg O. (2021). Novel sustainable alternatives for the fashion industry: A method of chemically recycling waste textiles via acid hydrolysis. Waste Manag..

[548] Trache D., Hussin M.H., Haafiz M.M., Thakur V.K. (2017). Recent progress in cellulose nanocrystals: Sources and production. Nanoscale.

[549] Kamimura A., Yamamoto S. (2007). An Efficient Method To Depolymerize Polyamide Plastics:  A New Use of Ionic Liquids. Org. Lett..

[550] Han M., Thomas S., Rane A., Kanny K., V.K A., Thomas M.G. (2019). 5—Depolymerization of PET Bottle via Methanolysis and Hydrolysis. Recycling of Polyethylene Terephthalate Bottles.

[551] Sinha V., Patel M.R., Patel J.V. (2010). Pet Waste Management by Chemical Recycling: A Review. J. Polym. Environ..

[552] Uekert T., Singh A., DesVeaux J.S., Ghosh T., Bhatt A., Yadav G., Afzal S., Walzberg J., Knauer K.M., Nicholson S.R. (2023). Technical, Economic, and Environmental Comparison of Closed-Loop Recycling Technologies for Common Plastics. ACS Sustain. Chem. Eng..

[553] Liu Q., Li R., Fang T. (2015). Investigating and modeling PET methanolysis under supercritical conditions by response surface methodology approach. Chem. Eng. J..

[554] Tang S., Li F., Liu J., Guo B., Tian Z., Lv J. (2022). MgO/NaY as modified mesoporous catalyst for methanolysis of polyethylene terephthalate wastes. J. Environ. Chem. Eng..

[555] Chan K., Zinchenko A. (2021). Conversion of waste bottles’ PET to a hydrogel adsorbent via PET aminolysis. J. Environ. Chem. Eng..

[556] Shukla S.R., Harad A.M. (2006). Aminolysis of polyethylene terephthalate waste. Polym. Degrad. Stab..

[557] Musale R.M., Shukla S.R. (2016). Deep eutectic solvent as effective catalyst for aminolysis of polyethylene terephthalate (PET) waste. Int. J. Plast. Technol..

[558] Metelski P.D. (2023). Chemical Recycle of PET. Industrial Arene Chemistry: Markets, Technologies, Sustainable Processes and Cases Studies of Aromatic Commodities.

[559] Radadiya R., Shahabuddin S., Gaur R. (2022). Waste to Best: Chemical Recycling of Polyethylene Terephthalate (PET) for Generation of Useful Molecules. Tailored Functional Materials: Select Proceedings of MMETFP 2021.

[560] Loccufier E., Debecker D.P., D’hooge D.R., De Buysser K., De Clerck K. (2024). Fibrous Material Structure Developments for Sustainable Heterogeneous Catalysis—An Overview. ChemCatChem.

[561] Kratish Y., Li J., Liu S., Gao Y., Marks T.J. (2020). Polyethylene Terephthalate Deconstruction Catalyzed by a Carbon-Supported Single-Site Molybdenum-Dioxo Complex. Angew. Chem..

[562] Ye M., Li Y., Yang Z., Yao C., Sun W., Zhang X., Chen W., Qian G., Duan X., Cao Y. (2023). Ruthenium/TiO_2_-Catalyzed Hydrogenolysis of Polyethylene Terephthalate: Reaction Pathways Dominated by Coordination Environment. Angew. Chem..

[563] Kumar A., von Wolff N., Rauch M., Zou Y.-Q., Shmul G., Ben-David Y., Leitus G., Avram L., Milstein D. (2020). Hydrogenative Depolymerization of Nylons. J. Am. Chem. Soc..

[564] Fernandes A.C. (2021). Reductive depolymerization as an efficient methodology for the conversion of plastic waste into value-added compounds. Green Chem..

[565] Monsigny L., Berthet J.-C., Cantat T. (2018). Depolymerization of Waste Plastics to Monomers and Chemicals Using a Hydrosilylation Strategy Facilitated by Brookhart’s Iridium(III) Catalyst. ACS Sustain. Chem. Eng..

[566] Balaraman E., Gnanaprakasam B., Shimon L.J.W., Milstein D. (2010). Direct Hydrogenation of Amides to Alcohols and Amines under Mild Conditions. J. Am. Chem. Soc..

[567] Koo H.J., Chang G.S., Kim S.H., Hahm W.G., Park S.Y. (2013). Effects of recycling processes on physical, mechanical and degradation properties of PET yarns. Fibers Polym..

[568] Gizem C., Gamze D.T., Fulya Y., Hassan I. (2022). Limitations of Textile Recycling: The Reason behind the Development of Alternative Sustainable Fibers. Next-Generation Textiles.

[569] Subramanian K., Chopra S.S., Cakin E., Li X.T., Lin C.S.K. (2020). Environmental life cycle assessment of textile bio-recycling—valorizing cotton-polyester textile waste to pet fiber and glucose syrup. Resour. Conserv. Recy..

[570] (2006). Environmental Management—Life Cycle Assessment—Principles and Framework.

[571] Shen L., Worrell E., Patel M.K. (2010). Open-loop recycling: A LCA case study of PET bottle-to-fibre recycling. Resour. Conserv. Recy..

[572] Fazio S., Castellani V., Sala S., Schau E.M., Secchi M., Zampori L., Diaconu E. (2018). Supporting Information to the Characterisation Factors of Recommended EF Life Cycle Impact Assessment Methods.

[573] Paunonen S., Kamppuri T., Katajainen L., Hohenthal C., Heikkila P., Harlin A. (2019). Environmental impact of cellulose carbamate fibers from chemically recycled cotton. J. Clean. Prod..

[574] Wernet G., Bauer C., Steubing B., Reinhard J., Moreno-Ruiz E., Weidema B. (2016). The ecoinvent database version 3 (part I): Overview and methodology. Int. J. Life Cycle Assess..

[575] Otto K.N., Wood K.L. (2003). Product Design: Techniques in Reverse Engineering and New Product Development.

[576] Varshney P., Swami C. Sustainable Apparel Design for Longevity: Review and Analysis, Proceedings of online International Conference on Fashion Apparel & Textile (INCFAT ‘22) (2022) 27. https://www.amity.edu/asft/pdf/INCFAT22-proceedings.pdf#page=42.

[577] (2012). Valuing Our Clothes: The True Cost of How We Design, Use and Dispose of Clothing in the UK.

[578] Niinimäki K., Peters G., Dahlbo H., Perry P., Rissanen T., Gwilt A. (2020). The environmental price of fast fashion. Nat. Rev. Earth Environ..

[579] Resortecs. https://resortecs.com/technology/.

[580] Wear2 The Circular Process. https://wear2.com/en/various/the-circular-process/.

[581] Laitala K., Klepp I.G. (2018). Care and Production of Clothing in Norwegian Homes: Environmental Implications of Mending and Making Practices. Sustainability.

[582] Arrigo E., Flavio G., Muthu S.S. (2024). Take-Back Programs for Fashion Brands’ Garments in Sustainable Manufacturing Systems. Sustainable Manufacturing Practices in the Textiles and Fashion Sector.

[583] Circular Business Models for Fashion and Textiles. Wrap. https://www.wrap.ngo/taking-action/textiles/actions/circular-business-models-fashion-textiles.

[584] Leonas K.K., Muthu S.S. (2017). The Use of Recycled Fibers in Fashion and Home Products. Textiles and Clothing Sustainability: Recycled and Upcycled Textiles and Fashion.

[585] Singh S., Jana P. (2024). Decoding the Science Behind the Chemical Recycling of Textiles. Functional Textiles and Clothing 2023.

